# Single-cell analysis reveals shared and distinct molecular signatures in brain organoid models of neurodegeneration and neuroinflammation

**DOI:** 10.1186/s13195-025-01926-0

**Published:** 2025-11-29

**Authors:** Sophie Le Bars, Mohamed Soudy, Sarah Louise Nickels, Jens Christian Schwamborn, Enrico Glaab

**Affiliations:** 1 Biomedical Data Science Group, Luxembourg Centre for Systems Biomedicine (LCSB), University of Luxembourg, Esch-sur-Alzette, Luxembourg; 2 Developmental and Cellular Biology Group, Luxembourg Centre for Systems Biomedicine (LCSB), University of Luxembourg, Esch-sur-Alzette, Luxembourg

**Keywords:** Neurodegeneration, Brain organoids, Single-cell transcriptomics, Alzheimer's disease, Parkinson's disease, Comparative transcriptomics, Cell-cell communication, Systems biology

## Abstract

**Background:**

Alzheimer’s disease (AD) and Parkinson’s disease (PD) are complex neurodegenerative disorders with common pathological features, but the molecular mechanisms underlying their early stages remain poorly understood. This study aims to elucidate common and divergent changes in cellular processes in the early stages of neurodegeneration and neuroinflammation using brain organoid models.

**Methods:**

We performed a multi-level comparative analysis of single-cell RNA sequencing data from brain organoid models previously designed to mimic specific features of PD- and AD-like pathology, by integrating gene expression, pathway enrichment, molecular network, and cell-cell communication analyses. Given the critical role of neuroinflammation in neurodegenerative disorders, we particularly focused on inflammatory signaling pathways and alterations in cell-cell communication that might drive disease progression.

**Results:**

Our results reveal both common and contrasting changes between the different organoid models, including a common dysregulation of apoptotic pathways in astrocytes, a common upregulation of energy metabolism pathways in neurons, and opposing trends in ribosome-related pathways. Notably, our multi-level analysis identified key inflammatory alterations, including contrasting changes mediated by *HMGB1* and shared dysregulation in the *MDK* signaling pathway. Finally, comparison with *post-mortem* brain tissue and GWAS data revealed a small set of overlapping significant genes, showing robust shared patterns across different stages of pathology and tissue sources.

**Conclusions:**

These findings provide new insights into the molecular basis of neurodegeneration and neuroinflammation, highlighting diverging and shared alterations between different organoid models and post-mortem brain tissues that may inform follow-up validation and preclinical intervention studies for neurodegenerative disorders.

**Supplementary Information:**

The online version contains supplementary material available at 10.1186/s13195-025-01926-0.

## Background

Neurodegenerative disorders such as Alzheimer’s disease (AD) and Parkinson’s disease (PD) are characterized by complex and heterogeneous molecular, cellular, and clinical features that pose significant challenges for the development of diagnostic and treatment approaches. Despite their distinct clinical presentations, these diseases share several common biological mechanisms, including neuroinflammation, dysregulation of iron homeostasis, mitochondrial dysfunction, oxidative stress, protein misfolding and neuronal loss [1]. Understanding the similarities and differences between neurodegenerative diseases at the molecular level can inform the development of more effective biomarker tools and therapeutic strategies for both diseases.

Recent advances in stem cell technology have enabled the development of brain organoids, three-dimensional in vitro models that recapitulate key aspects of human brain development and function [2]. These organoids provide a unique opportunity to study the early stages of neurodegenerative diseases in a human-relevant context, overcoming the limitations of animal models and *post-mortem* tissue studies [3, 4]. In addition, single-cell RNA sequencing (scRNA-seq) technology has improved our ability to dissect cellular heterogeneity and molecular changes with unprecedented resolution [5].

While several studies have used brain organoids to study AD [6] or PD [3] individually, a comprehensive comparison of early molecular changes across different organoid models of neurodegeneration and neuroinflammation has not been performed. Such a comparison can reveal common pathogenic mechanisms and diverging changes, and potentially provide pointers to new cross-disease therapeutic targets or disease-specific biomarkers.

In this study, we present a multi-level comparative analysis of two brain organoid models mimicking features of AD-like and PD-like neurodegeneration and neuroinflammation using scRNA-seq data. To provide a holistic view of the molecular landscape in the early stages of both diseases, we integrate gene expression, pathway enrichment, network, and cell-cell communication analyses. We focus these analyses on neurons and astrocytes, two cell types central to neurodegenerative processes in both diseases that are strongly represented in our single-cell data.

Our results reveal both common and distinct, cell type-specific molecular signatures in the two models, including a common dysregulation of apoptotic pathways in astrocytes, a common upregulation of energy metabolism pathways in neurons, and opposing trends in ribosome-related pathways. Notably, we identify robust contrasting alterations across multiple types of analyses in inflammatory signaling pathways, particularly those mediated by *HMGB1* and *MDK*, suggesting that shared pathways may be altered through different mechanisms in these models.

Overall, this comprehensive comparative analysis of transcriptomic alterations in key cell types associated with AD and PD provides new insights into converging and diverging cellular process changes. The findings may help pave the way towards a more complete mechanistic understanding of common and distinct molecular features between different neurodegenerative and neuroinflammatory conditions, potentially informing the development of more specific diagnostic approaches and early intervention strategies.

## Methods

### Single-cell RNA sequencing datasets

This study used two different scRNA-seq datasets derived from brain organoids for cross-disease comparison of molecular alterations in models of AD-like and PD-like pathology. 

#### Serum-induced AD-like pathology dataset (AD-like) dataset

This dataset was obtained from a study by Chen et al. [7] (GEO database ID: GSE164089). The brain organoids in this dataset were derived from healthy donor fibroblasts, and the experimental approach used serum treatment to model features of sporadic AD pathology by mimicking blood-brain barrier (BBB) dysfunction observed in aging and AD progression. Importantly, the original study by Chen et al. on this model showed that it recapitulates some of the key hallmarks of AD pathology beyond neuroinflammation, including amyloid-beta aggregation, tau phosphorylation, synaptic loss, and impaired neural networks [7]. We therefore term this model as “AD-like”, while acknowledging its shortcomings as a model that lacks the established AD-specificity of genetic models involving familial AD mutations and captures only a subset of AD pathology features, as discussed in detail in the ‘Limitations’ section of the manuscript. The brain organoids were generated from human cortical fibroblasts and cultured for 94–95 days before harvesting for scRNA-seq analysis. Single cell suspensions were prepared and processed using the 10x Genomics Chromium platform with a 10X V3 Single Cell 3’ Solution Kit. The experimental design included two samples exposed to a serum mimicking BBB dysfunction in AD and two control samples. The libraries were sequenced on the Illumina NovaSeq 6000 platform (see [7] for the detailed experimental procedures).

#### Parkinson’s disease-like (PD-like) dataset

This dataset was obtained from an in-house study by Nickels et al. (manuscript submitted). Brain organoids were derived from PD patients carrying the GBA-N409S mutation. These organoids were generated from human fibroblasts and cultured for 30 days before harvesting for scRNA-seq analysis. Single-cell RNA sequencing libraries were prepared and sequenced by Singleron Biotechnologies using their commercial GEXSCOPE™ platform. Sequencing depth was targeted at 50,000 reads per cell, generating approximately 90 GB of data per library. The experimental design included organoids derived from three healthy individuals and three PD patients with the GBA-N409S mutation.

Sample sizes were assessed based on previous scRNA-seq studies of brain organoids, which indicated that approximately 2,000–5,000 cells per condition provide sufficient statistical power (> 0.8) to detect differentially expressed genes with a minimum fold change of 1.5 at an FDR of 0.05. Accordingly, both datasets exceeded minimum sample size requirements for detecting biologically meaningful differences between conditions.

**Fig. 1:**
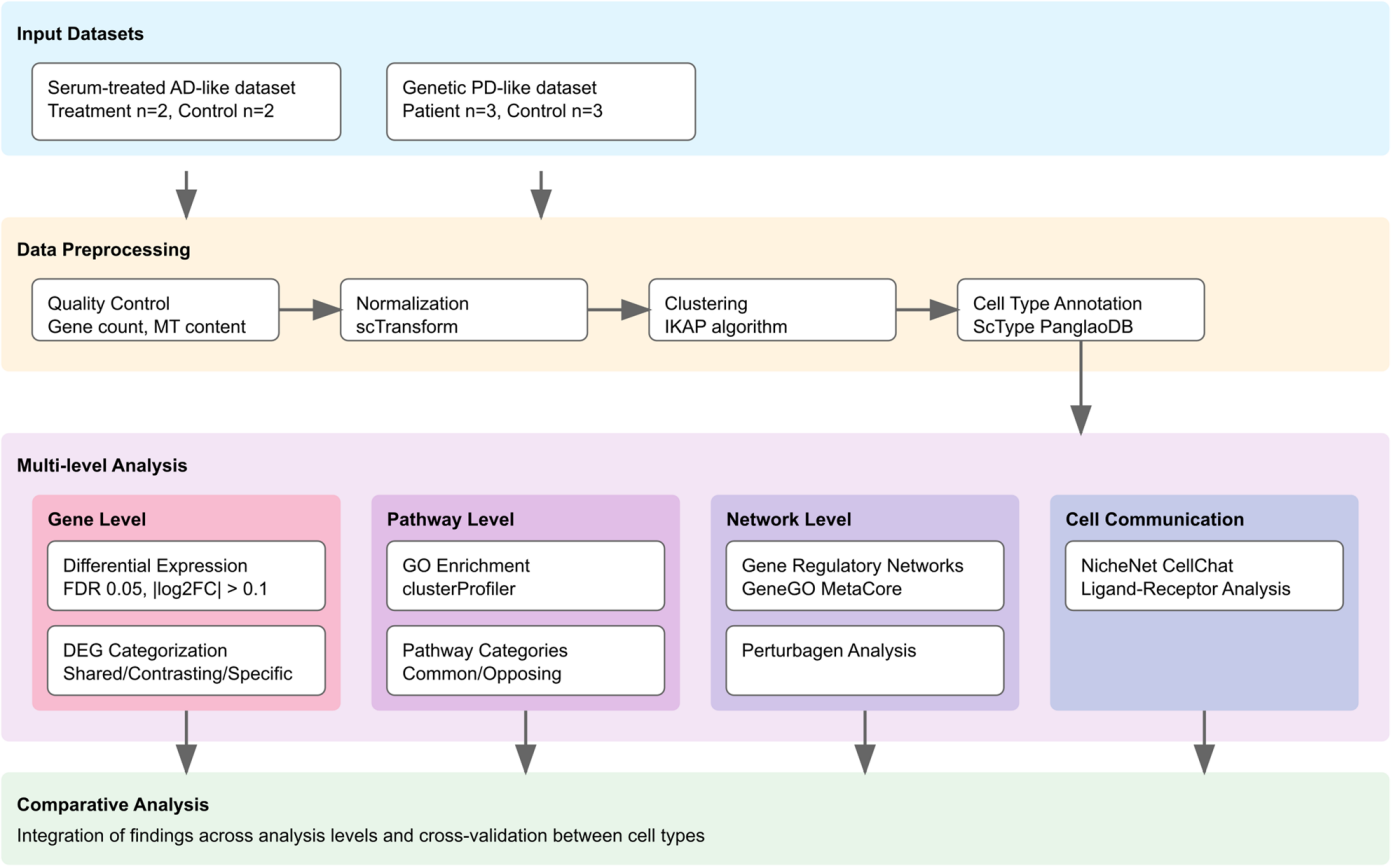
Overview of the study workflow. From top to bottom, the diagram shows the input datasets, the data preprocessing steps, the different multi-level omics analysis, and the final comparative analysis

A comparative overview of the main characteristics of these datasets is shown in Fig. 1. More detailed technical information on each dataset is provided in Supplementary Table 1.

### Addressing developmental time differences between datasets

We acknowledge that the substantial difference in organoid culture duration (30 days for the PD-like dataset vs. 94–95 days for the AD-like dataset) represents a methodological limitation that can confound cross-disease comparisons. This challenge is common in cross-disease comparative studies involving datasets with varying experimental protocols. We considered dataset integration and harmonization approaches (e.g., Harmony [8], Seurat CCA [9]) to address developmental stage differences but determined these methods are not appropriate for our study design. In our case, study identity, disease type, developmental stage, and experimental platform are completely interlinked, making it impossible to reliably separate these factors statistically. Harmonization algorithms assume that biological signals are conserved across datasets while only technical noise varies, but this assumption is not applicable when datasets represent fundamentally different biological states and experimental contexts. Therefore, we implemented the alternative analytical and filtering strategies described as follows.

First, our primary analytical approach compared disease vs. control samples within each dataset separately, thereby controlling for developmental stage and other dataset-specific factors within each comparison. All differential expression analyses were performed within-dataset (AD-like vs. matched control; PD-like vs. matched control) before comparing the resulting gene signatures between diseases. While this statistical framework enables meaningful comparisons by performing differential expression analysis within matched experimental contexts, we explicitly acknowledge that subsequent cross-dataset signature comparisons remain confounded by experimental differences including culture duration, protocols, and sequencing platforms, requiring cautious interpretation. This within-dataset approach followed by signature comparison represents the most appropriate analytical strategy given these inherent constraints, as it ensures statistical validity for within-dataset comparisons while supporting hypothesis discovery and prioritization of cross-disease similarities for further filtering of candidate disease genes (e.g., through comparison with DEGs from *post-mortem* brain studies and genes from GWAS meta-analyses, see below).

Second, we performed additional analyses to assess the potential impact of developmental timing on our results. We examined the expression patterns of known developmental markers and neuronal maturation genes across both datasets to characterize the developmental states represented. Additionally, using pathway enrichment analysis results, we confirmed that disease-associated signatures show minimal overlap with developmental pathways.

Third, we implemented a conservative interpretation framework, prioritizing findings that: (a) showed consistent patterns across multiple analytical levels (gene, pathway, network, and cell-cell communication), (b) demonstrated biological plausibility based on known disease mechanisms, and (c) showed significance in our *post-mortem* tissue or GWAS comparison analyses, which provide independent confirmation of disease relevance.

### Data pre-processing

Single-cell RNA sequencing data preprocessing was performed using the Seurat R package (version 5, RRID: SCR_016341) for both AD-like and PD-like datasets.

For the AD-like dataset, quality control was implemented by filtering cells based on gene count and mitochondrial gene percentage. Cells were retained if they met the following criteria: gene count between 200 and 8,000 and mitochondrial gene percentage below 5%. These thresholds were determined by following the Seurat tutorial workflow (see the ‘Guided tutorial’ section on https://satijalab.org/seurat/articles/get_started_v5_new), which uses violin and feature scatter plots to assess quality control (QC) metrics. The gene count range (200–8,000) removes empty droplets and doublets while retaining diverse cell types. The upper limit of this range was adjusted after manual inspection of the violin plots for the organoid data. In addition, as recommended in the tutorial, the 5% mitochondrial cutoff excludes cells with compromised integrity while preserving metabolically active neurons and astrocytes. Overall, this filtering resulted in 2,820 cells from AD-like brain organoids and 4,975 cells from control samples. Data normalization, scaling, and variance stabilization were performed using the *scTransform* function from the Seurat package.

For the PD-like dataset, using an analogous pre-processing in Seurat, the final processed data consisted of 85,624 cells, including 53,597 cells from PD-like brain organoids and 32,027 cells from control samples. It is important to note that within each individual dataset, all samples were processed as a single experimental batch and sequenced in a single run to minimize technical variation. Therefore, intra-dataset batch effects are not a concern, and batch correction within individual datasets was not necessary.

All data preprocessing and analysis steps were performed using the R programming language (version 4.4.0, RRID: SCR_001905). Computations were implemented on an RStudio (RRID: SCR_000432) server with 1 TB of memory to accommodate the large amount of cellular data.

### Clustering and cell-type annotation

Given the distinct experimental origins and validation contexts of our AD- and PD-like datasets, we employed dataset-appropriate clustering methodologies to ensure optimal biological accuracy while maintaining analytical rigor.

#### AD-like dataset - automated clustering approach

The AD-like dataset was clustered using the *Iterative K-means clustering with Adaptive Peaks* (IKAP) approach [9, 10]. This algorithm optimizes cluster identification automatically by iteratively varying the resolution and the number of top principal components (selected parameters: normalization method = “vst”, number of principal components = 7, clustering resolution = 1, k-parameter for SNN construction = 20, UMAP visualization: n.neighbors = 30 and min.dist = 0.3). This approach was selected because: (1) the data structure supported automated statistical clustering methods, as confirmed by internal cluster validity index assessment using the Silhouette width (avg. silhouette with = 0.429); (2) due to the public origin of the dataset, no prior experimental validation of cell type annotations was possible, requiring *de novo* cell type identification, and (3) established cell type marker databases provided robust reference standards for validation. Cell type annotation was then performed with the ScType method [11], using the Panglao database [12] for cell marker identification. The resulting cell type classifications were cross-referenced with those reported in the original publication [7] to ensure consistency and biological relevance. Cell type annotations were also cross-validated using published single-cell brain organoid datasets and established marker gene databases (CellMarker, PanglaoDB).

#### PD-like dataset - expert-guided semi-automated clustering

For the PD-like dataset, we used a semi-automated clustering approach guided by domain expertise from our collaborator Dr. Sarah Nickels, who performed direct experimental validation of clustering results. This methodology was chosen because: (1) for this in-house dataset, cell type identities were experimentally validated through in vitro expression analysis of specific gene markers, (2) expert knowledge of iPSC-derived neural differentiation patterns informed biologically appropriate cluster boundaries, and (3) automated clustering was not able to detect cluster structures reliably on the more complex PD-like dataset, necessitating manual cluster annotation and validation. Initial cell clusters were defined using the Seurat *FindClusters* algorithm with a resolution parameter of 0.2, following dimensionality reduction to the first 20 principal components. This clustering was manually annotated and refined according to the experimental marker validation results. Visualizations of cell type marker gene expression confirming the cell type annotations are presented in Suppl. Fig. S1. To facilitate comparison between the AD- and PD-like datasets, we merged subclusters into broader cell type categories. Specifically, all neuronal subclusters were merged into a single “Neurons” cluster. This step was necessary due to the differences in the specificity of detectable cell type clusters across the covered datasets. The validity of our annotation was further confirmed by visualizing the expression patterns of established astrocyte and neuron markers in both datasets (Supplementary Fig. S1, S2). To check for consistency between cell types in both datasets, we applied the same automatic annotation pipeline to the PD dataset and compared the results with the original annotations (see Results subsection “Cell Type Annotation”).

#### Cross-dataset analytical consistency and validation

Despite the required different clustering and annotation approaches, we ensured analytical compatibility through: (1) standardized quality control metrics applied to both datasets, (2) identical downstream analysis pipelines for differential expression and pathway analysis, (3) consistent statistical testing procedures, and (4) uniform data visualization and interpretation frameworks. Both clustering approaches were validated through silhouette analysis to assess cluster separation quality, marker gene enrichment analysis using established cell markers (databases: CellMarker, PanglaoDB).

### Gene-level analysis

Gene-level analysis was performed on neurons and astrocytes, the two cell types common to both organoid datasets. While mixed-effects models would be theoretically preferable for accounting for biological variability between samples, they are not practically feasible with our available data, as the limited sample size would hinder stable model convergence and reliable variance component estimation. Similarly, while pseudobulking would minimize Type I error, it would result in loss of cell-to-cell variability information and reduced statistical power and an expected increased Type II error, particularly for small sample sizes. Differential gene expression between disease-modeled brain organoids and their respective controls was therefore assessed using the Poisson method implemented in the software package *Seurat* [9]. P-values were adjusted for multiple hypothesis testing using the Bonferroni correction [9, 13]. Genes were considered significantly differentially expressed if they met the following criteria: false discovery rate (FDR) < 0.05 and absolute log2 fold change (|log2FC|) >0.1.

For the data interpretation, the differentially expressed genes (DEGs) were sub-categorized as follows:

1. *Shared DEGs*: Genes significantly differentially expressed in both diseases with concordant directionality (FDR < 0.05, |log2FC| >0.10 in both diseases, identical log2FC signs).
2. *Contrasting DEGs*: Genes significantly differentially expressed in both diseases with discordant directionality (FDR < 0.05, |log2FC| >0.10 in both diseases, opposite log2FC signs).
3. *Disease-specific DEGs*: Genes significantly differentially expressed in one disease (FDR < 0.05, |log2FC| >0.10), but not approaching significance in the other (nominal p-value > 0.5).

Subsequent analyses focused primarily on shared and contrasting DEGs, as these were the largest categories in our study.

### Pathway-level analysis

Pathway overrepresentation analyses were performed on the categorized differentially expressed genes (DEGs) defined in the previous section (shared, contrasting, and disease-specific DEGs in the comparison between the AD-like and PD-like organoid datasets). Additionally, we performed pathway enrichment analyses separately on the significant DEGs from each of the two independent brain organoid datasets, and separately for neurons and astrocytes. We applied the *enrichGO* function from the clusterProfiler R package (version 4.2.2, RRID: SCR_016884) [14], using human gene annotations from the org.Hs.eg.db R package (version 3.10.0). The analyses were carried out using four databases: WikiPathways, KEGG, Reactome and Gene Ontology (GO). For each input set, the top 100 enriched pathways were extracted from each database. Pathways were considered significantly overrepresented if they met the following criteria: adjusted p-value < 0.05 and a minimum of 3 mapped DEGs per pathway. Results specific to only one condition were reported separately for the AD-like data (Tables S5, S7, S9 and S11) and the PD-like data (Tables S6, S8, S10 and S12).

### Network-level analysis

Gene regulatory networks (GRNs) for the largest shared cell types, astrocytes, and neurons, were constructed using the identified shared and contrasting DEGs. Network construction and analysis were performed using GeneGO MetaCore software (RRID: SCR_008125). To identify potential key regulatory genes in these networks, we applied the algorithmic method described by Zickenrott et al. [15] to search for top candidate regulatory genes, whose modulation can revert the expression of a maximum number of DEGs within the network (called “perturbagens“).

Additional network analysis focused on identifying key nodes with outstanding network topological properties: (1) Hub nodes: Nodes with a high number of connections; (2) Central nodes: Nodes with strong connections within the network; (3) Attractor nodes: Nodes without parent nodes in the network. These topological features were considered alongside the perturbagene analysis to provide a comprehensive interpretation of the regulatory landscape in both disease contexts.

### Cell-cell communication analysis

To investigate common and distinct changes in intercellular communication between AD and PD in astrocytes and neurons, we used two complementary computational approaches: *NicheNet* (version 2.1.5) and *CellChat* (version 1.6.1, RRID: SCR_021946) [16, 17]. These methods allow the identification of ligand activities and hub genes involved in altered communication patterns.

First, we filtered the expression data for sender and receiver cells to include only genes expressed in more than 10% of cells to reduce noise and focus on robustly expressed genes. NicheNet analysis then predicted ligand activities and identified the most relevant ligands that could explain the observed gene expression changes in recipient cells.

Next, we applied CellChat to construct cell-cell communication networks and identify significantly overrepresented ligand-receptor pairs. We used the standard pathway database provided with CellChat, which contains known ligand-receptor interactions.

To identify biological processes affected by changes in cell-cell communication, we then performed pathway enrichment analysis. We used the intersection of target genes for the top predicted ligands (from NicheNet) and the significantly altered ligand-receptor pairs (from CellChat) as input. Enrichment analysis was performed using the *clusterProfiler* R package (version 4.2.2) with annotations from the GO database. An adjusted p-value < 0.05 was used as a significance threshold, and we restricted the analysis to biological process (BP) terms. For each cell type, we identified the five most significantly enriched pathways among those showing significant alterations in both the AD- and PD-like datasets. The complete ranking of enriched pathways is provided in the associated GitLab repository (see ‘Availability of Data and Materials’).

###  Comparison with post-mortem tissue data

To assess the relevance of our brain organoid findings to human disease pathology and compare the most robust results across different experimental settings, we performed an analysis of overlaps with DEGs identified in our previous study of *post-mortem* brain tissue in AD patients vs. controls and PD patients vs. controls [18]. Single-cell differential expression analysis for the *post-mortem* datasets was conducted using the Wilcoxon rank-sum test as implemented in the FindMarkers function of the Seurat R package (version 5.0). Statistical significance thresholds were applied consistently across both experimental systems: genes were considered significantly differentially expressed if they met false discovery rate (FDR) < 0.05 (Bonferroni correction) and absolute log2 fold change ≥ 0.1. The *post-mortem* analysis included the following datasets: AD *post-mortem* prefrontal cortex samples (71 AD subjects, 9 healthy controls) and PD *post-mortem* prefrontal cortex samples (6 PD subjects, 6 healthy controls), with cell type annotations validated using established marker genes from the CellMarker database [19]. As no significant differences between age and sex were detected between groups, no additional covariates beyond disease status were included in the differential expression models. The comparison between organoid and *post-mortem* data was conducted at two levels:

1. *Disease-Specific Overlap Analysis*: This analysis compared DEGs from the same modelled disease condition across different experimental systems (organoid vs. *post-mortem*). Specifically, we examined the overlap between significant DEGs identified in the organoid models versus *post-mortem* tissue separately for each disease (AD and PD) and cell type (neurons and astrocytes). This approach assesses whether a subset of alterations observed in organoid models can also be detected as disease-specific molecular signatures in a different experimental context, namely human *post-mortem* tissue. This analysis is not meant as a validation of DEGs, as the experimental settings and sample types are different across the datasets, but rather seeks to identify the most robust DEGs across diverse experimental settings, corroborating their disease-relevance. To quantify the overlap for each comparison, we used the following systematic approach:
  1. *DEG Collection*: We collected all significantly differentially expressed genes (adjusted p-value < 0.05) within neuronal and astrocyte clusters from both brain organoid datasets (AD-like and PD-like) and our corresponding *post-mortem* tissue datasets.
  2. *Intersection Identification*: For each cell type and disease combination, we identified the intersection of DEG sets between organoid and *post-mortem* datasets using gene symbols as identifiers.
  3. *Coverage Calculation*: We defined coverage as the proportion of organoid DEGs that were also significant in *post-mortem* tissue, using the formula: Coverage (%) = (Number of intersected DEGs / Total number of organoid DEGs) × 100.

This approach allows us to assess what proportion of organoid-identified disease signatures are also detectable in *post-mortem* tissue, providing insight into the degree to which organoid models capture molecular changes observed in human disease tissue. The coverage metric specifically indicates how many of the genes identified as dysregulated in organoids also show significant dysregulation in the corresponding post-mortem tissue analysis.

2) *Cross-Disease Signature Overlap Analysis: *This analysis focused on DEGs with significant changes in both AD-like and PD-like datasets, which we had previously categorized as either “shared” (dysregulated in the same direction in both AD-like and PD-like samples) or “contrasting” (dysregulated in opposite directions between AD-like and PD-like samples) according to the organoid data. We then examined whether these cross-disease signature DEGs were also present among the DEGs identified in *post-mortem* tissue studies. This more stringent analysis aims to identify robust gene markers that show consistent cross-disease patterns across distinct experimental systems. For this analysis, we maintained the same statistical threshold (FDR < 0.05) and coverage calculation approach as described above.

For both comparison levels, we also assessed the directionality of expression changes, comparing the sign of log2FC values between organoid and *post-mortem* data. Genes were considered to show consistent directionality only if their expression changed in the same direction (increase or decrease) in both datasets. This allowed us to perform two complementary types of overlap analysis to comprehensively assess molecular convergence between organoid and post-mortem systems: (1) *Total overlap analysis*, i.e., identifying all shared DEGs, providing insight into commonly dysregulated genes across experimental contexts; and (2) *directional overlap analysis*, i.e., genes were considered concordant only if both datasets showed either increased (positive log2FC) or decreased expression (negative log2FC) for the same gene. While the goal of the analysis was not to identify omics-scale associations but rather a small subset of genes with robust shared PD-associated patterns across diverse experimental systems, we also assessed the significance of the overlaps using the one-sided Fisher’s Exact test. All analyses were performed using R (version 4.4.0), and the associated custom scripts are available in our GitLab repository (see ‘Availability of data and materials’).

An important limitation of this cross-system comparison relates to differences in cell type resolution between datasets. The *post-mortem* tissue analysis included refined neuronal subtypes (excitatory and inhibitory neurons), whereas our organoid analysis collapsed all neurons into a single broad cluster due to the developmental stage and technical limitations of the organoid system. For comparison purposes, we aggregated the *post-mortem* neuronal subtypes into a combined “neurons” category to match the organoid cell type resolution. This approach, while necessary for comparison, inevitably obscures subtype-specific effects that may be present in the more mature *post-mortem* tissue. Similarly, astrocyte populations in *post-mortem* tissue may represent more mature and functionally diverse subtypes compared to the relatively immature astrocytes present in organoid cultures. When interpreting overlap statistics and directionality comparisons of gene expression alterations, these fundamental differences in cell type maturity and diversity need to be considered.

Finally, methodological differences between the studies may contribute to variations in overlap patterns. We applied the Poisson method for differential expression analysis in the organoid studies, which was necessitated by the limited sample sizes (2–3 donors per condition), whereas the Wilcoxon rank-sum test was used for the *post-mortem* studies, which had substantially larger sample sizes (71 AD subjects vs. 9 controls; 6 PD subjects vs. 6 controls). The Poisson method is more appropriate for scenarios with small sample sizes where pseudobulking approaches may not be feasible, while the Wilcoxon test can leverage the increased statistical power from larger sample sizes. These methodological differences, while each appropriate for their respective experimental constraints, may result in different sensitivities for detecting expression changes and could contribute to the modest overlap percentages observed.

### GWAS overlap analysis

To further assess the disease relevance of our organoid-derived DEGs, we systematically cross-referenced our findings with established genome-wide association study (GWAS) loci for AD and PD. For PD, we used the recent comprehensive meta-analysis compilation from Leonard et al. ([20], Table S3), including all genes harboring *bona fide* genome-wide significant SNPs (*p* < 5 × 10⁻⁸). For AD, we retrieved corresponding genes from the NIAGADS database compilation (https://advp.niagads.org/downloads) [21], which aggregates GWAS findings from multiple large-scale studies including recent meta-analyses. We identified the overlaps between GWAS genes and our organoid DEGs using exact gene symbol matching, including only genes showing both genome-wide significant genetic association and significant differential expression in our organoid models (FDR < 0.05, |log2FC| >0.1) and separating overlap analyses by disease and cell type.

For each cell type and disease combination, the significance of the overlap between GWAS genes and organoid DEGs was assessed using Fisher’s exact test. Background gene sets for statistical testing included the intersections of all genes considered in the relevant disease- and cell type-specific differential expression analyses and in the disease-specific GWAS datasets.

## Results

### Cell-type annotation

**Fig. 2:**
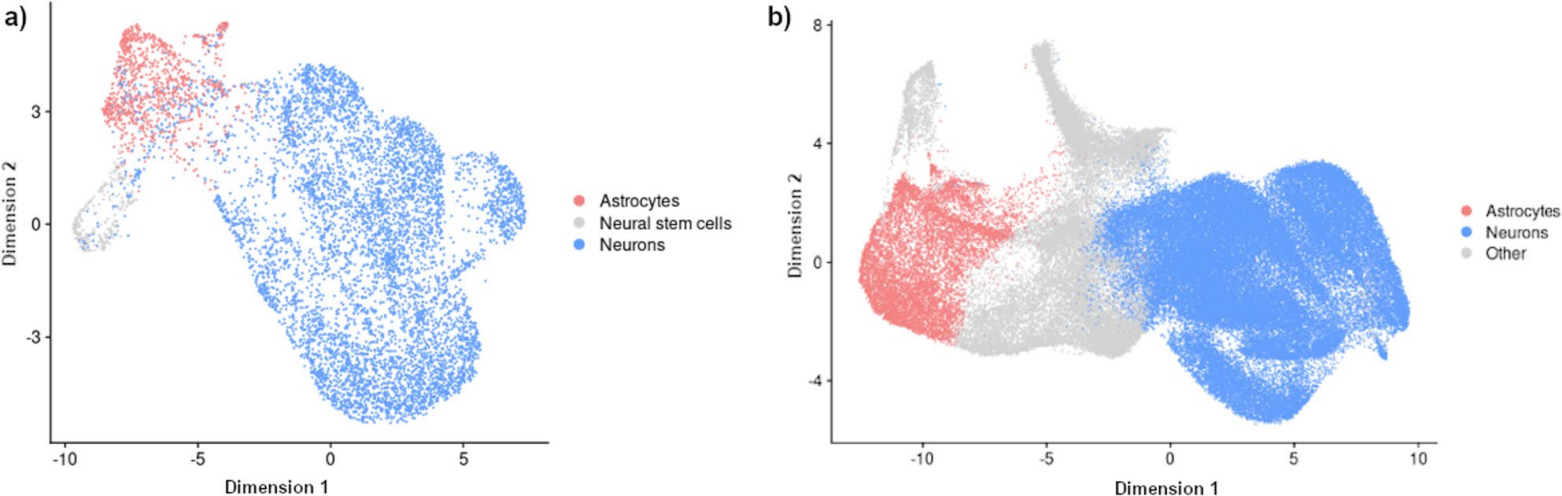
Two-dimensional visualization of cell type clustering and annotation in AD-like and PD-like brain organoid datasets. **a** UMAP (Uniform Manifold Approximation and Projection) visualization of cell clusters in the AD-like brain organoid dataset, showing three main cell populations: astrocytes (red), neural stem/precursor cells (gray), and neurons (blue). **b** UMAP visualization of cell clusters in the PD-like brain organoid dataset, revealing distinct populations of astrocytes (red), neurons (blue), and other cell types (gray). Both visualizations demonstrate clear separation between major cell types, with neurons forming the largest population in both datasets. The dimensional reduction was performed using principal component analysis followed by UMAP projection to enable visualization of the high-dimensional single-cell transcriptomic data

Cell-type clustering and annotation revealed three main clusters in the AD-like dataset, representing astrocytes, neurons, and neural stem/precursor cells. For the PD-like dataset, cell type annotations were assigned based on differential expression patterns of established lineage-specific markers from the database PanglaoDB (Franzén et al., 2019). These fine-scale annotations and clusters were then merged into larger clusters representing broader cell type annotation to enable a comparison with the AD-like dataset. (i.e., all neuronal clusters were merged into one cluster called “Neurons”). This allowed us to obtain two comparable cell type clusters for neurons and astrocytes (see Fig. 2), shared between both datasets, which are discussed separately in the following analyses.

To confirm the consistency of the obtained clusters and demonstrate their comparability despite different annotation processes being used, we applied the same fully automated pipeline used for the AD-like data also to the PD-like dataset. We then compared the broad clusters with those obtained from the original annotation of this dataset, observing the same coarse-grained clusters, with 89.3% overlap between the semi-automated and fully automated approaches in the PD dataset. Although the semi-automated method identifies more specific neuronal subtypes, the AD dataset contains only a single broad neuronal cluster, necessitating the merging of subtypes for comparison. While this precludes analysis of excitatory versus inhibitory neuron-specific effects, it allows us to identify broader neuronal dysfunction patterns shared across both datasets. Nevertheless, we acknowledge that it is possible that excitatory versus inhibitory differentiation was present in the AD dataset but not the PD dataset, and note this as a limitation. Importantly, our analysis confirms that the 30-day PD organoids contain well-defined neuronal populations rather than being dominated by neural stem cells, as neural stem cells are grouped together with other cell types in a separate cluster (labelled “Other”) while neurons form their own distinct cluster (labelled “Neuron”) that is specific to markers for mature neuronal cells.

### Comparative quality control analysis across datasets

While each individual dataset was processed as a single experimental batch (eliminating intra-dataset batch effects), the methodological differences between our AD-like and PD-like organoid datasets require comprehensive quality control analyses to assess dataset comparability and potential technical confounds that may influence cross-disease comparisons.

#### Technical Metrics Comparison

Side-by-side analysis confirmed significant differences in technical metrics between datasets (Suppl. Fig S11). The AD-like dataset (Day 94–95, *n* = 7,795 cells) showed a median of 6,544 UMIs per cell (IQR: 5,531-7,687.5) and 2,755 genes per cell (IQR: 2,056.5-3,329), while the PD-like dataset (Day 30, *n* = 85,624 cells) demonstrated higher sequencing depth with a median of 3,241 UMIs per cell (IQR: 2,870-3,750) and 1,592 genes per cell (IQR: 1,201-1,976). The PD dataset detected 27,851 unique genes compared to 18,413 in the AD-like dataset. Mitochondrial read percentages also differed: AD-like organoids showed 3.12% ± 1.29% mitochondrial reads while PD organoids showed 4.20% ± 3.62%, potentially reflecting different cellular stress states or technical processing differences.

#### Cell type resolution differences

The ten-fold difference in cell numbers (7,795 vs. 85,624) resulted in substantially different statistical power for cell type identification and differential gene expression analysis. The PD dataset resolved 9 distinct cellular clusters compared to 3 in the AD-like dataset, potentially reflecting either genuine biological differences or the enhanced resolution afforded by larger sample sizes.

#### Impact on downstream analyses

These technical differences have implications for cross-dataset comparisons. The higher sequencing depth in PD organoids provides greater sensitivity for detecting lowly expressed genes and identifying subtle expression changes. The different developmental time points (Day 30 vs. Day 94–95) represent different biological states, with Day 94–95 organoids showing enhanced neuronal maturation markers and reduced proliferation signatures compared to Day 30 organoids. In the following, we therefore conducted dedicated analyses for developmental genes and maturation markers.

### Assessment of developmental stage differences

To evaluate the potential impact of different organoid maturation times on our cross-disease comparisons, we examined the expression patterns of established developmental and neuronal maturation markers. Analysis of key developmental genes (*PAX6*, *FABP7*, *HES1*) and synaptic maturation markers (*SNAP25*, *MAP2*) revealed expected differences in maturation state between the 30-day PD and 94-95-day AD-like organoids (Supplementary Fig. S7 and S8). Importantly, Day 30 neurons expressed established mature neuronal markers including *MAP2* and *NEFL*, while showing minimal expression of proliferation markers such as *MKI67*, *TOP2A*, and *PCNA* (Supplementary Fig. S8), indicating that neurons in the 30-day organoids have exited the cell cycle and achieved a post-mitotic state suitable for comparative transcriptomic analysis. In addition, to gain a broader overview of ongoing development processes, we also extracted all genes associated with the functional term ‘Nervous system development’ in the Gene Ontology (GO) and BrainSpan databases and checked for overlaps with DEGs from our brain organoids study. All DEGs that overlap with this list of developmental genes are marked with an asterisk (*) throughout the organoid model DEG ranking tables (referenced in the supplementary tables ST19, ST20, ST24, and ST25) and should therefore be interpreted with caution, because while their within-dataset expression alterations are not confounded by developmental stage influences, their cross-dataset differences may reflect differences in developmental stages. Despite the developmental time difference between the organoid datasets, several observations support the validity of our cross-disease comparative approach: First, both datasets contained clearly identifiable and functionally similar populations of neurons and astrocytes, as confirmed by expression of cell-type-specific markers (Supplementary Fig. S1), indicating that both time points represent biologically relevant early developmental stages with comparable cell type composition. Second, in our pathway enrichment analyses, developmental and maturation-related processes showed no overrepresentation among the top-ranked disease-associated processes (see the top-ranked pathways in Suppl. Tab. S5 to S12; and ‘Availability of data and material’ for the online repository providing the complete pathway rankings). Third, our most robust findings (particularly those present also in *post-mortem* tissue) involved genes and pathways with established roles in neurodegeneration rather than development, suggesting genuine disease-relevant signals that overcome developmental confounds.

### Clustering approach validation

To confirm that our dataset-specific clustering approaches produce biologically coherent results, we performed comprehensive validation analyses across both datasets.

#### Marker gene expression validation

Both clustering approaches successfully identified biologically relevant cell types with appropriate marker gene expression patterns. In the AD-like dataset, automated clustering produced distinct cell populations expressing canonical markers that were confirmed for the main cell types of interest overlapping between the AD- and PD-like dataset, namely neurons (*STMN2*, *SNAP25*, *CAMK2N1*, see Suppl. Fig S1) and astrocytes (*SLC1A3*, *SOX9*, *FABP7*, see Suppl. Figure 2). Similarly, the expert-guided PD dataset clustering identified comparable cell types with validated marker expression (see Suppl. Figures 1 and 2).

#### Functional pathway coherence

Examination of the pathway enrichment results across both AD- and PD-like datasets (Suppl. Tab. S5 to S12) confirms functionally coherent patterns that support our cell type clustering approach.

*Neuronal clusters* showed enrichment for processes characteristic of neuronal function. Neurons in the AD-like dataset were significantly enriched for mitochondrial processes including “Mutation Caused Aberrant Abeta to Electron Transfer in Complex IV” (*p* = 5.67E-07) and “Respiratory Electron Transport” (*p* = 2.67E-11). PD neurons showed enrichment for “Mutation Caused Aberrant Abeta to Electron Transfer in Complex I” (*p* = 5.62E-11) and neuronal signaling pathways including “mGluR5 Ca2 + Apoptotic Pathway” and “VGCC Ca2 + Apoptotic Pathway,” which are specifically related to synaptic calcium signaling and neurotransmission.

*Astrocytic clusters* demonstrated enrichment for metabolic processes typical of glial support functions. Astrocytes in the AD-like dataset were significantly enriched for lipid metabolism pathways including “Mevalonate Pathway” (*p* = 2.76E-05) and “Cholesterol Biosynthesis” (*p* = 4.88E-05). PD astrocytes showed enrichment for “Mitochondrial Complex UCP1 in Thermogenesis” (*p* = 4.48E-05) and “Electron Transfer in Complex I” (*p* = 5.77E-05), reflecting their role in metabolic support and energy production.

These pathway enrichment patterns support the biological validity of our cell type annotations, as each cluster shows enrichment for processes matching their expected functional roles: neurons for synaptic signaling and specialized mitochondrial functions, astrocytes for metabolic support and lipid homeostasis, confirming that our clustering captured genuine cell-type-appropriate molecular signatures.

#### Silhouette analysis

Cluster separation quality was assessed using silhouette analysis, showing strong separation in both final clustered datasets (AD-like dataset mean silhouette width = 0.429; PD dataset = 0.39), indicating well-defined cluster boundaries regardless of clustering methodology.

These validation analyses confirm that both clustering approaches identify coherent and biologically relevant cell populations, supporting the validity of our comparative analyses despite methodological differences.

### Cellular composition and experimental condition distribution

To ensure that our identified cell clusters reflect biological diversity rather than technical artifacts, we performed comprehensive quantitative analysis of cellular composition and experimental condition distribution across all clusters.

#### AD-like dataset composition analysis

The AD-like dataset comprised 7,795 cells distributed across 3 major cell types (Suppl. Table S2). Control conditions contributed 4,975 cells (64.0%) while serum-treated conditions contributed 2,820 cells (36.0%). Across individual clusters, the proportion of control vs. treated cells ranged from 35% to 63%, demonstrating balanced representation without extreme skewing toward either condition (Suppl. Figure S4, left). Chi-squared analysis confirmed that the distribution of experimental conditions across clusters did not significantly deviate from expected random distribution (χ² = 0.99, df = 2, *p* = 0.61).

#### PD dataset composition analysis

The PD dataset contained 85,624 cells across 3 major cell types (Suppl. Table S3). Control iPSC-derived organoids contributed 32,027 cells (37%) while GBA-mutant organoids contributed 53,597 cells (63%). The distribution across clusters showed similar balance, with individual cluster compositions ranging from.

32% to 68% for each condition (Suppl. Figure S4, right). Chi-squared testing showed that cell type distribution differs significantly between HC and PD (χ² = 343.86, df = 2, *p* < 2.2e-16; PD vs. Control ratios: astrocytes: 6493/3002 = 2.16, neurons: 33884/19514 = 1.74, other: 13220/9511 = 1.39). For this reason, we analyzed PD vs. Control differences separately for each cell type.

#### Cross-cluster validation

Within each major cell type, we observed consistent representation of experimental conditions across subclusters. For example, in the AD-like dataset, astrocyte clusters showed control/treated ratios ranging from 1.64 to 1.86, while neuronal clusters ranged from 0.46 to 0.72. This consistency across hierarchical clustering levels further confirms that our clustering captures biological cell state variation rather than condition-specific technical effects.

Overall, these analyses indicate that our clustering approach captures biological cell type diversity with balanced representation of experimental conditions, supporting the validity of subsequent differential expression and pathway analyses.

### Gene expression analysis

#### Total dataset analysis (all cell types combined)

Differential expression analysis revealed substantial transcriptomic changes across both datasets. In the AD-like dataset, we identified a total of 1479 DEGs across all cell types, with a higher number observed in neurons (1061 genes) than in astrocytes (418 genes). Similarly, the PD dataset showed 3115 total DEGs, and among the largest cell type clusters, neurons exhibited more extensive changes (1033 genes) than astrocytes (872 genes). The magnitude and distribution of these changes are visualized in volcano plots for each cell type comparison (Suppl. Fig. S5). Cross-dataset comparison revealed 496 genes with overlapping differential expressions between AD-like and PD-like conditions across all cell types, representing 17.2% of total DEGs identified. Venn diagram analysis (Figure S6) demonstrates that neurons showed a larger overlap (320 shared genes) compared to astrocytes (176 shared genes).

#### Cell type-specific analysis

Next, we focused our analysis on the cell-type specific shared and contrasting DEGs between AD- and PD-like datasets, as the main goal of this study was to identify and interpret the converging and diverging pathways between the two diseases. In astrocytes, we found 46 shared and 130 contrasting DEGs, while in neurons we identified 142 shared and 178 contrasting DEGs. In Table 1, we present the top 10 most significant DEGs for each combination of cell types and categories of changes (shared DEGs or contrasting DEGs). The complete lists of DEGs for both datasets are provided in the GitLab repository associated with this study (see ‘Availability of Data and Materials’). The magnitude and statistical significance of expression changes are illustrated in volcano plots (Figures S5), highlighting the most informative genes (FDR < 0.05, |log2FC| >1). This filtering approach by significance and effect size threshold enables us to focus on DEGs with substantial expression changes that are more likely to reflect biologically meaningful alterations rather than technical variation.

Among the top 10 shared or contrasting DEGs in both neurons and astrocytes, we find the following groups of gene functions that have previously been implicated in neurodegenerative diseases:

**Table 1:**
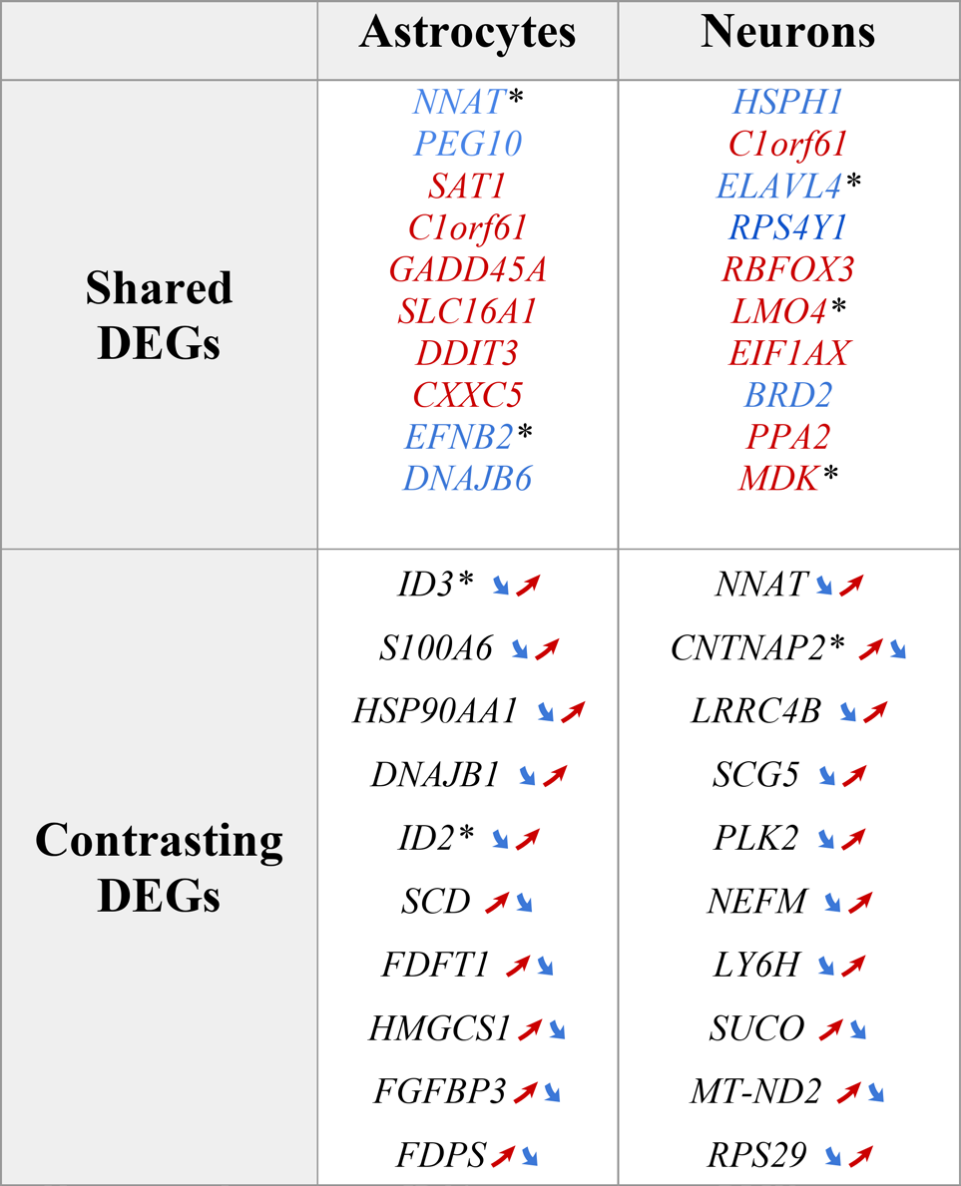
Overview of key DEGs in AD- and PD-like datasets, grouped by cell type and shared or contrasting changes

Overview of the most significant differentially expressed genes (DEGs) identified in AD- and PD-like organoid datasets, categorized by cell type (neurons and astrocytes) and the type of changes (shared or contrasting between AD- and PD-like models). DEGs are highlighted in blue for decreased expression and in red for increased expression. For the contrasting DEGs with opposite directionality, arrows indicate whether their expression increases (↗) or decreases (↘) in PD-like (left arrow) and in AD-like conditions (right arrow). All genes marked with an asterisk (*) are implicated in developmental processes from the GO or brainspan databases and May partly reflect developmental stage differences between datasets

1. *Inflammatory Response:*
  Astrocytes: HSP90AA1, DNAJB1, ID2, ID3, GADD45A, S100A6.
  Neurons: *MDK*, *C1ORF61/CROC4*.
  These genes are key regulators of inflammatory processes through diverse mechanisms. Heat shock proteins (*HSP90AA1*, *DNAJB1*) modulate inflammatory responses via NF-κB signaling [22, 23]. The ID family proteins (*ID2*, *ID3*) regulate cytokine production and immune cell responses [24–27], and *GADD45A* influences inflammatory signaling through MAPK pathways [28]. *S100A6* acts as a damage-associated molecular pattern protein (DAMP), triggering inflammatory responses through interaction with the receptor RAGE [29]. In neurons, *MDK* plays a dual role by mediating immune cell recruitment and modulating chemokine expression [30, 31], while *C1ORF61/CROC4* influences inflammatory gene expression through FOS signaling [32, 33]. The dysregulation of these inflammatory mediators in both cell types confirms neuroinflammation as a key shared feature of early pathogenesis in neurodegenerative conditions.
2. * Protein Quality Control and Stress Response*:
  Astrocytes: DNAJB6, HSP90AA1, DNAJB1, DDIT3.
  Neurons: HSPH1.
  These genes are involved in protein folding, heat hock and stress response. They play a critical role in the response to cellular stresses and in maintaining protein homeostasis, which is important in neurodegenerative diseases characterized by protein aggregation [34].
3. * Neuronal and Synaptic Function:*
  Neurons: *ELAVL4, RBFOX3 (NeuN), CNTNAP2, LRRC4B, SCG5, NEFM, LY6H.*
  Astrocytes: *EFNB2* (involved in neuron-glia communication).
  These genes are implicated in various aspects of neuronal function, including synaptic plasticity and neurotransmission. Their dysregulation could contribute to synaptic dysfunction previously observed in both AD and PD [35].
4. *Cell Cycle Regulation and Apoptosis:*
  Astrocytes: *GADD45A*,* ID3*,* ID2*,* CXXC5*.
  Neurons: *BRD2*,* PLK2*.
  These genes are involved in cell cycle regulation and apoptosis. Aberrant neural cell cycle regulation and neuronal death are features of both AD and PD.
5. * Metabolism and Homeostasis:*
  Astrocytes: *SLC16A1*,* FDFT1*,* HMGCS1*,* FDPS*,* SCD*,* FGFBP3*.
  Neurons: *PPA2*,* MT-ND2*.
  Metabolic dysregulation is increasingly recognized as a feature of neurodegenerative diseases. These genes are members of multiple metabolic pathways and processes, including lipid metabolism (*SCD*,* FGFBP3*) and mitochondrial pathways (*MT-ND2*, *SLC16A1*, *PPA2*, *FDPS*), which have previously been implicated in AD and PD [36].
6. *Neurodevelopment and Plasticity:*
  Astrocytes: *NNAT*,* PEG10*.
  Neurons: *NNAT*,* LMO4*.
  While primarily known for their roles in neurodevelopment, these genes may have ongoing functions in adult neuroplasticity, which could be relevant to neurodegenerative processes [37].
7. *RNA Processing and Regulation:*
  Neurons: *ELAVL4*,* RBFOX3*.
  These genes are members of RNA processing and regulation processes, which are increasingly recognized as important in neurodegenerative diseases [38].
8. *Ribosomal Function:*
  Neurons: *RPS4Y1*,* RPS29*,* EIF1AX*.
  These genes are involved in ribosomal function and protein synthesis. Significant alterations in protein synthesis have been identified in both AD and PD in multiple studies [39].

Other DEGs encode proteins involved in calcium signaling and homeostasis (*S100A6* [40]) that may reflect calcium dysregulation as a common feature of AD and PD, and genes associated with polyamine metabolism (*SAT1* [41]).

Overall, both AD- and PD-like brain organoids showed significant changes in multiple functional gene groups. They primarily involved mechanisms well-established in neurodegenerative diseases, such as synaptic dysfunction and protein misfolding, and with shared directionality of changes for the genes involved, such as *ELAVL4* and *DNAJB6* (see Table 1, upper part). However, we also identified examples of functionally related groups of DEG with contrasting direction of the change (see Table 1, lower part), e.g., the genes *ID2* and *ID3* involved in apoptosis, or the genes *SCD* and *FGFBP3* associated with lipid metabolism.

### Pathway analysis

Pathway enrichment analysis revealed distinct patterns of biological process alterations in the two organoid datasets, with both contrasting and common alterations observed in astrocytes and neurons (Table 2). In the main text, we focus on the GO database, highlighting the top five enriched pathways for both the contrasting and shared DEG categories. The complete enrichment results for these categorized DEGs, across all four databases, are provided in Tables S13 and S14.

**Table 2:**
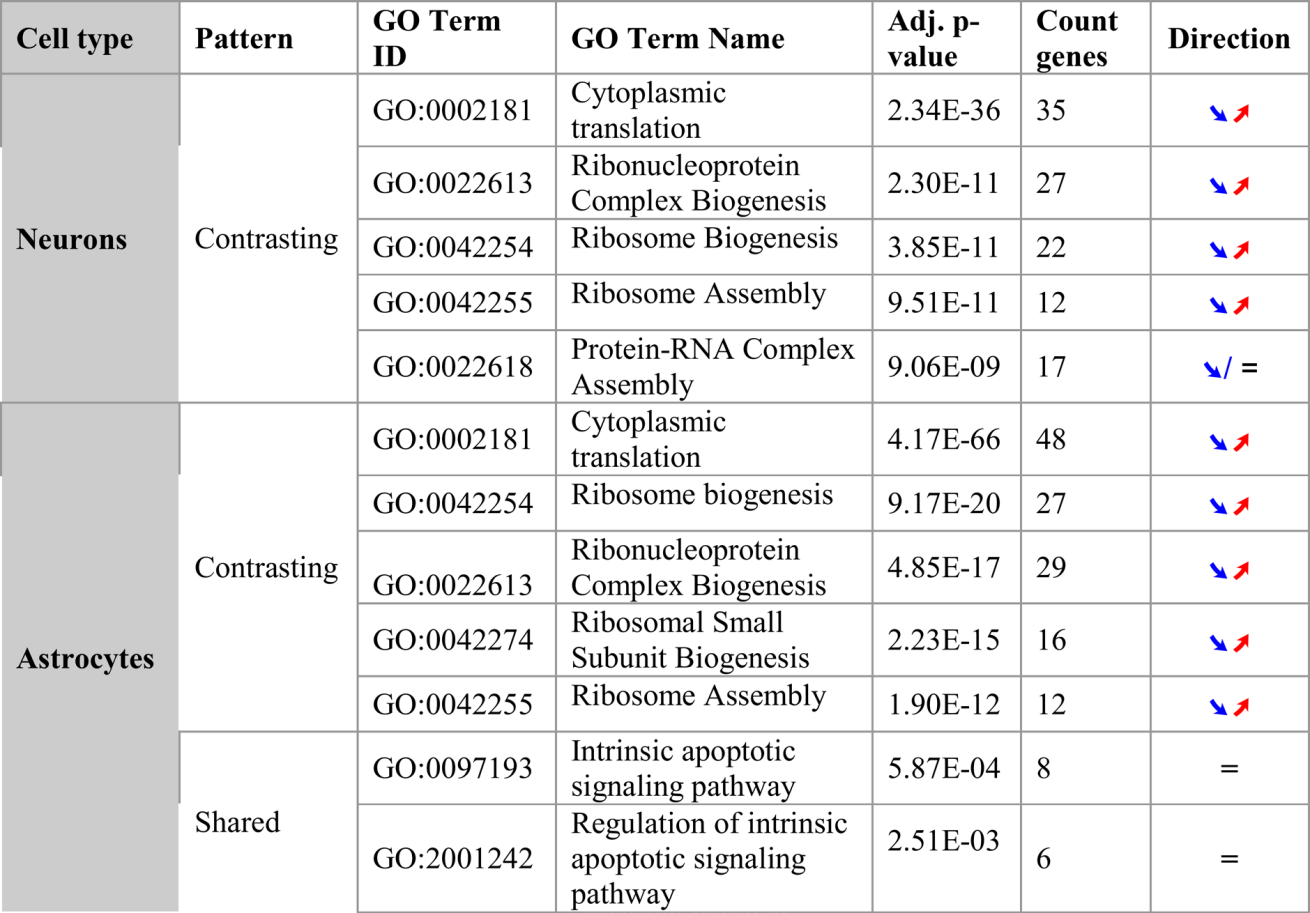
Biological processes with shared or contrasting alterations between AD-like and PD-like datasets

Gene ontology (GO) enrichment analysis of differentially expressed genes (DEGs) showing the most significant biological processes altered in both diseases.* Contrasting patterns* represent pathways where genes show opposite expression trends between the two models (e.g., upregulated in AD-like samples but downregulated in PD-like samples). *Shared patterns* represent pathways with consistent alterations across both diseases. The “Count genes” column indicates the number of DEGs mapped to each pathway. *Direction symbols*: for contrasting DEGs, arrows show expression direction in PD-like (left arrow) and AD-like conditions (right arrow), where ➘ = decreased expression and ➚ = increased expression. for shared DEGs, “=” indicates mixed or minimal average expression changes across overlapping genes (below 0.1 log2FC threshold). Analysis restricted to pathways with FDR < 0.05 and minimum 5 mapped genes. Results demonstrate opposing alterations in ribosome-related pathways and shared dysregulation of apoptotic signaling between the two organoid models

As complementary information, we also report pathway enrichment results from analyses of all significant DEGs in neurons and astrocytes. Specifically, the top 100 enriched pathways per databases are presented for the AD dataset (Tables S5, S7, S9 and S11) and the PD dataset (Tables S6, S8, S12). The full pathway ranking tables for all analyses are available in the GitLab repository associated with this study (see ‘Availability of Data and Materials’).

#### Contrasting pathways

Our analysis revealed significant divergences in ribosome-related pathways between AD-like and PD-like datasets in both astrocytes and neurons.

In astrocytes, several ribosome-associated processes showed opposing trends:

- *Ribosome biogenesis* (GO:0042254, q-value: 1.44E-19).
- *Ribosomal small subunit biogenesis* (GO:0042274, q-value: 9.68E-17).
- *Ribosome assembly* (GO:0042255, q-value: 3.37E-12).

These pathways exhibited consistent increased expression in the AD-like dataset and decreased expression in the PD-like data (see Table 2, lower part). This differential activity suggests distinct perturbations of the protein synthesis machinery in these neurodegenerative diseases. The increased expression trend in AD-like conditions may indicate a compensatory response to increased protein misfolding and aggregation, while the decreased gene expression in PD-like samples could reflect a direct impairment of ribosomal function.

Notably, a similar contrasting pattern in ribosome-related pathways was observed in neurons (see Table 2, upper part), further emphasizing the differential impact of the modeled pathologies on protein synthesis across different cell types in the central nervous system.

These findings align with and extend recent studies highlighting ribosome dysfunction as a key feature in neurodegenerative pathology [42]. The observation of these contrasting patterns in both astrocytes and neurons underscores the robustness of ribosomal pathway dysregulation in neurodegenerative and neuroinflammatory conditions.

#### Shared pathways

Our analysis revealed several common pathways significantly altered in both datasets, with shared directionality of change.

In astrocytes, multiple apoptosis-related pathways were identified as significantly enriched in both models (see Table 2, lower part). These include the pathways:

- *intrinsic apoptotic signaling* (GO:0097193, q-value: 4.87E-04),
- *regulation of intrinsic apoptotic signaling* (GO:2001242, q-value: 2.08E-03).

The shared trend of altered expression levels for *intrinsic apoptotic signaling* suggests that increased susceptibility to programmed cell death or dysregulation of cellular homeostasis mechanisms may be a common feature in astrocytes in both models. This aligns with growing evidence linking deregulation of apoptosis to neurodegenerative processes [43, 44].

Beyond the Gene Ontology analysis, pathway enrichment using additional databases (KEGG, Reactome, and WikiPathways) provided complementary insights into disease-specific molecular alterations (Supplementary Tables S5-S10, Supplementary Fig. S10). KEGG pathway analysis revealed significant enrichment of translation initiation pathways in both models, with particularly strong signals in astrocytes (AD-like data: FDR = 7.10E-93, PD-like data: FDR = 3.88E-33) and neurons (AD-like data: FDR = 4.05E-59, PD-like data: FDR = 8.69E-19), consistent with our GO findings of ribosome-related pathway alterations. Model-specific differences were evident in metabolic pathways, with AD-like data showing enrichment in mevalonate and cholesterol biosynthesis pathways, while the PD-like data demonstrated stronger associations with mitochondrial electron transport complexes and proteasome-mediated protein degradation pathways.

Reactome analysis provided broad pathway coverage, identifying 61 significant pathways in astrocytes and 96 in neurons in the AD-like data, and 237 in astrocytes and 290 in neurons in the PD-like data, with extensive overlap in translation-related processes (see statistics on the numbers of significant enriched pathways in Supplementary Fig. S10a). WikiPathways analysis, while yielding fewer significant pathways overall, confirmed the ribosomal protein and cholesterol metabolism patterns observed in other databases. The cross-disease analysis revealed that contrasting DEGs consistently showed more extensive pathway enrichment across all databases compared to shared DEGs (Supplementary Fig. S10b), supporting our conclusion that disease-specific mechanisms may be more prominent than shared pathological processes in these early-stage organoid models.

Overall, these results provide first insights into the common and diverging pathway activity alterations in models of neurodegeneration and neuroinflammation in neurons and astrocytes. They may also serve as initial pointers to determine candidate cellular processes for cross-disease or disease-specific therapeutic target discovery. In particular, the contrasting patterns in ribosomal pathways indicate that targeting disease-associated alterations in the protein synthesis machinery may require disease-specific approaches. Conversely, the common alterations of apoptotic and stress response pathways in astrocytes suggest a potential for exploring pharmaceutical intervention strategies addressing pathological changes in these specific pathways across different degenerative or neuroinflammatory conditions.

### Network analysis

A gene regulatory network (GRN) analysis was conducted to identify central regulatory genes whose modulation has the potential to reverse pathological expression changes observed downstream in the network (also called “perturbagens”, see Methods). This analysis was performed separately for shared and contrasting DEGs in astrocytes and neurons.

#### Contrasting DEGs in neurons

**Fig. 3:**
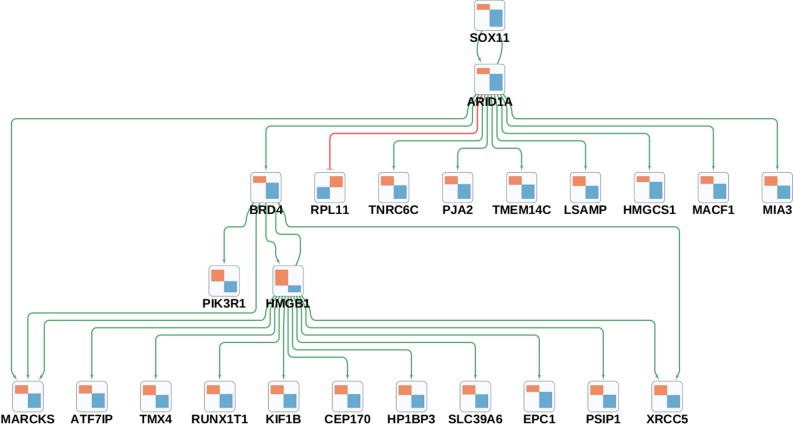
Gene regulatory subnetwork highlighting contrasting expression changes between AD-like and PD-like samples in neurons. Each box represents a differentially expressed gene (DEG), arrows for the activating interactions are highlighted in green, inhibiting interactions in red. The colored bars in the nodes represent the condition-specific gene expression changes, left for PD-like and right for AD-like samples; increases are highlighted in orange and decreases in blue

GRN analysis of contrasting DEGs in neurons revealed three major perturbagens in the network: *HMGB1*, *BRD4*, and *SOX11* (see Fig. 3).

*HMGB1* (High Mobility Group Box 1) emerged as the top upstream regulator with a perturbation score of 28, indicating its potential to modulate the expression of 28 other genes in the network (see Fig. 3). This gene encodes a multifunctional redox sensitive protein that has been implicated in neuroinflammation due to its ability to bind to inflammatory mediators (e.g., TLR4, RAGE) and to activate a pro-inflammatory signaling cascade [45, 46], which is a hallmark of multiple neurodegenerative conditions, including AD and PD [47].

*BRD4* (Bromodomain Containing 4) was identified as the second most influential perturbagen (score: 24). This gene encodes a chromatin reader protein that binds acetylated histones and is involved in epigenetic processes associated with cellular senescence and apoptosis in age-related diseases [48, 49].

The last perturbagen, *SOX11* (SRY box transcription factor 11, score: 23), is a transcriptional activator with functional roles in the regulation of adult neurogenesis and neuronal development [50, 51]. *SOX11* ablation has previously been shown to lead to reduced hippocampal neurogenesis in mice [51], explaining its potential involvement in neurodegenerative disorders.

Overall, the roles of *HMGB1*, *BRD4* and *SOX11* as key regulators in the GRN for contrasting DEGs suggests these genes may be key drivers of associated differential alterations in neuroinflammatory, epigenetic and adult neurogenesis-related processes in neurodegenerative diseases.

#### Shared DEGs in neurons

In the GRN analysis for shared DEGs in neurons, no perturbagen could be identified, probably due to the limited connectivity of only 29 edges for 30 genes in this network. However, we identified the transcription factor *JUN* as a hub node in this network with a betweenness centrality score of 0.85 and shared interactions with 14 other nodes. *JUN* is a key regulator of gene expression in response to oxidative stress which induces both antioxidant and proinflammatory pathways [52], and may thus serve as a mediator of disease response processes in the GRN for the shared DEGs in neurons.

#### Contrasting DEGs in astrocytes

In the GRN for contrasting DEGs in astrocytes, two regulatory genes were identified as key perturbagens: *CTNNB1* and *MEIS2* (both with a score of 26, see Supplementary Fig. 3).

*CTNNB1*, which encodes β-catenin, is a key mediator in the canonical Wnt signaling cascade, a pathway that promotes neuronal survival and maintenance [53]. Dysregulation of Wnt signaling has been associated with synaptic dysfunction in AD [54] and proposed as a target for neuroprotective and regenerative strategies in PD [54, 55].

*MEIS2* (Meis homeobox 2) is a transcriptional regulator, which acts as a co-factor in generic neurogenesis and dopaminergic fate specification [56]. Because *MEIS2* also increases expression of *BACE1*, a key enzyme in the amyloidogenic processing of APP, it has been suggested as a potential drug target for AD [56, 57].

Overall, the changes observed in these perturbagens and their downstream targets point to condition-specific changes in neuronal survival and neurogenesis-related pathways in the two organoid models.

#### Shared DEGs in astrocytes

**Fig. 4:**
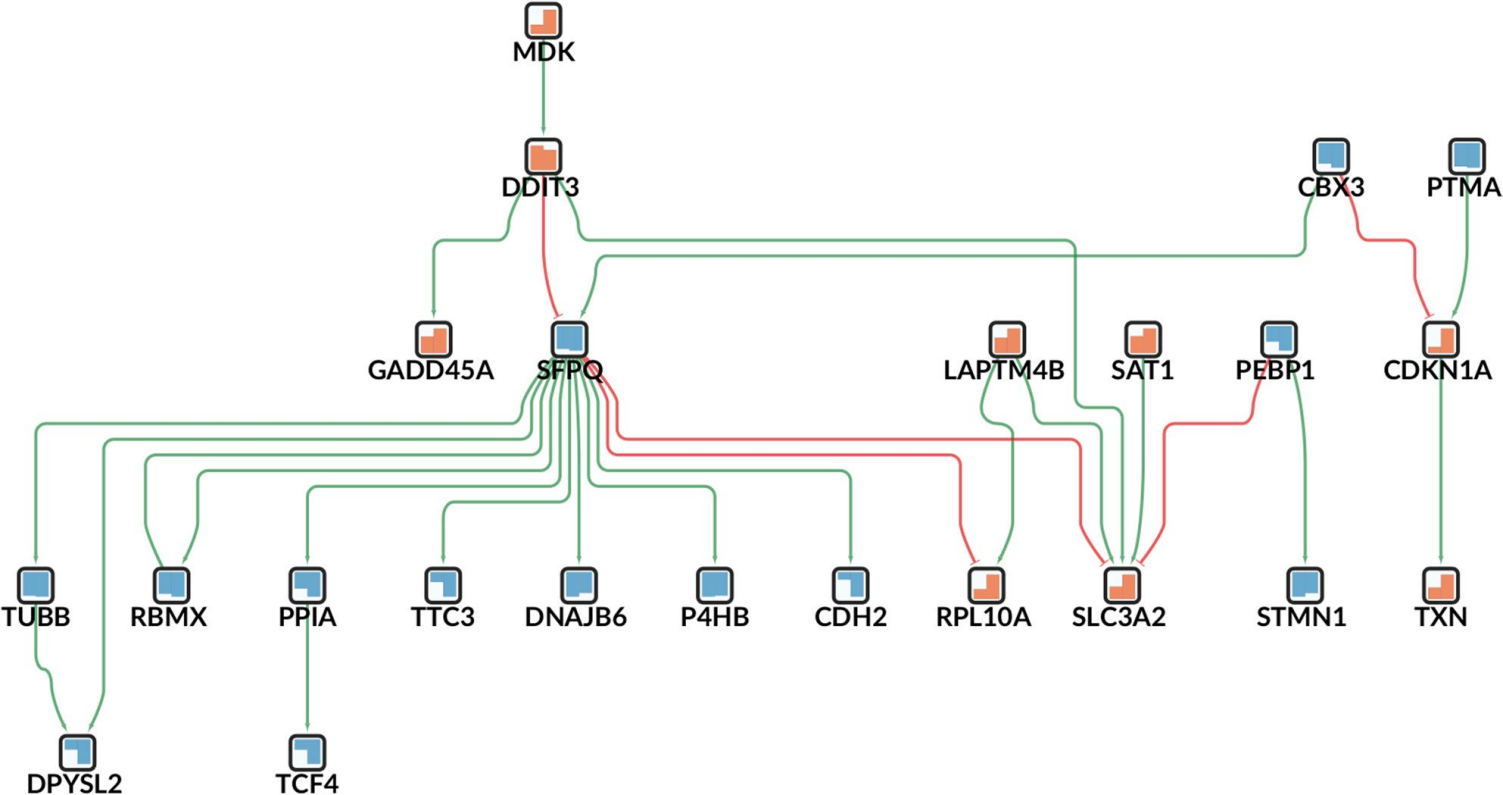
Gene regulatory subnetwork highlighting shared expression changes between AD-like and PD-like samples in astrocytes. Each box represents a differentially expressed gene (DEG), arrows for the activating interactions are highlighted in green, inhibiting interactions in red. The colored bars in the nodes represent the condition-specific gene expression changes, left for PD-like and right for AD-like samples; increases are highlighted in orange and decreases in blue.

The GRN analysis of common DEGs in astrocytes identified two perturbagens: *SFPQ* and *RBMX* (both with a score of 14, Fig. 4).

*SFPQ* (splicing factor proline and glutamine rich) is an RNA-binding protein (RBP) acting as a pre-mRNA splicing factor in spliceosome formation. Interestingly, SFPQ inclusions have been reported to accumulate in the nuclei of PD patient neurons and to sequester edited RNAs encoding proteins in axons, synapses, and mitochondria [58]. Moreover, an observed association between *SFPQ* and tau-oligomers in brains from patients with rapidly progressing AD has led to the proposition that *SFPQ* may be involved in the oligomerization and subsequent misfolding of the tau protein in AD [59].

*RBMX* (RNA Binding Motif Protein X-Linked) has been identified as a key splicing regulator in PD, inducing alternative splicing of alpha-synuclein, the protein that forms toxic aggregates in neurons as a hallmark of PD [60, 61] .

Taken together, the key roles of these RBPs as perturbagens in the shared DEG network confirms the emerging role of RNA processing dysfunction in neurodegenerative and neuroinflammatory conditions. Notably, the network analysis also revealed the role of the gene *MDK* (midkine) as an important upstream regulator in the GRN of shared DEGs in astrocytes (Fig. 4). *MDK* indirectly influences the expression of many downstream DEGs through the intermediate regulator *DDIT3*. Given that *MDK* itself was also identified as a top DEG and the associated MDK pathway was significant in the cell-cell communication analysis for both datasets (see below), it may merit further study as a potential shared disease gene.

### Cell-cell communication analysis

Our cell-cell communication analysis (see Methods) revealed a substantial number of significantly altered pathways in both astrocytes and neurons across the two organoid models. Quantitative analysis identified 56–70 significantly predicted ligand-receptor pairs per condition, with AD-like data showing higher communication complexity (66–70 pairs) compared to PD-like data (56–58 pairs). Interaction strengths varied between conditions, with astrocytes demonstrating the highest median interaction weights (0.75) and neurons showing superior ligand activity scores in the AD-like data (mean AUPR: 0.47), indicating stronger predictive capacity for explaining observed gene expression changes (Supplementary Table S15). After computing the shared significant pathways between the two organoid models, we restricted our discussion to the top 5 shared significant pathways (however, the complete lists of significant shared and non-shared pathways are provided in the GitLab repository associated with the manuscript, see ‘Availability of Data and Materials’).

#### Results for neurons

The analysis of neuronal pathways yielded approximately 864 significant Gene Ontology (GO) terms for the AD-like data, and 557 significant pathways for the PD-like data, highlighting the complex cellular communication landscape in neurodegenerative diseases. By intersecting these significant pathways, we identified 388 pathways that are significantly altered in neurons of both organoid models. These shared pathways suggest common underlying mechanisms in neuronal dysfunction across the modeled neurodegenerative and neuroinflammatory conditions. Among the most significant shared pathways, we identified the following five key processes.

1. Axonogenesis (GO:0007409): This pathway was highly significant in both AD-like (p-adjust = 5.21e-18) and PD-like data (p-adjust = 4.24e-17). In the AD-like data, 59 genes were involved, while in the PD-like data, 47 genes were implicated. This suggests that axon development and growth are significantly affected in both diseases, which may contribute to the observed neuronal loss and synaptic dysfunction.
2. Axon development (GO:0061564): Closely related to axonogenesis, this pathway was also highly significant in both the AD-like (p-adjust = 1.15e-17) and PD-like data (p-adjust = 1.36e-19). The involvement of 62 genes in the AD-like dataset and 53 genes in the PD-like data indicates a broader impact on axonal processes, including guidance, elongation, and maintenance.
3. Cell morphogenesis involved in neuron differentiation (GO:0048667): This pathway showed significant alterations in both AD-like (p-adjust = 1.41e-17) and PD-like conditions (p-adjust = 5.19e-15), with 65 and 49 genes involved, respectively. This suggests that the overall process of neuronal differentiation and morphological development is disrupted in both diseases.
4. Neuron projection morphogenesis (GO:0048812): Similarly, this pathway was significantly altered in both AD-like (p-adjust = 1.41e-17) and PD-like data (p-adjust = 1.61e-14), involving 69 and 51 genes, respectively. This indicates that the formation and remodeling of neuronal projections, including dendrites and axons, are affected in both diseases.
5. Plasma membrane bounded cell projection morphogenesis (GO:0120039): This broader category, which encompasses various cellular protrusions, was also significantly altered in both AD-like (p-adjust = 1.41e-17) and PD-like samples (p-adjust = 3.95e-14), with 70 and 51 genes involved, respectively.

These shared pathways highlight the importance of neuronal structural integrity and plasticity in both organoid models. The disruption of axon development, neuronal differentiation, and projection morphogenesis suggests that both diseases may share common mechanisms leading to neuronal dysfunction and eventual neurodegeneration.

#### Results for astrocytes

Similar to neurons, our investigation into cell-cell communication in astrocytes revealed many significant GO terms shared between the two models, pointing to common mechanisms of astrocyte dysfunction that may contribute to the progression of neurodegenerative and neuroinflammatory conditions. Among the key shared pathways, we identified.

1. Tube morphogenesis (GO:0035239): This pathway emerged as highly significant in both AD-like (p-adjust = 6.66e-25) and PD-like data (p-adjust = 1.18e-18), involving 67 and 63 genes, respectively. The high significance of this pathway suggests that astrocytes may play a previously underappreciated role in shaping brain microarchitecture, particularly in the context of the neurovascular unit and blood-brain barrier integrity.
2. Cell-cell adhesion (GO:0098609): With 70 genes implicated in AD-like (p-adjust = 2.12e-23) and 74 in PD-like conditions (p-adjust = 1.17e-20), this pathway highlights the important role of astrocytes in maintaining cellular cohesion within the brain. Disruption of these adhesion processes could potentially compromise the structural integrity of the neurovascular unit and alter glial-neuronal interactions.
3. Blood vessel development (GO:0001568): The significant alteration of this pathway in both AD-like (p-adjust = 2.23e-22, 57 genes) and PD-like samples (p-adjust = 5.72e-16, 52 genes) points to a possible role of astrocytes in the vascular changes observed in these diseases. This finding may have implications for brain perfusion and the delivery of essential nutrients and oxygen to neurons.
4. Vasculature development (GO:0001944): Closely related to blood vessel development, this pathway showed similar significance in AD-like (p-adjust = 3.62e-22, 58 genes) and PD-like data (p-adjust = 2.78e-16, 54 genes). The involvement of astrocytes in vascular remodeling could represent a key point of intervention in neurodegenerative disease progression.
5. Axonogenesis (GO:0007409): It is important to interpret the significant alteration of this pathway in astrocytes in both AD-like (p-adjust = 1.52e-21, 53 genes) and PD-like samples (p-adjust = 1.59e-18, 55 genes) within the context of the cell-cell communication framework. This finding does not imply that astrocytes themselves are undergoing axonogenesis. Instead, it suggests a significant disruption in the signaling dialogue between astrocytes and neurons, indicating that altered ligand-receptor interactions involving astrocytes are drivers of expression changes in axon-related genes within their target cells, primarily neurons.

These shared pathways indicate the multifaceted role of astrocytes in the pathogenesis of neurodegenerative and neuroinflammatory conditions, particularly in relation to vascular function, cellular adhesion, and, intriguingly, potentially also in axonal development. The alterations of these pathways suggest that astrocyte dysfunction may be a central player in the progression of both diseases through multiple mechanisms. Regarding the differences observed between the two organoid datasets in this analysis, we note that they may be influenced by technical and biological differences unrelated to disease, including the differences in organoid maturation time and experimental protocols described in our quality control analysis. While these results offer first insights and testable hypotheses on potential disease-related pathways, both shared and disease-specific findings require independent validation in future studies with controlled experimental conditions.

### Comparison with post-mortem brain tissue studies

To obtain complementary information on the disease-relevance of our findings from the brain organoid models, we performed two complementary overlap analyses between organoid and post-mortem tissue datasets (The full list of differentially expressed genes (DEGs) from *post-mortem* (PMT) and organoid datasets is provided as supplementary tables ST19-28).

1. *Condition-Specific Overlap Analysis*: For each modelled disease condition and cell type combination, we calculated the proportion of organoid DEGs that were also significantly altered in the corresponding *post-mortem* analysis (see Methods). Overlaps were computed both with and without taking the directionality of expression changes into account (Figure S9).

For the AD-like dataset, we identified 418 significant DEGs in organoid astrocytes, of which 206 genes (49.3%) were also significantly altered in *post-mortem* astrocytes. In organoid neurons, 1,061 DEGs were identified, with 329 genes (31.0%) overlapping with *post-mortem* neuronal signatures. For the PD-like data, organoid astrocytes showed 872 significant DEGs, with 193 genes (22.1%) also present in *post-mortem* astrocyte signatures. Organoid neurons displayed 1,033 DEGs, of which 397 genes (38.4%) overlapped with *post-mortem* neuronal changes.

Fisher’s exact test analysis revealed statistically significant overlaps for most of these comparisons with varying effect sizes (see Suppl. Tab. S4). Neurons in the PD-like model showed the strongest association (*p* = 2.13 × 10⁻^66^), followed by astrocytes in the AD-like model (*p* = 4.57 × 10⁻^24^) and astrocytes in the PD-like model (*p* = 6.3 × 10^− 08^). Notably, neurons in the AD-like model showed no significant association (*p* = 1.0), indicating that these neuronal DEGs were not more likely to be found among *post-mortem* DEGs than would be expected by chance, possibly reflecting the limitations of this organoid model and the substantial differences in developmental stage and cellular context between the experimental systems. Overall, these results indicate that organoid models of neurodegeneration and neuroinflammation capture a subset of the molecular signatures observed in end-stage *post-mortem* disease tissue, and vice-versa, *post-mortem* stage alterations capture significant subsets of organoid model changes, with the proportions varying by condition and cell type (detailed breakdown of the bidirectional comparisons provided in Suppl. Tab. S4). The moderate overlaps for both directional and undirectional analyses reflect the model limitations and fundamental biological differences between early developmental organoids and mature *post-mortem* tissue, while suggesting that some pathology-associated molecular signatures are detectable across these different experimental contexts.

2) *Cross-Disease Signature Overlap Analysis*: In a more stringent analysis, we examined whether genes we had identified as “shared” (dysregulated in the same direction in both organoid models) or “contrasting” (dysregulated in opposite directions between the models) were also significantly dysregulated in *post-mortem* tissue studies. As expected, this more stringent analysis revealed more modest overlap percentages, ranging from 4.2% to 8.5% across different cell types and DEG categories. Specifically, for shared DEGs (genes altered in the same direction in both organoid models):
  Astrocytes: 6.5% of organoid shared DEGs were also altered in *post-mortem* tissue (3 overlapping genes, not reaching significance: *p* = 0.188).
  Neurons: 4.2% of organoid shared DEGs were also found altered in *post-mortem* tissue (6 overlapping genes, not reaching significance: *p* = 0.28).

For contrasting DEGs (genes altered in opposite directions between the two organoid models):

- Astrocytes: 8.5% of organoid contrasting DEGs were also altered in *post-mortem* tissue (11 overlapping genes, *p* = 0.00971).
- Neurons: 4.5% of organoid contrasting DEGs were also altered in *post-mortem* tissue (8 overlapping genes, not reaching significance: *p* = 0.0646).

The lower percentages in this analysis reflect the more stringent criteria: genes must first be identified as having consistent cross-disease patterns in organoids and then also be significantly dysregulated in *post-mortem* tissue. While the goal of this analysis was not to identify global, omics-scale similarities in gene expression signatures, but rather to detect a small subset of genes with robust shared neurodegeneration- and neuroinflammation-associated patterns across different experimental settings, we also performed a significance-of-overlap analysis (see Methods). In line with our expectations, significant overrepresentations of DEGs from the organoid model analyses among the DEGs from the *post-mortem* data in this more stringent analysis were only detectable for the larger set of contrasting DEGs (astrocytes: *p* = 0.00971, neurons: *p* = 0.0646), but not for the smaller sets of shared DEGs (astrocytes *p* = 0.188, neurons: *p* = 0.28). However, some of the key genes identified in our organoid analysis fulfilled the stringent criteria of displaying shared significance and shared directionality of alteration patterns in the *post-mortem* data:

1. In astrocytes:
  - Contrasting DEGs: *FOS*,* PTPRZ1*,* MT-ND4*,* PLP1*,* ID3*,* DNAJB1*,* RTN1*,* HSP90AA1*,* HSPB1*,* DDIT4* and *GAPDH*.
  - Shared DEGs: *RPL10A*,* GADD45A* and *PPDPF*.
2. In neurons:
  - Contrasting DEGs: *RPL37A*,* MARCKS*,* GNAS*,* RPS5*,* HMGB1*,* NNAT*,* BRD4* and *ANP32A*.
  - Shared DEGs: *HINT1*,* CCDC85B*,* ASAH1*,* TMEM14A*,* HES4* and *GUK1*.

Interestingly, in multiple cases the directionality of expression changes differed between organoid and *post-mortem* tissue. For example, *ID3* showed increased expression in the AD-like data and decreased expression in the PD-like data, while displaying the opposite pattern in *post-mortem* tissue. When considering directionality in the overlap analysis, only three DEGs across all categories and cell types showed consistent directionality between the two study types: *DDIT4*, *PLP1*, and *GNAS*. These differences might reflect the distinct pathology stages being captured (early-stage in organoids versus end-stage in *post-mortem* tissue) or the inherent limitations of each model system. In particular, the interpretation of this *post-mortem* tissue comparison is limited by differences in cell type resolution and biological context between the experimental systems. The organoid datasets necessarily represent broader, less differentiated cell populations compared to the refined neuronal and glial subtypes identifiable in mature *post-mortem* brain tissue. Our comparison used aggregated *post-mortem* neuronal subtypes to match organoid resolution, but this approach may mask subtype-specific disease effects that are present in the more mature tissue context.

Furthermore, the biological contexts represented are different: early developmental organoids cultured in vitro versus fully mature brain tissue from end-stage disease patients. The modest overlap percentages we observed for the stringent analyses (0-8.5% of the shared and contrasting DEG categories across directional and undirectional analyses, Figure S9) should be interpreted considering these substantial methodological and biological differences. They should not be interpreted as a validation of organoid analysis results but viewed as a complementary finding, indicating the genes of highest robustness across disease stages and experimental approaches.

Overall, these findings highlight that brain organoids and *post-mortem* tissue capture different aspects of neurodegeneration biology, reflecting their distinct developmental stages, cellular contexts, and disease phases. This underscores the complementary rather than validatory nature of these different model systems and the importance of considering disease stage and model system limitations when interpreting molecular findings.

### GWAS overlap analysis

AD-like dataset: Studying the overlap between DEGs from the AD-like organoid model and the significant genes from the AD GWAS data revealed that 18 astrocyte DEGs and 58 neuron DEGs from the AD-like dataset corresponded to significant GWAS genes, representing 4.52% and 5.64% of the total significant DEGs for astrocytes and neurons, respectively. In total, 67 genes were shared between the AD-like dataset and the AD GWAS study (see Suppl. Table 16, containing the complete statistics and gene symbols for the overlapping genes). They include some of the most significant DEGs in our analysis, such as *EFNB2*, *ELAVL4*, and *CNTNAP2*, also listed in Table 1 among the top 10 DEGs. *EFNB2* belongs to the shared astrocyte category, while *ELAVL4* and *CNTNAP2* are found in the shared and contrasting neuron categories, respectively. Additionally, *MARCKS*, identified as a contrasting neuronal DEG, overlaps with both *post-mortem* study DEGs and the AD GWAS gene list (see Supplementary Table S17). *MEIS2*, highlighted as a key regulator in the network analysis of contrasting astrocyte DEGs, is also present in the AD GWAS list. Finally, *SOX11*, which appears in the AD GWAS dataset and is highlighted in the network analysis, is identified as a DEG with a consistent direction of change in both the Alzheimer’s *post-mortem* study and the organoid study.

PD-like dataset: The overlap between the DEGs from the PD brain organoid dataset and the PD GWAS-derived significant genes was more limited, with only 9 intersecting genes (*RPS12*, *CTSB*, *PTPRN2*, *TOX3*, *PDCD5*, *FYN*, *ZYG11B*, *NCOR1*, and *PTPN1*), covering 0.37 and 0.73% of DEGs for astrocytes and neurons, respectively. This more restricted overlap likely reflects the smaller number of significant genes for the PD GWAS data (132 genes) compared with the AD GWAS data (758 genes).

While Fisher’s exact test did not reveal statistical significance for the assessed overlaps with the GWAS-derived genes (p-values ranging from 0.15 to 0.97 depending on the considered combination of disease and cell type, see Suppl. Tab. S16), this is consistent with the expected differences between genomic variation (associated with disease susceptibility) and transcriptomic alterations (reflecting disease-associated cellular responses). Importantly, the identified overlapping genes, including several from our top-ranked signatures such as *EFNB2*, *ELAVL4*, *CNTNAP2*, and *MEIS2*, represent a refined subset of high-confidence candidate disease genes that integrate both genetic risk and functional expression changes, making them particularly valuable for prioritizing follow-up functional validation studies.

Additionally, all organoid DEGs that are also identified in both post-mortem studies and GWAS datasets are listed in Tables S17 (without considering the consistency of the change direction of the DEG between *post-mortem* and organoid studies) and S18 (taking the directionality of the change into account).

## Limitations

While our analysis benefits from the fact that each individual dataset was processed as a single experimental batch, eliminating concerns about intra-dataset batch effects, important limitations should be considered when interpreting the results.

First, the substantial difference in organoid culture duration between the AD-like (94–95 days) and PD-like (30 days) datasets represents a significant methodological limitation that could confound our cross-disease comparisons. Brain organoids undergo extensive transcriptional and cellular remodeling during maturation, and this ~ 65-day difference likely represents different developmental stages that could overshadow genuine disease-specific signatures. For this reason, we have checked alterations in developmental and maturation marker genes and pathways and confirmed that our disease-associated signatures show minimal overlap with developmental pathways (see Suppl. Fig. S7 and S8 and the pathway rankings in Suppl. Tab. S5 to S12). While our within-dataset analytical approach (comparing disease vs. control within each study) is matched for developmental stage in the primary differential expression analysis, the subsequent cross-disease comparisons of gene signatures may however still be influenced by maturation state differences. In the DEG ranking tables for the organoid models, we have therefore marked all genes that are implicated in developmental processes with an asterisk (*), as their differences across datasets may at least partly reflect developmental stage differences. This limitation particularly affects the interpretation of our contrasting gene signatures and requires validation in studies using temporally matched organoid cultures. However, the comparison of key findings with *post-mortem* human tissue provides important complementary evidence of disease relevance despite these temporal differences.

Second, the brain organoids used in this study represent models of early-stage pathology processes and may not fully capture the complexity of neurodegeneration in the human brain. Additionally, the AD-like pathology model presents specific limitations that distinguish it from the genetic PD model. While the serum treatment approach successfully induces a subset of key features of AD pathology including amyloid-β aggregation, tau phosphorylation, synaptic loss, and neuroinflammation as demonstrated in the original Chen et al. study, it lacks the established AD-specificity of genetic models involving familial AD mutations. The serum treatment represents a more generic approach to inducing neurodegeneration that primarily captures blood-brain barrier dysfunction and its downstream consequences in AD, rather than the specific genetic mechanisms underlying familial forms of the disease. This distinction is important when interpreting comparative results, as the AD-like model represents environmentally-induced pathology while the PD model captures genetically-driven mechanisms.

In addition, the organoids lack certain features of the mature human brain, including a fully developed vasculature, immune cells, and the blood-brain barrier. This limitation restricts our ability to study complex interactions among cell types and systems present in the living brain.

Third, our study focused primarily on neurons and astrocytes due to their representation in both datasets. However, other cell types important in neurodegeneration, such as microglia and oligodendrocytes, could therefore not be analyzed. This means we may have missed a subset of relevant changes specific to these other cell types and intercellular interactions involved in disease progression.

Fourth, the available sample size limits the statistical power and may have prevented us from detecting additional molecular changes that are more subtle. The statistical results for our differential expression analyses should be interpreted with this limitation in mind.

Fifth, while comparison with *post-mortem* data provided insights into the robustness of our findings, the fundamental biological and methodological differences between organoid and *post-mortem* systems present major interpretive challenges. These systems represent significantly different biological contexts: early developmental organoids cultured in vitro versus mature brain tissue from end-stage disease patients with decades of pathological progression. Additionally, significant differences in cell type resolution exist, with *post-mortem* analyses capturing refined neuronal subtypes and mature glial populations that are not adequately represented in the broader cell clusters identifiable in organoid cultures. The small overlap percentages between systems, especially when using the most stringent settings accounting for directionality and cross-disease consistency, reflect these fundamental differences. The observed overlaps should not be interpreted as validation, but rather as complementary evidence that some disease-associated molecular signatures may be detectable across different experimental contexts and disease stages.

Sixth, our study is purely observational regarding potential therapeutic targets and lacks functional validation of any identified candidates. While we identified regulatory genes of potential interest in the network analysis, including *MDK*, determining possible beneficial or detrimental effects of modulating these genes requires extensive additional work including: (1) functional manipulation experiments in organoid models to demonstrate causality between gene expression changes and disease phenotypes, (2) rescue experiments showing that modulating target genes can impact disease-associated molecular or cellular changes, (3) validation in animal models, and (4) assessment of the complex, potentially contradictory roles these genes may play in different disease contexts or stages. Although previous literature has suggested *MDK* as a candidate therapeutic target in neurodegenerative disease contexts [62–64], our specific findings require disease-specific validation. Transcriptomic changes alone are insufficient for therapeutic target validation, and our findings should be interpreted as generating hypotheses for future functional studies rather than providing ready-to-test therapeutic strategies.

Finally, as this is the first comparative analysis of its kind using brain organoid models, replication studies will be essential to confirm our findings. The identified molecular signatures should be validated in further independent datasets and different experimental models.

## Discussion & conclusion

**Fig. 5:**
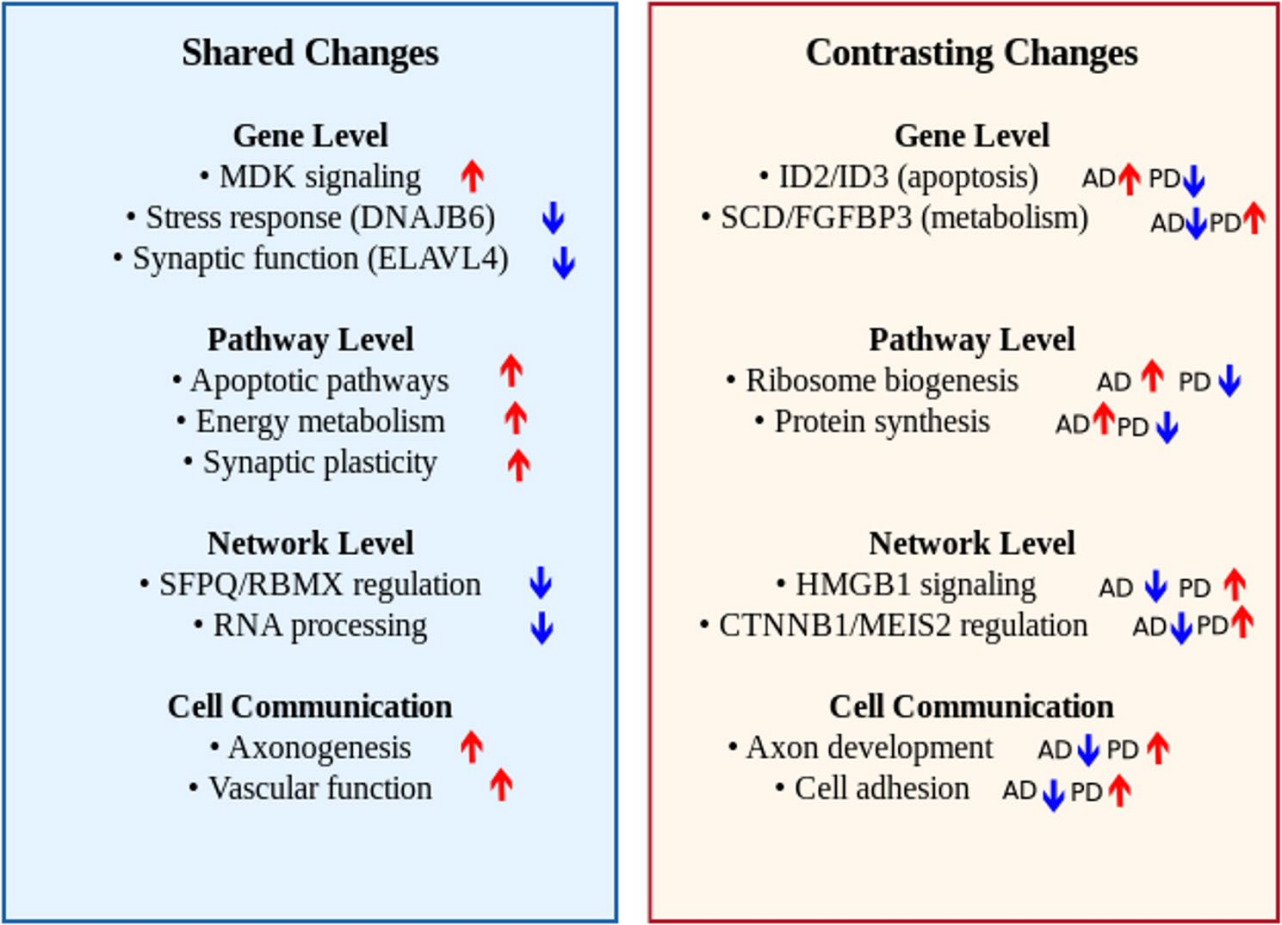
Summary of key pathways and genes altered in the AD- and PD-like datasets across multiple analyses. The most pronounced and consistent shared (left side) and contrasting (right side) changes between the AD- and PD-like datasets are highlighted, covering significant alterations detected in gene and pathway analyses, cell-cell communication, and network analyses. The arrows indicate whether the associated pathway member genes show increased (⬆) or decreased (⬇) mean expression trends. For the contrasting changes, two arrows indicate the condition-specific changes in AD-like (AD) or PD-like (PD) models

This study aimed to elucidate common and divergent cellular process alterations in individual brain cell types using brain organoid models of neurodegeneration and neuroinflammation. By comparing single-cell transcriptomic data from two organoid models, we sought to identify shared pathways and potential cross-disease therapeutic targets, as well as disease-specific alterations that could inform tailored treatment strategies. Our analysis, conducted at multiple biological levels, revealed the following main findings (see also the short summary of key findings presented in Fig. 5):

1. *Gene-level analysis*: We identified both shared and contrasting differentially expressed genes (DEGs) in neurons and astrocytes between the two organoid models. Key shared DEGs include genes involved in protein quality control and stress response (e.g., *DNAJB6*, *HSP90AA1*), neuronal and synaptic function (e.g., *ELAVL4*, *RBFOX3*), and mitochondrial pathways (e.g., *MT-ND2*, *SLC16A1*) and lipid metabolism (e.g., *SLC16A1*). Notably, *MDK* (midkine) emerged as a significant shared DEG and central regulator in the network analysis. Previous studies have implicated *MDK* in mediating immune cell recruitment and modulating chemokine expression [62, 65], suggesting potential roles in the neuroinflammatory responses observed in our disease models. However, it is important to note that *MDK*’s role in neurodegeneration appears to be context-dependent, with the literature revealing dual and sometimes opposing functions that vary according to disease type, progression stage, and cellular environment. While some studies suggest neuroprotective functions [66], others indicate pro-inflammatory or potentially detrimental effects [67, 68]. Our transcriptomic analysis alone cannot determine whether *MDK* upregulation represents a protective response or contributes to pathological processes. Furthermore, *MDK* was not among the genes showing consistent directionality between our organoid and *post-mortem* datasets, highlighting the uncertainty surrounding its functional significance in human disease contexts. Elucidating *MDK*’s potential role in neurodegeneration and neuroinflammation will therefore require follow-up functional validation studies.
2. *Pathway-level analysis*: (a) Contrasting alterations: We observed opposing trends in ribosome-related pathways between the two organoid models in both astrocytes and neurons. This suggests that while protein synthesis machinery is affected in both diseases, the underlying mechanisms may be distinct. These findings align with emerging evidence of ribosome dysfunction in neurodegeneration and highlight the potential of targeting ribosome biogenesis as a therapeutic strategy, albeit with disease-specific approaches. (b) Shared alterations: Common dysregulation of intrinsic apoptotic pathways was identified in astrocytes for both organoid models. This corroborates the well-established link between neuronal loss and apoptosis in neurodegenerative diseases [69]. In neurons, we observed contrasting changes in ribosome-related pathways, suggesting different mechanisms in response to neurodegenerative processes.
3. *Network and cell-cell communication analysis*: (a) Network analysis: Key regulatory genes modulating the observed downstream disease-associated DEGs were identified, including *HMGB1*, *CTNNB1*, and *MEIS2* for contrasting DEGs, and *SFPQ* and *RBMX* for shared DEGs. *HMGB1* emerged as a potential indicator of altered neuroinflammation, while SFPQ dysregulation appears to reflect multiple features of neurodegeneration, including aberrant RNA splicing and protein aggregation. *SOX11*, identified as a key regulator of contrasting neuronal DEGs, is also present in both the AD *post-mortem* study and the AD GWAS dataset. (b) Cell-cell communication: We identified common perturbations affecting several signaling pathways. Specifically, in neurons from both models, we identified altered pathways related to neuronal function, and in astrocytes, we identified shared altered pathways associated with vascular function, cell adhesion, and axon development.

The identification of contrasting and cell type-specific alterations in neuroinflammatory signaling pathways in both organoid models, such as those associated with *HMGB1* and *MDK*, highlights the complex nature of neuroinflammation in these diseases. The literature presents conflicting evidence regarding *MDK*’s role in neurodegeneration. Some studies suggest neuroprotective functions, e.g., *MDK* has been shown to promote neuronal survival and neurite outgrowth in vitro [70–72], and *MDK* knockout mice exhibit increased susceptibility to ischemic brain injury [67]. Additionally, *MDK* can promote oligodendrocyte differentiation and remyelination, potentially beneficial in neurodegenerative contexts [62]. Studies in animal models demonstrate that injection of MDK-encoding adenovirus after ischemic injury decreases infarct volume and protects against ischemic damage, while intrathecal administration of *MDK* promotes functional recovery upon spinal cord injury in rats [73–75]. However, other evidence suggests potentially detrimental roles. *MDK* can promote microglial activation and neuroinflammation [67, 76], and elevated *MDK* levels have been associated with increased inflammatory responses in various CNS pathologies [62]. Furthermore, *MDK*’s role in promoting angiogenesis and cell migration could potentially contribute to pathological processes in certain disease contexts [77, 78]. This contradictory evidence illustrates an important limitation of our study: transcriptomic upregulation of *MDK* in our disease models could represent a protective response to cellular stress or a contributor to pathological processes. Therefore, functional validation experiments will be needed to demonstrate the consequences of *MDK* modulation in the organoid systems.

### Comparison with post-mortem brain tissue and GWAS data

To address potential concerns about Type I errors inherent in single-cell differential expression analysis, we used a multi-level biological evaluation and filtering strategy that prioritizes DEGs showing convergent results across multiple independent lines of evidence. This approach builds on the complementary strengths of different data modalities (organoid models for early disease mechanisms, post-mortem tissue for end-stage pathology, and GWAS for genetic risk) while accounting for the inherent limitations of each source to identify genes with the most robust and consistent evidence of disease relevance. The overlaps of our organoid findings with independent *post-mortem* tissue datasets and GWAS-derived disease genes suggest that the DEGs capture genuine disease-relevant mechanisms and provide a refined subset of high-confidence intersecting significant disease-associated genes.

However, the comparison between brain organoid and *post-mortem* tissue data reveals not only the potential but also the limitations of cross-system validation approaches in neurodegeneration research. These experimental systems are fundamentally non-equivalent: organoids represent early developmental, in vitro neuroepithelial systems lacking vasculature, immune cells, and systemic context, while *post-mortem* tissue reflects fully mature, end-stage disease brain tissue influenced by chronic pathology, systemic aging, and complex cellular interactions developed over decades. The cell type populations are also inherently different in maturity and functional specialization. Organoid neurons and astrocytes represent relatively immature cell states compared to their fully differentiated counterparts in adult brain tissue, which have undergone extensive maturation and functional specialization. Additionally, *post-mortem* tissue includes diverse neuronal subtypes and mature glial populations that are not adequately represented in organoid cultures.

Given these fundamental differences, the gene overlap we observe should not be interpreted as validation of organoid findings, but rather as a complementary finding for hypothesis generation, showing that some molecular signatures associated with neurodegeneration may be detectable across very different experimental contexts and disease stages. The overlap percentages (22–50%) from disease-specific comparisons suggest that a subset of PD-associated changes in organoids affects genes that may be of particular interest across different stages of pathology, since they also display significant PD-associated alterations in a late-stage *post-mortem* dataset. The lower percentages (4–8%) from the cross-model signature analysis reflect a more stringent assessment, focusing on genes that show consistent patterns across both organoid models and appear in *post-mortem* studies. This limited overlap observed when stringent criteria are applied likely results from the model limitations, and technical and biological differences between the organoid and *post-mortem* datasets, including differences in developmental stage, in cellular composition and maturity, in the represented pathology progression stages, temporal dynamics and technical platforms.

Similarly, our GWAS overlap analysis revealed modest but biologically informative intersections between organoid DEGs and established genetic risk loci. The overlap percentages (0.37–5.64%) reflect the expected differences between genetic susceptibility variants and functional expression changes, representing complementary rather than directly overlapping aspects of disease biology. Importantly, several key genes from our top-ranked organoid signatures, including *EFNB2*, *ELAVL4*, *CNTNAP2*, and *MEIS2*, were present in both organoid and GWAS datasets, suggesting these represent particularly robust candidate disease genes that interlink genetic risk with functional alterations. In addition, *EFNB2* and *ELAVL4* were also found in the overlap with the DEGs from the *post-mortem* data.

While these small sets of overlapping genes with consistent patterns across very diverse experimental systems may represent particularly robust markers of neuroinflammatory or neurodegenerative processes, this possible interpretation requires caution and further independent validation in longitudinal and temporally matched studies.

Taken together, these findings may contribute to a more detailed understanding of the molecular underpinnings of neurodegenerative and neuroinflammatory processes, revealing both shared and distinct pathway and network alteration mechanisms. The identification of common pathways in both model systems, such as apoptotic pathways in astrocytes, provides a foundation for the follow-up exploration of cross-disease therapeutic strategies targeting generic processes of neuroinflammation or neurodegeneration. Simultaneously, the contrasting alteration patterns observed in specific cellular processes, such as those associated with *HMGB1* and *MDK*, underscore the need for condition-specific approaches in treatment strategies focused on specific pathology-associated pathways.

However, these findings must be interpreted with consideration of the substantial difference in organoid maturation time between datasets, which represents a significant limitation requiring validation in future studies with temporally matched experimental designs. Nevertheless, our statistical framework and complementary *post-mortem* analysis provide confidence in the biological relevance of our most robust findings across different experimental settings. Future research directions could also include the confirmation of key findings in additional brain organoid datasets and other model systems to increase robustness across different experimental conditions. Moreover, studies with larger sample sizes would enable the application of mixed-effects approaches that could provide more statistically rigorous differential expression analyses while accounting for individual donor variability. Additional in-depth functional studies of the identified genes and pathways, especially *MDK*, are also needed to further elucidate their specific roles in neuroinflammation and neurodegeneration. In particular, further investigation of the contrasting alterations in ribosome biogenesis is required to understand their underlying mechanisms and implications for disease progression and treatment. Concerning the therapeutic potential of targeting common pathways, such as anti-apoptotic interventions or *MDK* modulation, this may warrant exploration in preclinical models of both diseases. Finally, integration of these findings with data from human *post-mortem* brain tissue and other neurodegenerative diseases may help to identify broader patterns of neurodegeneration, potentially helping to prepare the way for more effective cross-disease therapeutic strategies.

This study provides a first comprehensive multi-level comparison of early molecular changes in brain organoid models of neurodegeneration and neuroinflammation, revealing three key conclusions.

As a main finding, we identified the midkine (MDK) signaling pathway as robustly altered across gene-, pathway- and network-level analyses in both diseases. This finding is of particular interest given *MDK*’s established role as a key regulator of inflammatory responses, mediating immune cell recruitment and chemokine expression, while also promoting cell survival and tissue regeneration [62, 79, 80]. The dysregulation of MDK signaling could therefore represent a critical early event in both diseases, linking inflammatory responses to subsequent neurodegenerative changes. Given prior evidence showing that *MDK* modulation can reduce neuroinflammation [67], promote neuronal survival [81], and improve cognitive function in animal models [70], this signaling pathway may merit further exploration in future studies. As another key finding, we identified contrasting alterations in ribosome-related pathways between the two organoid models, suggesting that while dysfunction of the protein synthesis machinery is common to both models, the underlying mechanisms are distinct. Among the main shared alterations identified, we observed common dysregulations of apoptotic pathways in astrocytes and energy metabolism pathways in neurons, which could represent either pathological mechanisms or compensatory responses requiring further investigation to determine their therapeutic potential.

These results can provide relevant information for early preclinical research on intervention strategies in neurodegenerative diseases, e.g., by prioritizing shared pathway alterations for further functional validation. Furthermore, our results highlight the value of brain organoid models for studying early-stage molecular changes in neurodegenerative diseases, providing insights that may not be accessible through *post-mortem* tissue analysis.

Our findings lay the groundwork for future studies exploring cross-disease therapeutic strategies and underscore the importance of considering both common and distinct pathological and protective mechanisms when developing treatments for neurodegenerative diseases. The identification of specific pathways and regulatory networks that are altered in both diseases or show disease-specific changes, especially in HMGB1- and MDK-mediated signaling, provides concrete starting points for such investigations.

## Supplementary Information

Supplementary Material 1.

## Data Availability

The AD dataset is available in the NCBI Gene Expression Omnibus (GEO) database (Accession number: GSE164089). The PD dataset will become available in the NCBI GEO database (release pending). All scripts used for data analysis are available on GitLab: https://gitlab.com/uniluxembourg/lcsb/bds/brain_organoid_ad_pd_comparison.

## Abbreviations

AD: Alzheimer’s Disease
APP: Amyloid Precursor Protein
BACE1: Beta-secretase 1
BBB: Blood-Brain Barrier
BP: Biological Process
DEGs: Differentially Expressed Genes
DDIT3: DNA Damage Inducible Transcript 3
FDR: False Discovery Rate
GBA: Glucocerebrosidase
GO: Gene Ontology
GRN: Gene Regulatory Network
HMGB1: High Mobility Group Box 1
IKAP: Iterative K-means clustering with Adaptive Peaks
LCSB: Luxembourg Centre for Systems Biomedicine
MDK: Midkine
NF-κB: Nuclear Factor Kappa B
PD: Parkinson’s Disease
RAGE: Receptor for Advanced Glycation End Products
RBP: RNA-Binding Protein
RNA: Ribonucleic Acid
scRNA: seq-Single-cell RNA Sequencing
TLR4: Toll-like Receptor 4
UMAP: Uniform Manifold Approximation and Projection
