# Supplementary material for: Single-cell analysis reveals shared and distinct molecular signatures in brain organoid models of neurodegeneration and neuroinflammation
Source: Alzheimers Res Ther. 2025 Nov 29;18:1. doi: 10.1186/s13195-025-01926-0 (PMC12771709; doi:10.1186/s13195-025-01926-0)
Supplement: Supplementary file 1 — Supplementary Material 1. [file 13195_2025_1926_MOESM1_ESM.pdf]

Supplementary material to “Single-cell analysis reveals shared and distinct molecular signatures in brain organoid models of Alzheimer's and Parkinson's disease”

Sophie Le Bars, PhD [1], Mohamed Soudy, MS [1], Sarah Louise Nickels, PhD[2] , Jens Christian Schwamborn, PhD [2] and Enrico Glaab, PhD [1]†✉

[1] Biomedical Data Science Group, Luxembourg Centre for Systems Biomedicine (LCSB), University of Luxembourg, Esch-sur-Alzette, Luxembourg

[2] Developmental and Cellular Biology Group, Luxembourg Centre for Systems Biomedicine (LCSB), University of Luxembourg, Esch-sur-Alzette, Luxembourg

Corresponding author:

Enrico Glaab, PhD
Luxembourg Centre for Systems Biomedicine
University of Luxembourg
Tel. +352 621 621 6186
E-mail: enrico.glaab@uni.lu

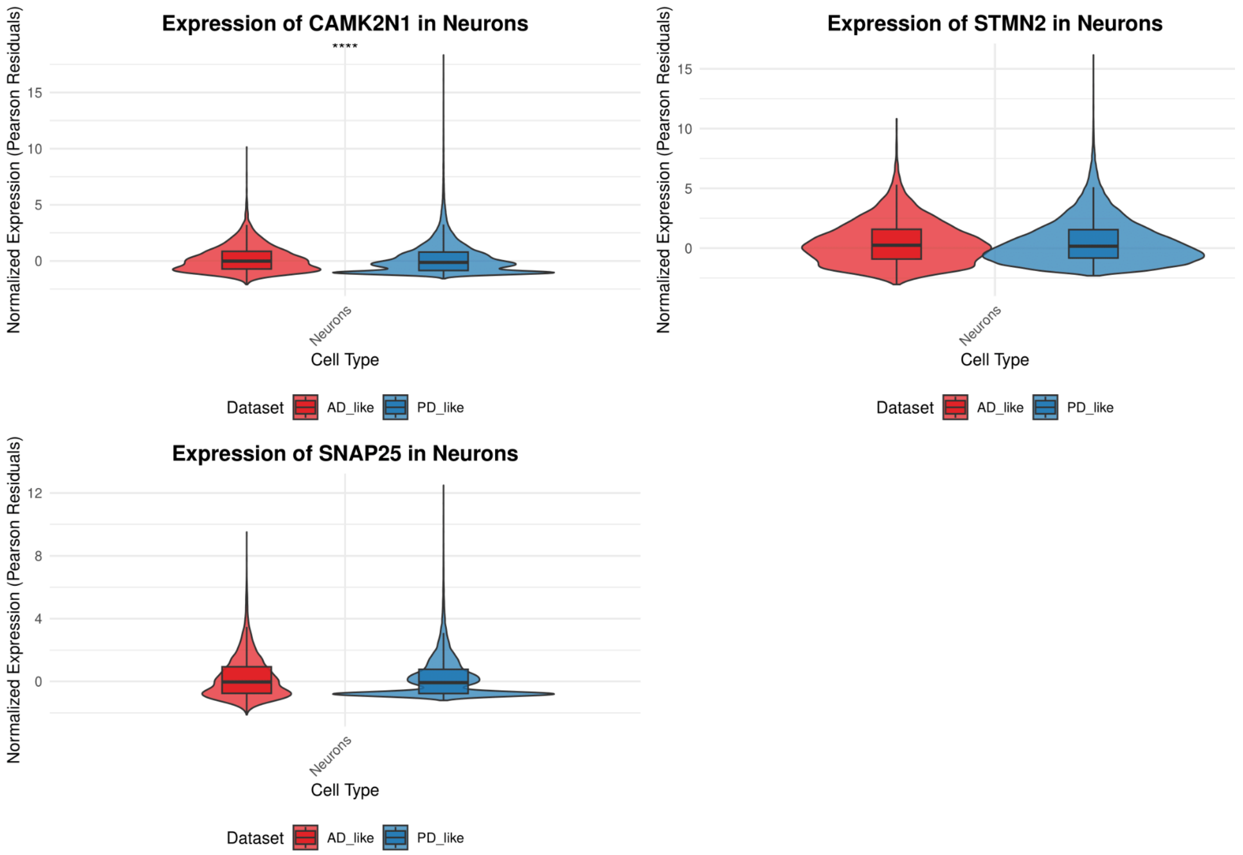

Fig. S1. Dodged violin plots showing the normalized expression (Pearson residuals) of neuronal marker genes (from the Panglao database) in single-cell RNA-seq data from Parkinson's-like (PD-like, blue) and Alzheimer's-like (AD-like, red) brain organoids. Boxplots within the violins indicate expression distribution, and statistical significance was assessed using the Wilcoxon test. A significant difference was only observed for *CAMK2N1* (****: p<=0.0001), whereas no significant differences were detected for *STMN2* (p=0.1) and *SNAP25* (p=0.55).

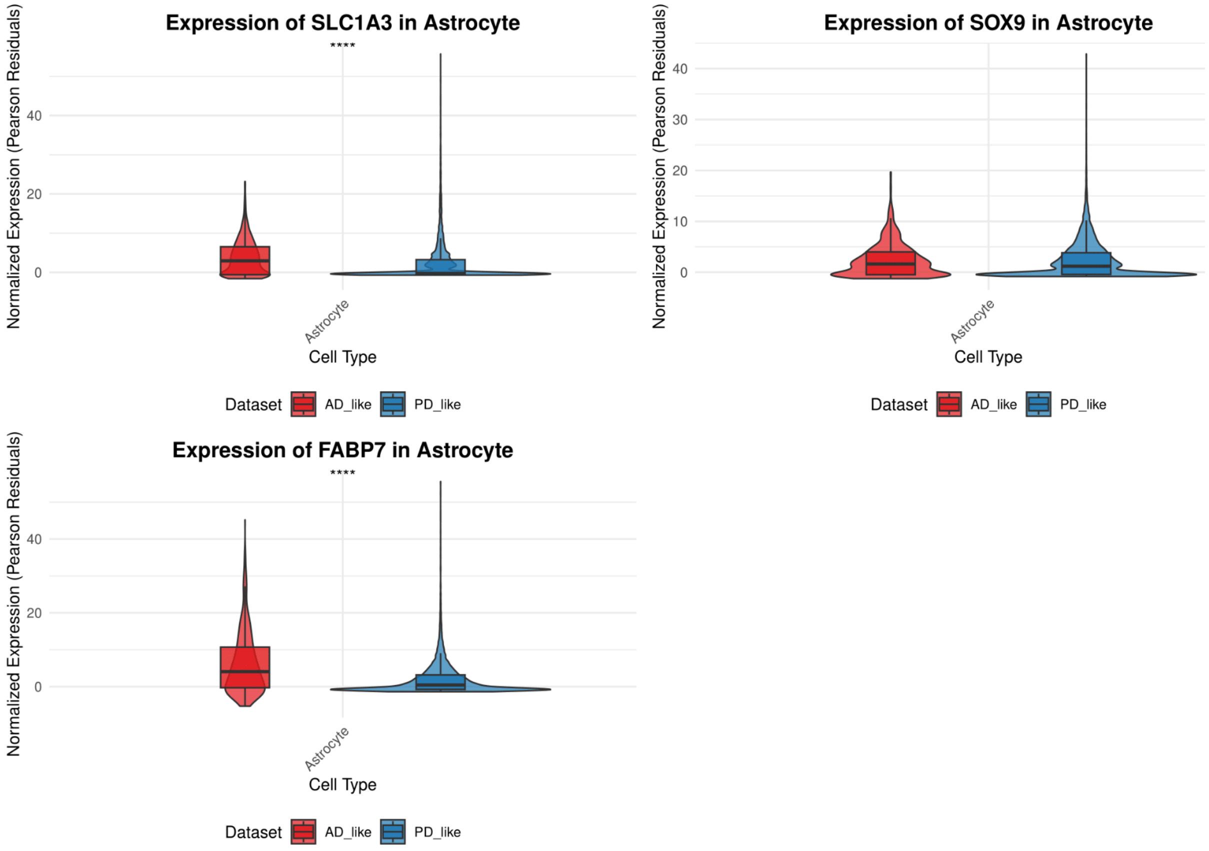

Fig. S2. Dodged violin plots showing the normalized expression (pearson residuals) of astrocyte marker genes (from the Panglao database) in single-cell RNA-seq data from Parkinson's-like (PD-like, blue) and Alzheimer's-like (AD-like, red) brain organoids. Boxplots within the violins indicate expression distribution, and statistical significance was assessed using the Wilcoxon test. Significant differences were observed for *SLC1A3* and *FABP7* (****: $p<=0.0001$), but not for *SOX9* ($p=0.72$).

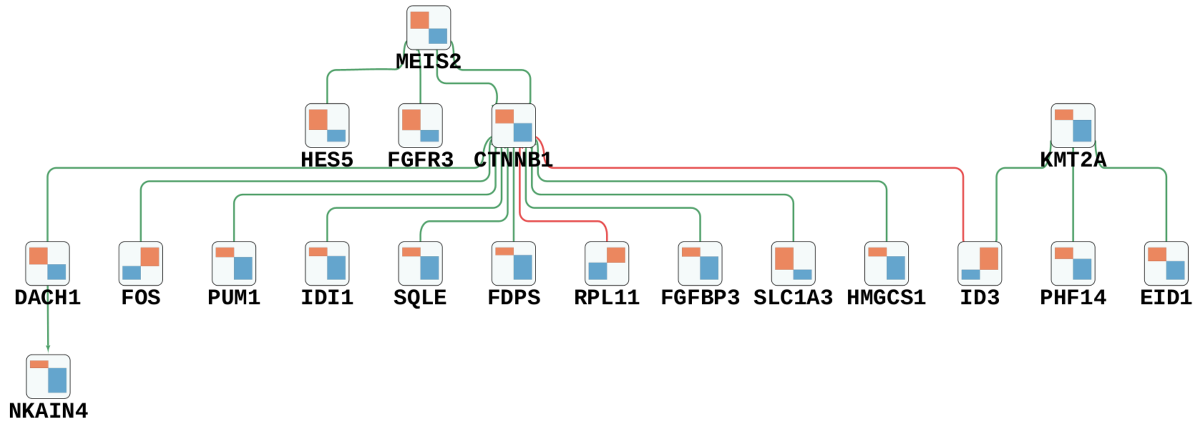

Fig. S3. Visualisation of the gene regulatory subnetwork for the DEGs with contrasting direction of change between AD and PD in astrocytes. Arrows for activating interactions are highlighted in green, inhibiting interactions are highlighted in red. The coloured bars in the nodes represent the condition-specific gene expression changes, left for PD and right for AD; increases are highlighted in orange and decreases in blue.

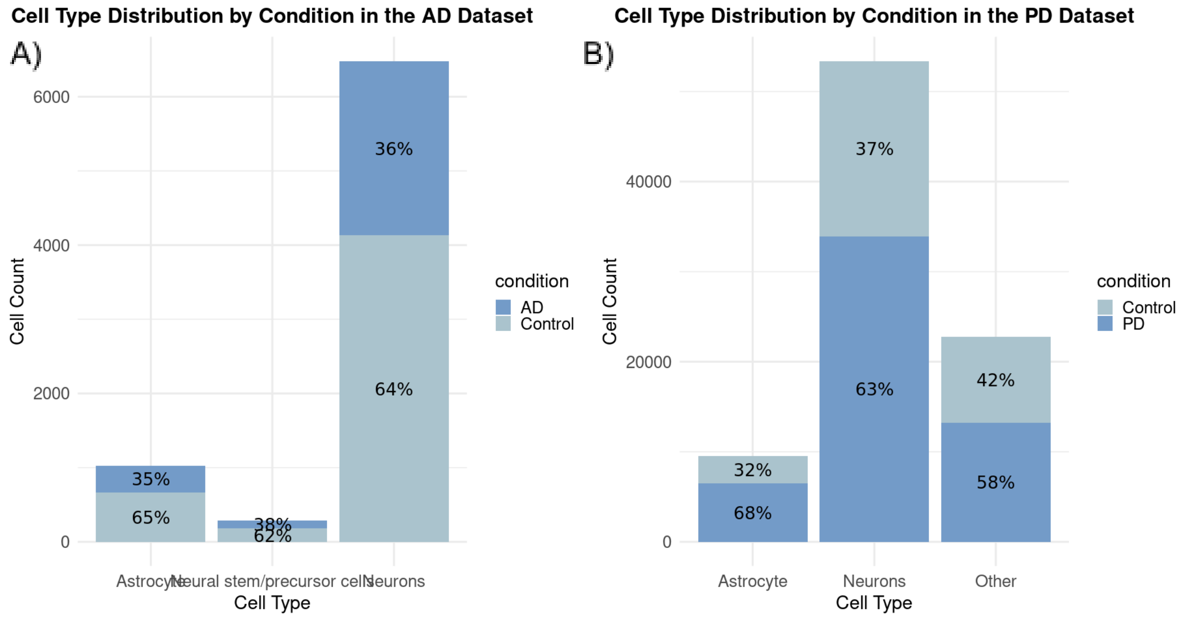

Fig. S4. Cellular composition and experimental condition distribution in AD and PD organoid datasets. Stacked bar charts showing the distribution of experimental conditions across major cell types in (A) AD dataset and (B) PD dataset. Each bar represents the total cell count for a given cell type, with segments indicating the proportion of cells from control (dark blue) versus treatment conditions (light blue: AD_serum for panel A, PD mutant for panel B). Percentages within each segment show the relative contribution of each condition to that cell type. The AD dataset shows balanced distribution across astrocytes (65% control, 35% treated), neural stem/precursor cells (62% control, 38% treated), and neurons (64%

control, 36% treated). The PD dataset demonstrates a more unbalanced distribution across astrocytes (32% control, 68% treated), neurons (37% control, 63% treated), and other cell types (42% control, 58% treated). Note that neural stem/precursor cells were only detected in the AD dataset. For the AD data, Chi-squared analysis confirmed that the distribution of experimental conditions across clusters did not significantly deviate from expected random distribution (χ^2 = 0.99, df = 2, p = 0.61). However, for the PD data, cell type distribution differs significantly between HC and PD (χ^2 = 343.86, df = 2 , p < 2.2e-16). For this reason, we analyzed PD vs. control differences separately for each cell type.

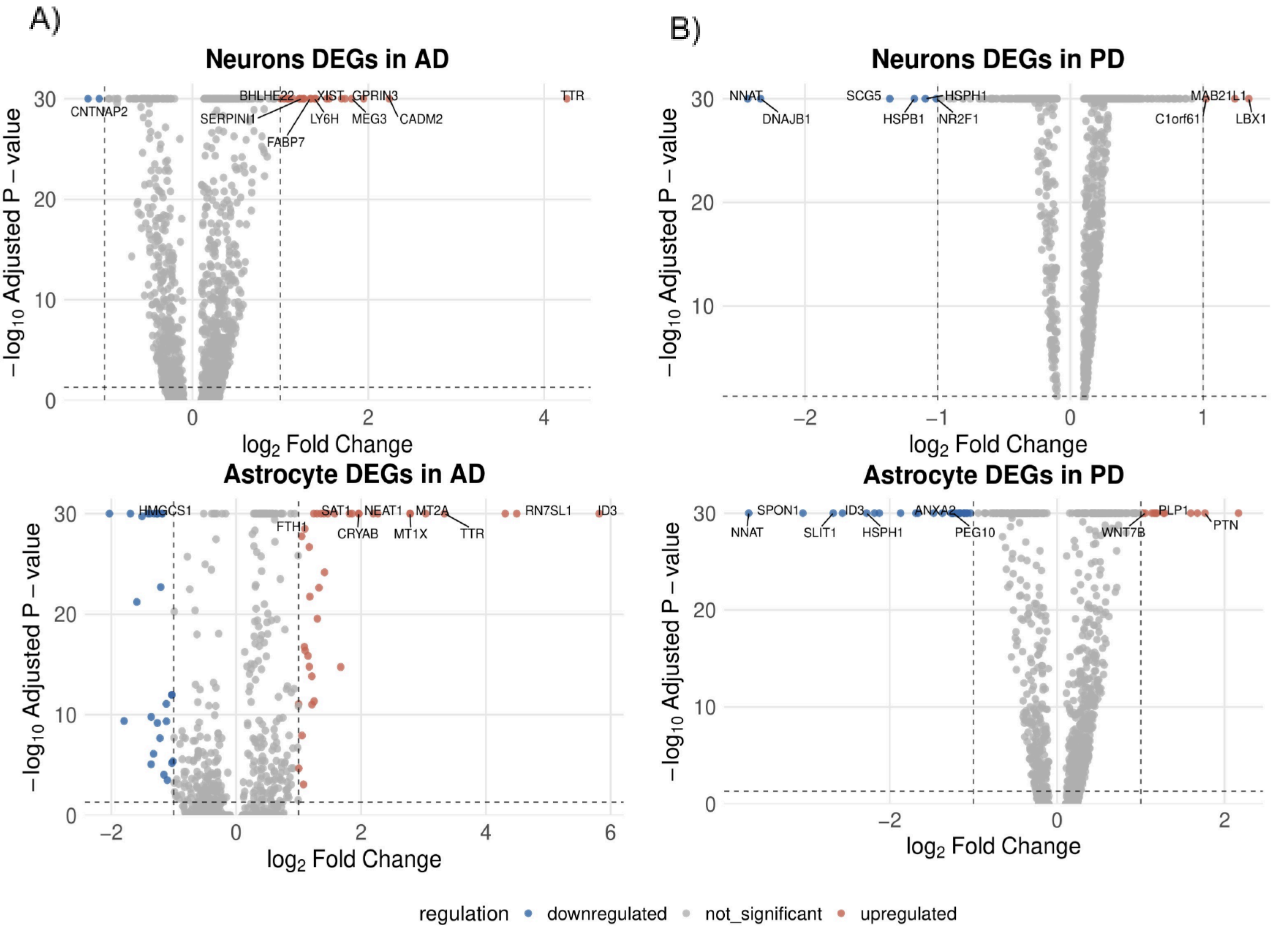

Fig. S5. Volcano plots for differential expression analysis. Volcano plots showing log2 fold change versus -log10(adjusted p-value) for each cell type in (A) AD dataset and (B) PD dataset. Red points indicate significantly overexpressed genes, blue points indicate significantly underexpressed genes, and gray points represent non-significant changes. Dashed lines indicate significance thresholds (adjusted p-value = 0.05, |log2FC| > 1).

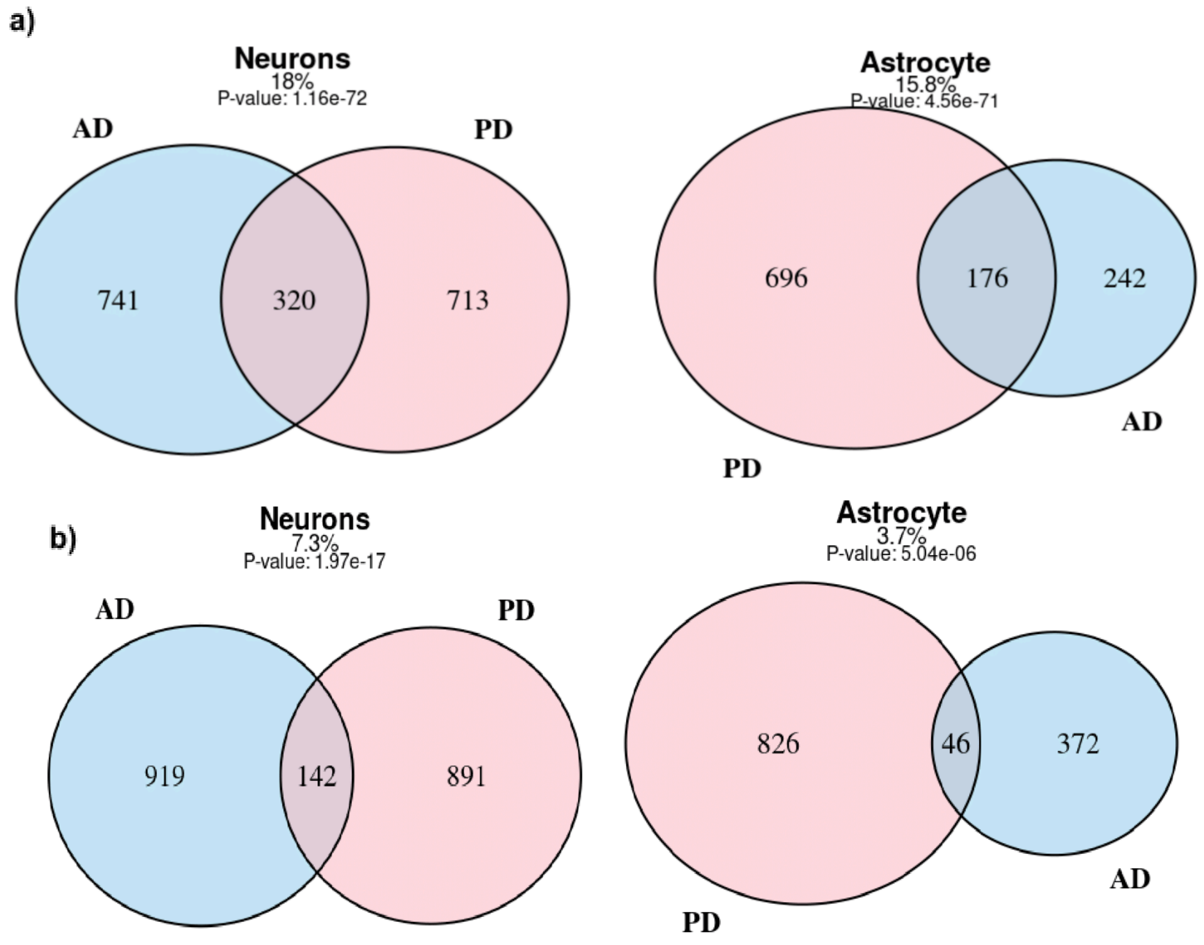

Fig. S6. Venn diagrams of differentially expressed genes (DEGs) overlapping between AD-like and PD-like organoid datasets. (a) **Shared significance irrespective of directionality.** Venn diagrams illustrating the overlap of differentially expressed genes between AD and PD conditions for the shared cell type clusters for neurons (left) and astrocytes (right). Numbers within circles indicate the count of unique DEGs for each condition, while the overlapping region shows shared DEGs between conditions. Percentages represent the proportion of shared DEGs relative to the total DEGs for each cell type (18% for neurons: 320 shared out of 1,774 total DEGs; 15.8% for astrocytes: 176 shared out of 1,114 total DEGs). Blue circles represent AD-specific DEGs, pink circles represent PD-specific DEGs. For neurons: 741 AD-unique, 320 shared, 713 PD-unique DEGs. For astrocytes: 242 AD-unique, 176 shared, 696 PD-unique DEGs. (b) **Shared significance with concordant directionality.** Venn diagrams as in (a), but restricted to DEGs showing the same direction of change (under- or over-expressed) in both conditions. Shared DEGs account for 7.3% of neuronal DEGs and 3.7% of astrocytic DEGs. Blue circles = AD-specific DEGs; pink circles = PD-specific DEGs. For neurons: 919 AD-unique, 142 shared, 891 PD-unique DEGs. For astrocytes: 826 AD-unique, 46 shared, 372 PD-unique DEGs.

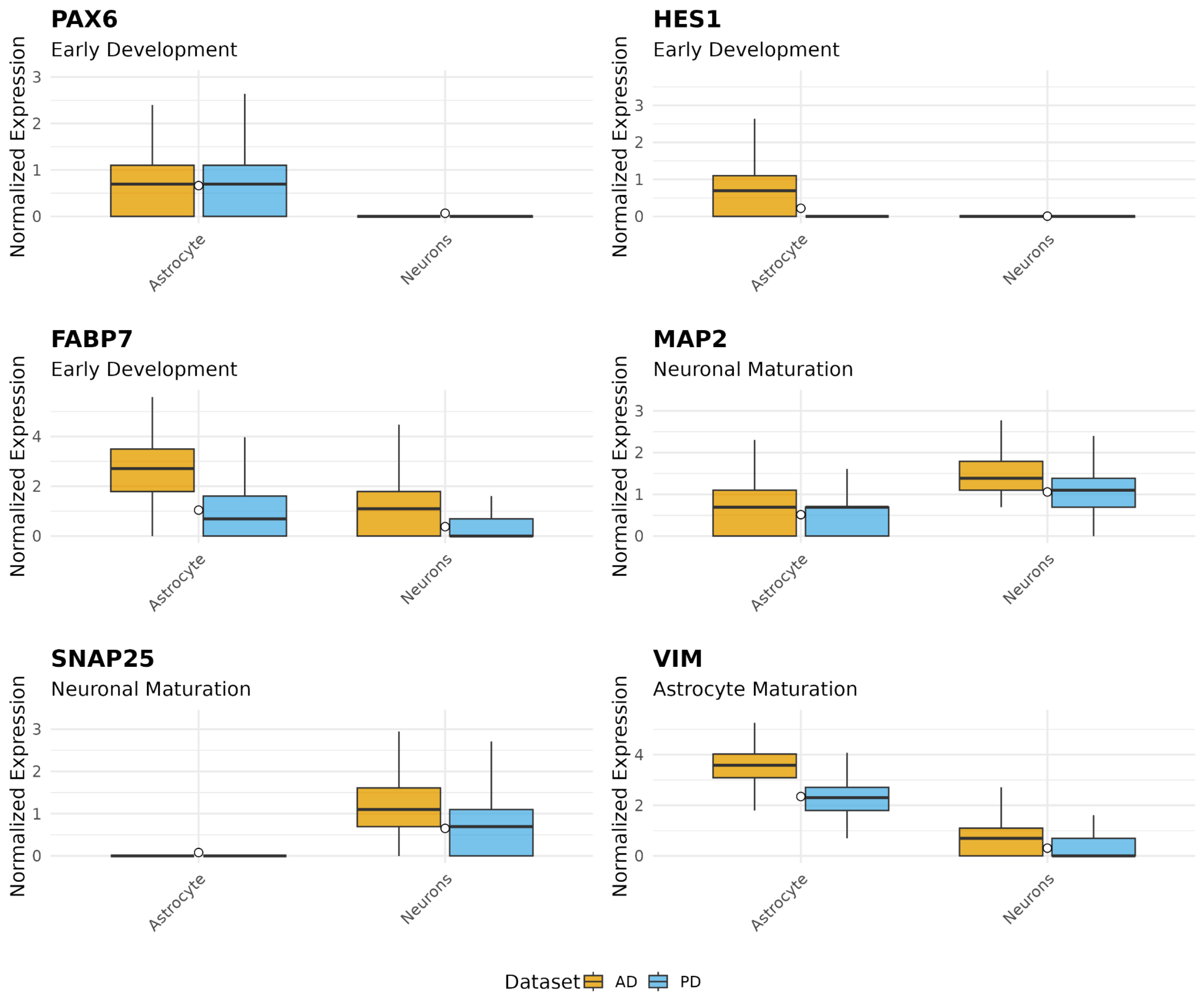

Fig. S7. Developmental and maturation marker analysis across datasets. Expression analysis of developmental stage markers comparing AD (Day 94-95) and PD (Day 30) organoid datasets. Box plots show expression distributions of key developmental and maturation markers across astrocytes and neurons in both datasets. **Early developmental markers** (PAX6, HES1, FABP7) are expressed across cell types in both datasets and show higher levels of expression in AD organoids. **Neuronal maturation markers** (MAP2, SNAP25) demonstrate cell-type specificity with predominant expression in neurons and generally comparable or slightly elevated levels in the more mature AD organoids. **Astrocyte maturation marker analysis** (VIM) shows astrocyte-specific expression with higher levels in AD organoids, reflecting astrocyte maturation over the extended culture period. These expression patterns confirm expected developmental differences between the 30-day (PD) and 94-95-day (AD) culture time points, demonstrating that both datasets contain biologically relevant cell populations at different maturation stages. The observed developmental signatures are distinct from the disease-associated molecular changes identified in the main pathway analyses (Tab. S5 to S13), supporting the biological relevance of the identified disease-specific alterations rather than developmental confounds. Box plots display median, interquartile ranges, and data distribution; minimal expression in non-target cell types confirms marker specificity.

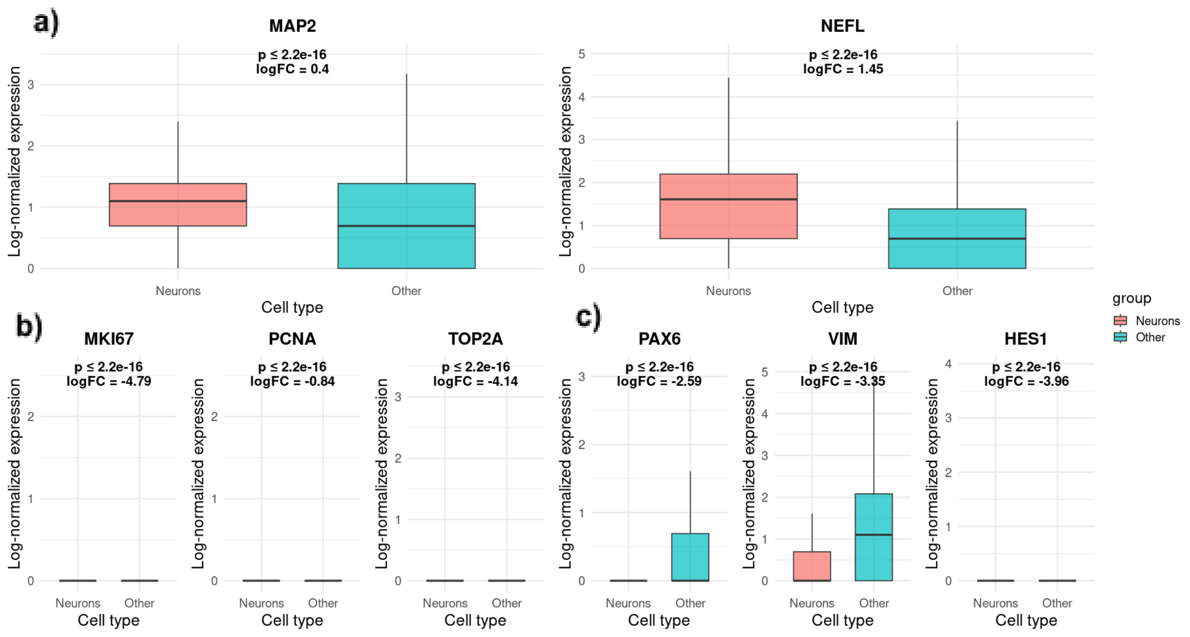

Fig. S8. Neuronal maturity validation in Day 30 PD organoids. Marker gene expression analysis demonstrating neuronal maturity in Day 30 PD organoids. (A) Expression levels of mature neuronal markers (MAP2, NEFL) in identified neuronal clusters. (B) Expression of proliferation markers (MKI67, PCNA, TOP2A) showing minimal proliferative activity. (C) Comparison with neuronal progenitor markers (VIM, PAX6, HES1) demonstrating post-mitotic status. Scale bars represent normalized expression values, with statistical significance indicated by p-value.

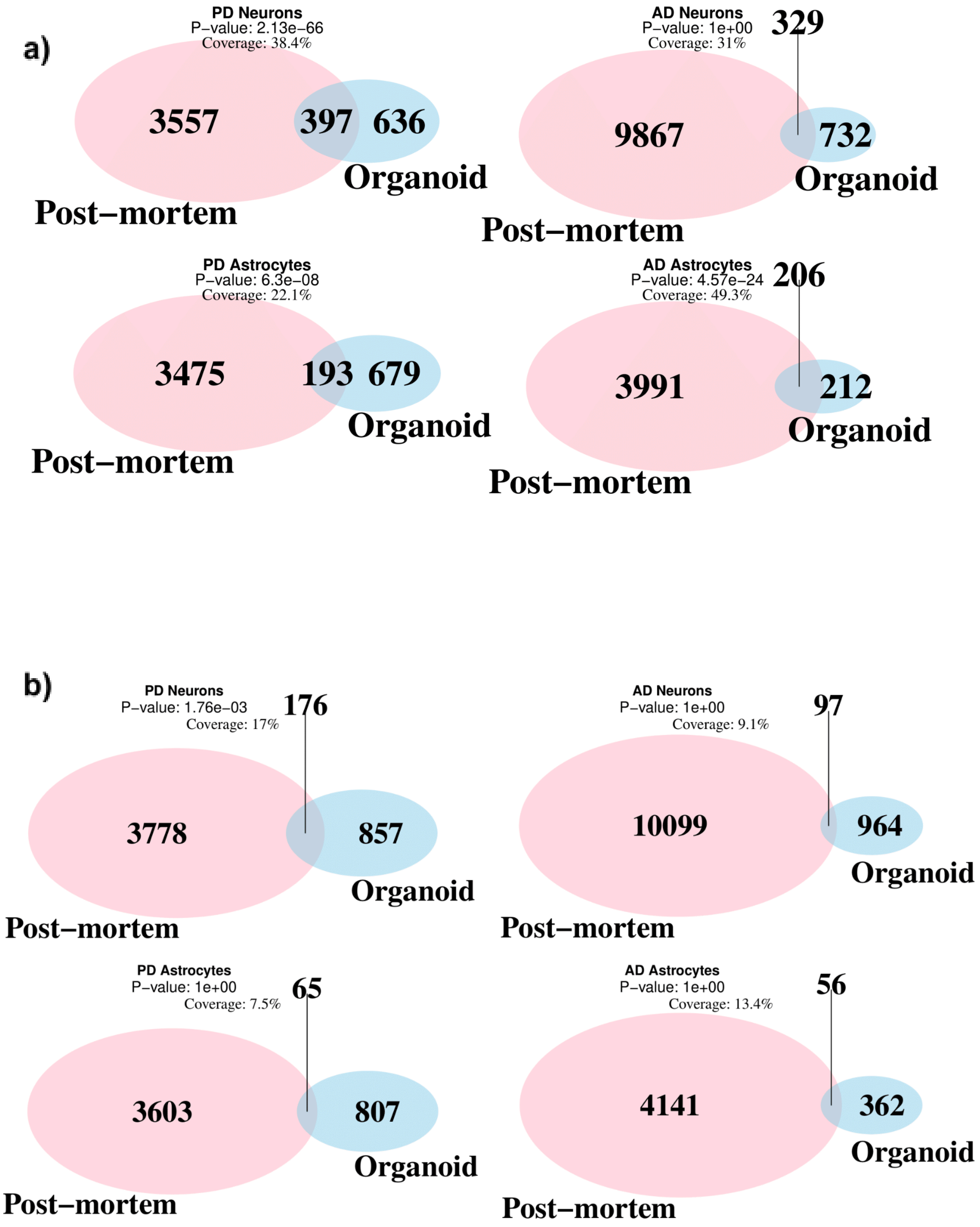
a)
PD Neurons
P–value: 2.13e–66
Coverage: 38.4%
3557
397
636
Organoid
Post–mortem
AD Neurons
P–value: 1e+00
Coverage: 31%
329
9867
732
Organoid
Post–mortem
PD Astrocytes
P–value: 6.3e–08
Coverage: 22.1%
3475
193
679
Organoid
Post–mortem
AD Astrocytes
P–value: 4.57e–24
Coverage: 49.3%
206
3991
212
Organoid
Post–mortem
b)
PD Neurons
P–value: 1.76e–03
Coverage: 17%
176
3778
857
Organoid
Post–mortem
AD Neurons
P–value: 1e+00
Coverage: 9.1%
97
10099
964
Organoid
Post–mortem
PD Astrocytes
P–value: 1e+00
Coverage: 7.5%
65
3603
807
Organoid
Post–mortem
AD Astrocytes
P–value: 1e+00
Coverage: 13.4%
56
4141
362
Organoid
Post–mortem

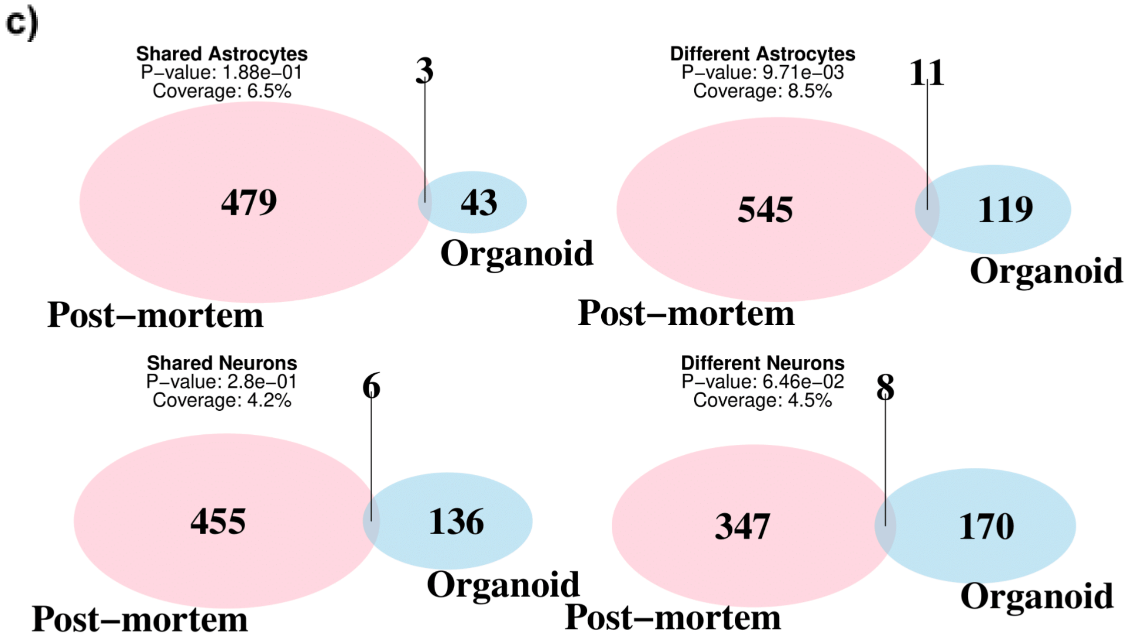

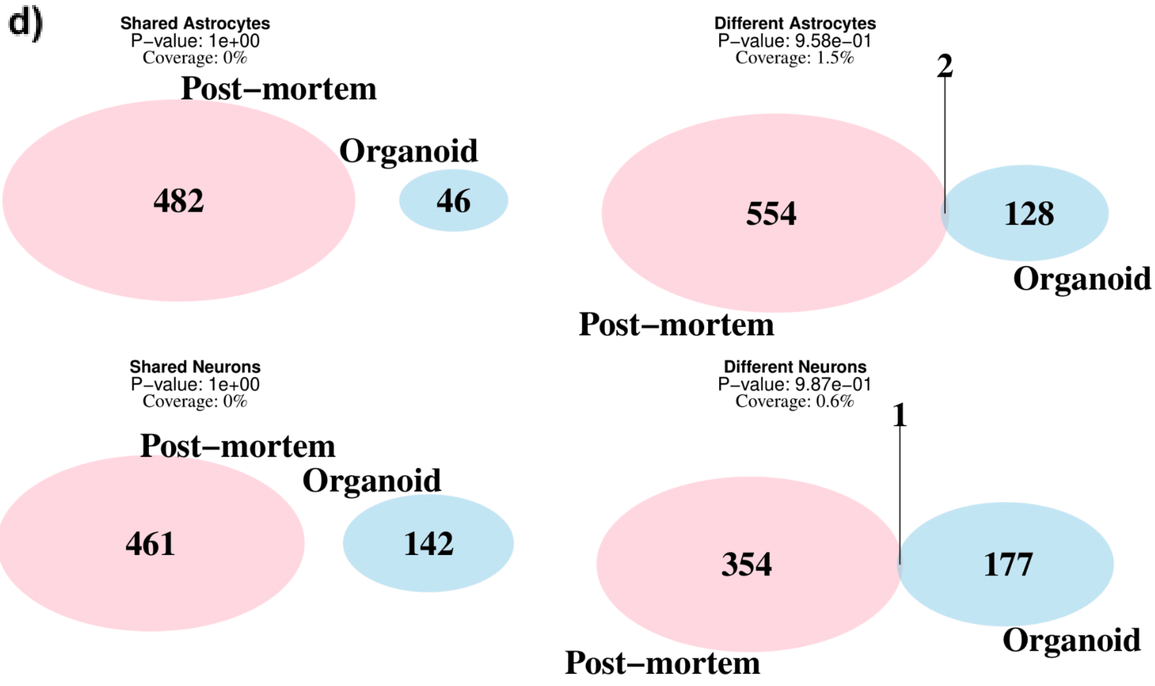

Fig. S9. Venn diagrams of differentially expressed genes (DEGs) overlapping between organoid and *post-mortem* datasets. (a) Disease-specific overlap analysis - genes with shared significance irrespective of directionality: Venn diagrams illustrating the overlap of DEGs between organoid models and *post-mortem* tissue data for each disease and cell type combination. Top panels show neuronal comparisons: PD neurons (left) with 397 shared

DEGs representing 38.4% coverage of organoid DEGs, and AD neurons (right) with 329 shared DEGs representing 31.0% coverage. Bottom panels show astrocyte comparisons: PD astrocytes (left) with 193 shared DEGs representing 22.1% coverage, and AD astrocytes (right) with 206 shared DEGs representing 49.3% coverage. Numbers within circles indicate unique DEGs for each experimental system, while overlapping regions show shared DEGs. Blue circles represent organoid-specific DEGs, pink circles represent post-mortem-specific DEGs. Fisher's exact test p-values show that there is statistically significant overlap in three out of four comparisons (PD neurons: p = 2.13E-66; AD neurons: p = 1; PD astrocytes: p = 6.3E-08; AD astrocytes: p = 4.57E-24). **(b) Disease-specific overlap analysis – genes with shared significance and concordant directionality.** Venn diagrams as in (a), but restricted to DEGs showing the same direction of change (under- or over-expressed) in both organoid and post-mortem datasets. Top panels show neuronal comparisons: PD neurons (left) with 176 shared DEGs (17% of organoid DEGs) and AD neurons (right) with 97 shared DEGs (9.1%). Bottom panels show astrocyte comparisons: PD astrocytes (left) with 65 shared DEGs (7.5%) and AD astrocytes (right) with 56 shared DEGs (13.4%). Numbers within circles denote unique DEGs for each dataset, and overlapping regions indicate shared DEGs. Blue circles represent organoid-specific DEGs; pink circles represent post-mortem-specific DEGs. Fisher's exact test p-values indicate statistically significant overlap for PD neurons (p = 1.76E-03); all other comparisons were not significant (AD neurons: p = 1; PD astrocytes: p = 1; AD astrocytes: p = 1).

(c) Cross-disease signature overlap analysis (stringent analysis) - genes with shared significance irrespective of directionality: Venn diagrams showing overlap between organoid and *post-mortem* datasets for genes previously identified as either shared (concordant expression changes between AD and PD) or contrasting (discordant expression changes between diseases) in organoid models. Analysis reveals the following overlaps: shared astrocytes (6.5% coverage, 3 overlapping genes, p = 1.88E-01), contrasting astrocytes (8.5% coverage, 11 overlapping genes, p = 9.71E-03), shared neurons (4.2% coverage, 6 overlapping genes, p = 2.8E-01), and contrasting neurons (4.5% coverage, 8 overlapping genes, p = 6.46E-02). The limited overlap in this stringent analysis emphasizes the challenge of validating cross-disease molecular signatures across different experimental systems and disease stages. **(d) Cross-disease signature overlap analysis (stringent analysis) – genes with shared significance and concordant directionality.** Same as (c), but restricted to DEGs showing the same direction of change (under- or over-expressed) in both organoid and post-mortem datasets. Analysis reveals the following overlaps: shared astrocytes (0% coverage), contrasting astrocytes (1.5% coverage, 2 overlapping genes, p = 9.58E-01), shared neurons (0% coverage), and contrasting neurons (0.6% coverage, 1 overlapping gene, p = 9.87E-01). The complete list of differentially expressed genes (DEGs) from *post mortem* (PMT) and organoid datasets is provided in the GitLab repository associated with the manuscript.

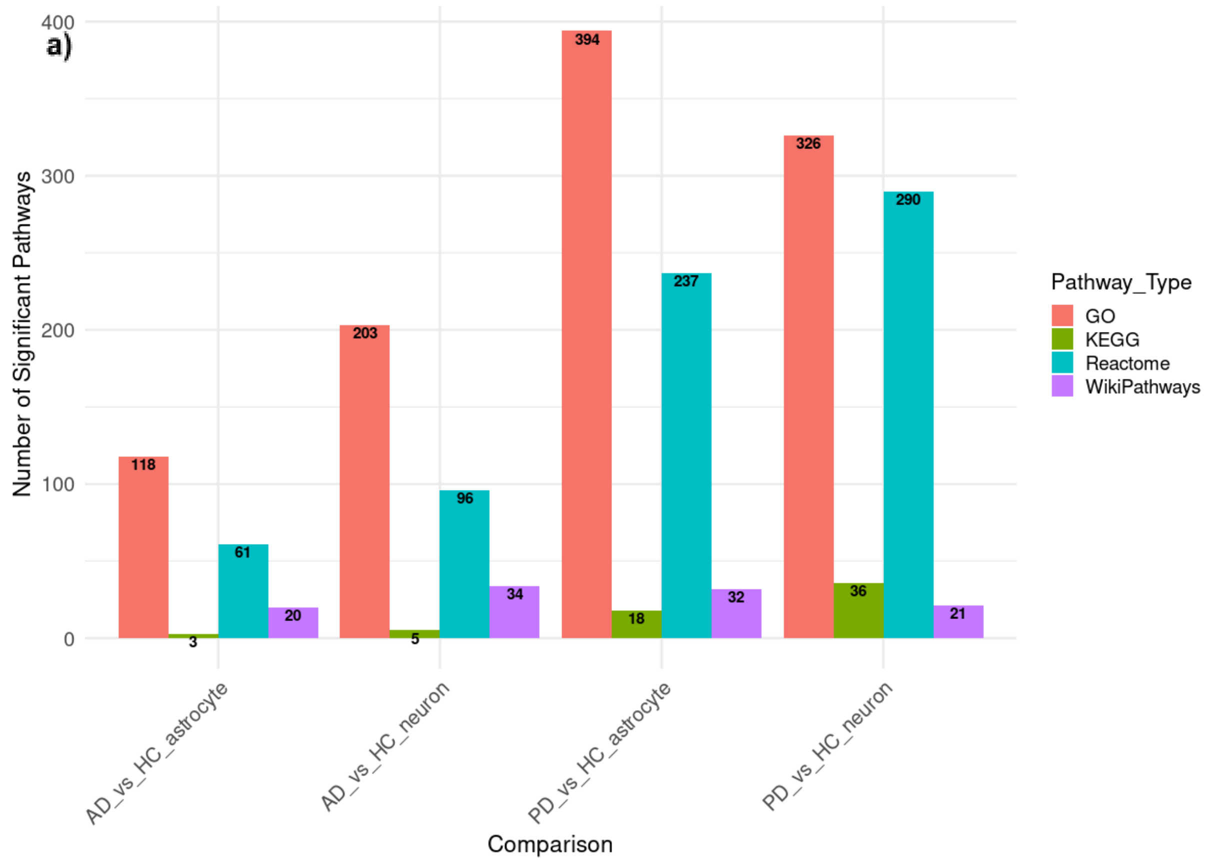
a)
Number of Significant Pathways
400
300
200
100
0
394
326
290
237
203
118
96
61
36
34
32
21
20
18
5
3
AD_vs_HC_astrocyte
AD_vs_HC_neuron
PD_vs_HC_astrocyte
PD_vs_HC_neuron
Comparison
Pathway_Type
GO
KEGG
Reactome
WikiPathways

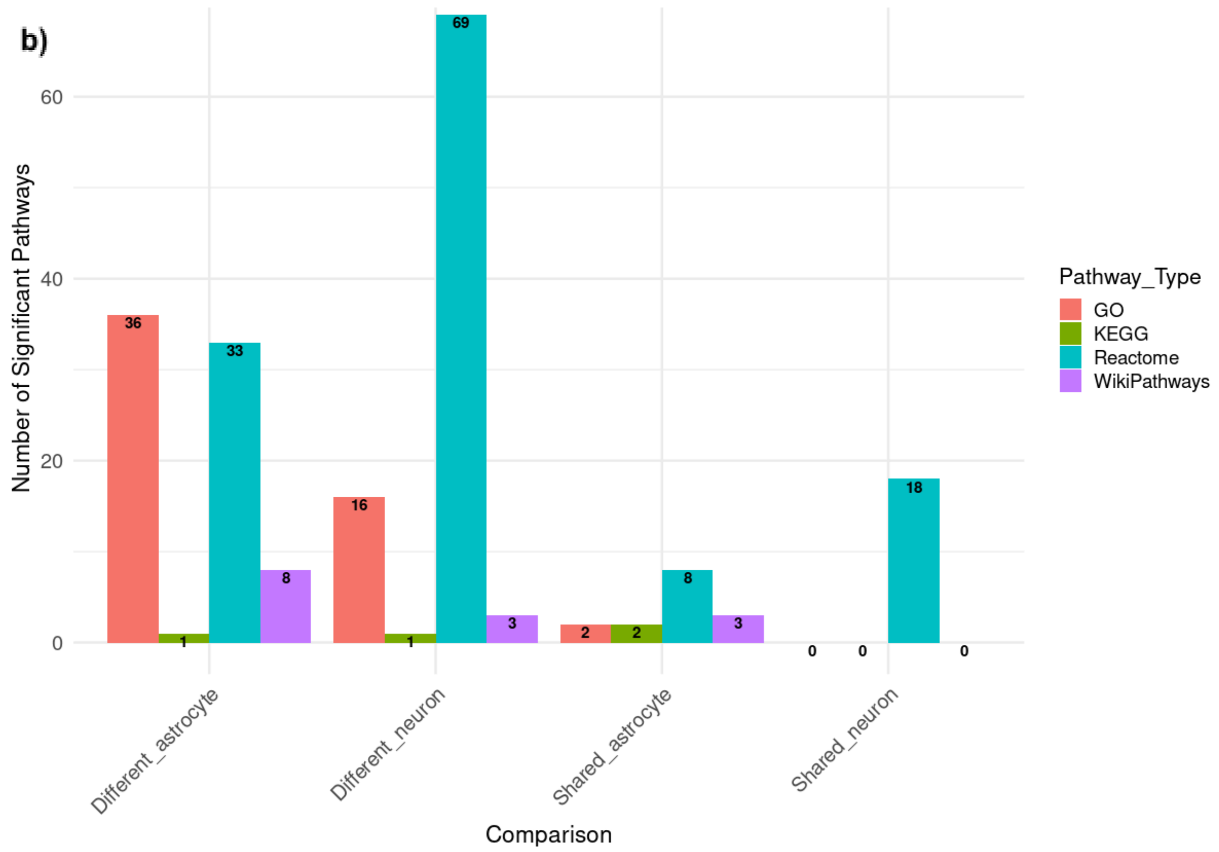
b)
Number of Significant Pathways
60
40
20
0
69
36
33
18
16
8
8
3
3
2
2
1
1
0
0
0
Different_astrocyte
Different_neuron
Shared_astrocyte
Shared_neuron
Comparison
Pathway_Type
GO
KEGG
Reactome
WikiPathways

Fig. S10. Pathway enrichment summary statistics across diseases and databases. Bar plots showing the number of significantly enriched pathways (FDR < 0.05) identified across different pathway databases and comparisons. **(a)** The top plot shows the **disease-specific pathway enrichment,** comparing AD vs. control and PD vs. control for astrocytes and neurons across the pathway databases Gene Ontology (GO) Biological Process, KEGG, Reactome, and WikiPathways. **(b) The bottom plot contains the cross-disease pathway analysis,** showing enrichment for shared DEGs (genes with concordant expression changes between AD and PD) and contrasting DEGs (genes with discordant expression changes) in astrocytes and neurons. Numbers on bars indicate the exact count of significantly enriched pathways for each comparison. The GO Biological Process database consistently shows the highest number of enriched pathways across all comparisons in line with the larger size of this database, while KEGG and Reactome databases provide more focused pathway sets. Cross-disease analysis reveals that contrasting DEGs show more extensive pathway enrichment than shared DEGs, suggesting distinct disease-specific molecular mechanisms alongside common neurodegenerative processes. Pathway rankings with statistical details are provided in Supplementary Tables S5-S12 (for the significant pathways only up to a maximum of 50 top-ranked pathways) and the GitLab repository associated with the manuscript.

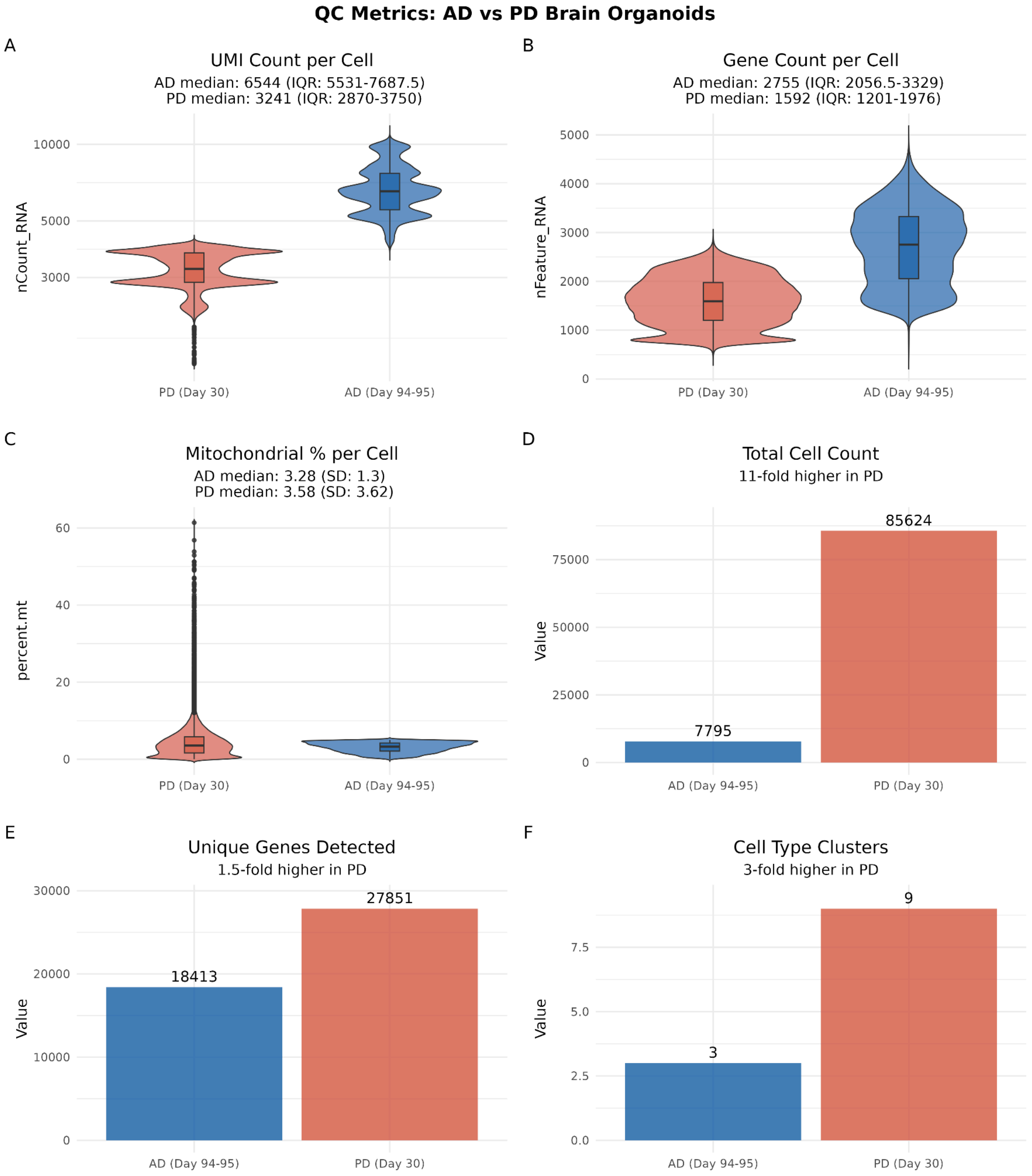

Fig. S11. Technical Differences Between AD and PD Organoid Datasets. Side-by-side comparison of key technical metrics between AD and PD brain organoid datasets demonstrating methodological differences that may influence cross-disease comparisons. Top left: Distribution of unique molecular identifiers (UMIs) per cell, showing 2-fold higher sequencing depth in AD organoids (median: 6,544) compared to PD organoids (median: 3,241). Top right: Distribution of genes detected per cell, with AD organoids showing 1.7-fold higher gene detection per cell (median: 2,755) than PD organoids (median: 1,592). Middle left: Mitochondrial read percentages, indicating higher mitochondrial stress signatures in PD organoids (median: 3.58% ± 3.62%) compared to AD organoids (median: 3.28% ± 1.3%). Middle right: Total cell count comparison revealing an 11-fold imbalance between datasets (PD: 85,624 cells vs AD: 7,795 cells), creating differences in statistical power for

downstream analyses. Bottom left: Total unique genes detected across each dataset, with PD detecting 1.5-fold more genes (27,851) than AD (18,413), likely reflecting the larger cell population. Bottom right: Number of resolved cell type clusters, showing 3-fold higher resolution in the PD dataset (9 clusters) compared to AD (3 clusters), potentially due to enhanced statistical power from larger sample size. These technical differences highlight the challenges in direct cross-disease comparisons and underscore the hypothesis-generating nature of the comparative analyses presented in this study. Violin plots show kernel density distributions with embedded box plots indicating median and interquartile ranges; bar charts show total counts with fold-change differences indicated (IQR = interquartile range; SD = standard deviation).

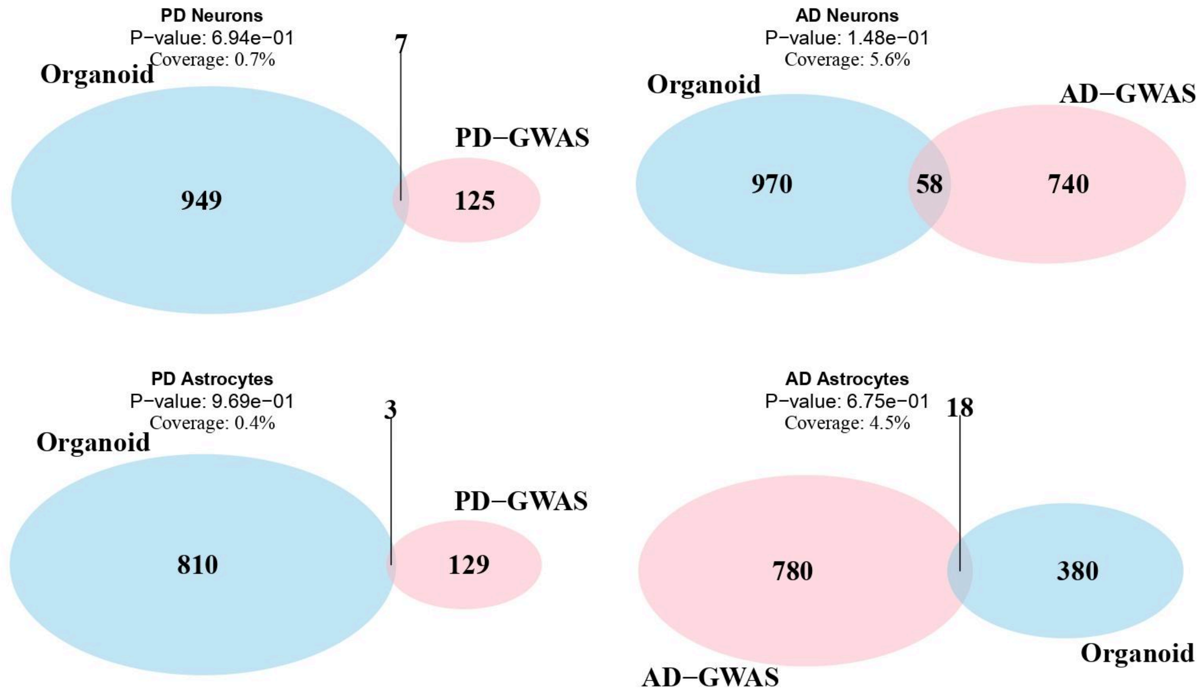

Fig. S12. Venn diagrams of differentially expressed genes (DEGs) in the organoid datasets overlapping with significant *GWAS* genes for AD and PD. Venn diagrams showing the overlap between differentially expressed genes (DEGs) from brain organoid models and genome-wide association study (GWAS) loci, studied separately for Alzheimer's disease (AD) and Parkinson's disease (PD). Top panels: neuronal DEGs (PD neurons, left; AD neurons, right). PD neurons share 7 DEGs with PD-GWAS genes (0.7% of organoid DEGs), while AD neurons share 58 DEGs with AD-GWAS (5.6%). Bottom panels: astrocytic DEGs (PD astrocytes, left; AD astrocytes, right). PD astrocytes share 3 DEGs with PD-GWAS genes (0.4%), and AD astrocytes share 18 DEGs with AD-GWAS genes (4.5%). Numbers within circles indicate unique DEGs per dataset, and overlaps indicate shared DEGs. Blue circles = organoid-specific DEGs; pink circles = GWAS-identified genes

(genome-wide significant SNPs). Fisher's exact test p-values indicate no statistically significant overlap in any comparison. No four-group overlaps between the datasets were observed (i.e. AD-GWAS genes overlapping with both PD-GWAS and AD/PD-organoid DEGs). Three-group overlaps between the datasets are reported in Tab. ST17.

Characteristic	Alzheimer's Disease Dataset	Parkinson's Disease Dataset
Origin	Human cortical fibroblasts	Human midbrain fibroblasts
Culture duration	94-95 days	30 days
Experimental groups	Serum-treated (n=2), Control (n=2)	PD patients (n=3), Healthy controls (n=3)
scRNA-seq platform	10x Genomics Chromium	GEXSCOPE Single Cell Library Prep 3'
Sequencing platform	Illumina NovaSeq 6000	Illumina NovaSeq
Sequencing configuration	Paired-end (Read 1: 28 cycles, Read 2: 101 cycles, Index read: 8 cycles)	Paired-end, 2x150 bp
Disease model	Serum-induced BBB breakdown	Patient-derived organoids

Tab. S1. Comparative Characteristics of AD and PD scRNA-seq Datasets.This table presents a side-by-side comparison of key features from two single-cell RNA sequencing (scRNA-seq) datasets derived from brain organoids modeling Alzheimer's disease (AD) and Parkinson's disease (PD). It highlights differences in organoid origin, culture duration, experimental design, sequencing methods and disease modelling approaches between the two studies**.**

Cell Type	Total Cells	Control Cells	Serum-treated Cells	% Control	% Serum-treated	Control-to-Treated Ratio
Astrocyte	1028	668	360	65%	35%	1.86
NSCs	285	177	108	62%	38%	1.64
Neurons	6482	4130	2352	64%	36%	1.76

Tab. S2. Cellular composition and experimental condition distribution in the AD brain organoid dataset. Detailed cell counts and percentages showing the distribution of cells across experimental conditions (control vs. serum-treated) for each identified cell type in the Alzheimer's disease brain organoid dataset. The columns include: (1) Cell type annotations (NSCs = Neural Stem Cells), (2) Total cell count per cell type, (3) Number of cells from

control samples, (4) Number of cells from serum-treated samples, (5) Percentage of cells from each condition, and (6) Control-to-treated ratio. Chi-squared test results ($\chi^2 = 0.99$, df = 2, p = 0.61) confirm no significant deviation from expected random condition distribution across clusters, indicating that clustering reflects biological cell type diversity rather than condition-specific technical artifacts. Data support the balanced representation of experimental conditions across all major cell types (astrocytes, neural stem/precursor cells, and neurons).

Cell Type	Total Cells	Control Cells	Parkinson's Disease Cells	% Healthy	% PD	Control-to-PD Ratio
Astrocyte	9495	3002	6493	32%	68%	0.46
Neurons	53398	19514	33884	37%	63%	0.58
Other	22731	9511	13220	42%	58%	0.72

Tab. S3. Cellular composition and experimental condition distribution in the PD brain organoid dataset. Comprehensive breakdown of cell counts and percentages across experimental conditions (healthy control vs. GBA-N409S mutation) for each identified cell type in the PD brain organoid dataset. The columns include: (1) Cell type annotations (the category "Other" reflects a cluster with a mixture of marker genes for different cell types, precluding a clear assignment to a single cell type), (2) Total cell count per cell type, (3) Number of cells from healthy control organoids, (4) Number of cells from PD patient-derived organoids with GBA-N409S mutation, (5) Percentage distribution of cells from each condition, and (6) PD-to-control ratio. Chi-squared testing showed that cell type distribution differs significantly between HC and PD ($\chi^2 = 343.86$, df = 2 , $p < 2.2e\text{-}16$) between conditions. The control-to-PD vs. control ratios are 0.46 for astrocytes, 0.58 for neurons, and 0.72 for other cell types. These differences necessitate cell type-specific differential expression analyses to account for the varying cellular composition between experimental groups.

Dataset / Celltype	Organoid DEGs	Post-mortem DEGs	Shared DEGs	Only Organoid	Only Post-mortem	%Coverage Organoid	%Coverage Post-mortem	P-value
AD Neurons	1061	10196	329	732	9867	31	3.23	1
AD Astrocytes	418	4197	206	212	3991	49.3	4.91	4.57E-24
PD Neurons	1033	3954	397	636	3557	38.4	10.04	2.13E-66
PD Astrocytes	872	3668	193	679	3475	22.1	5.26	6.2E-08

Tab. S4. Bidirectional overlap statistics for organoid-postmortem DEG comparisons. Statistics for the differentially expressed gene (DEG) overlap between organoid models and *post-mortem* tissue data. Columns include: Dataset/Cell type, total organoid DEGs, total *post-mortem* DEGs, number of shared DEGs between datasets, organoid-specific DEGs (only significant in organoids), *post-mortem*-specific DEGs (only significant in *post-mortem* tissue), organoid coverage percentage (shared DEGs / total organoid DEGs × 100), post-mortem coverage percentage (shared DEGs / total post-mortem DEGs × 100), and Fisher's Exact test p-value for enrichment significance. Coverage percentages show that organoid DEGs capture 22.1-49.3% of their respective gene sets in post-mortem data, while post-mortem DEGs show 3.23-10.04% reciprocal coverage in organoids, reflecting the different scales and contexts of these experimental systems. According to Fisher's Exact test ($p < 0.05$), three out of four comparisons show statistically significant overlap, with varying effect sizes as indicated by odds ratios.

Pathway	Cell Type	p-value	FDR	Count
Translation Initiation	astrocyte	4.01E-95	7.10E-93	76
Mevalonate Pathway	astrocyte	2.76E-05	0.002	6
Cholesterol Biosynthesis	astrocyte	4.88E-05	0.003	6
Translation Initiation	neuron	1.64E-61	4.05E-59	69
Mutation Caused Aberrant Abeta to Electron Transfer in Complex IV	neuron	5.67E-07	7.02E-05	10
Electron Transfer in Complex IV	neuron	3.62E-06	2.99E-04	9
Env Factor Arsenic to Electron Transfer in Complex IV	neuron	1.30E-05	8.01E-04	9
Mevalonate Pathway	neuron	3.48E-05	0.002	7

Tab. S5. KEGG pathway enrichment analysis for AD dataset. Kyoto Encyclopedia of Genes and Genomes (KEGG) pathway enrichment analysis performed on all significantly differentially expressed genes (DEGs; FDR < 0.05, irrespective of directionality) identified in the AD organoid dataset. Analysis performed using clusterProfiler with over-representation test and Benjamini-Hochberg correction. Columns include: KEGG pathway, cell type, nominal p-value, false-discovery rate (FDR), and gene count. Only pathways with FDR < 0.05 and minimum 5 genes are displayed, ordered by significance within each cell type.

Pathway	Cell Type	p-value	FDR	Count
Translation Initiation	astrocyte	1.59E-35	3.88E-33	51
Mitochondrial Complex UCP1 in Thermogenesis	astrocyte	4.48E-05	2.68E-03	16
Electron Transfer in Complex I	astrocyte	5.77E-05	2.68E-03	12
Electron Transfer in Complex III	astrocyte	6.90E-05	2.68E-03	6
Mutation Caused Aberrant SNCA to Electron Transfer in Complex I	astrocyte	7.69E-05	2.68E-03	12

Mutation Caused Aberrant TDP43 to Electron Transfer in Complex I	astrocyte	7.69E-05	2.68E-03	12
Mutation Inactivated PINK1 to Electron Transfer in Complex I	astrocyte	7.69E-05	2.68E-03	12
Mutation Caused Aberrant SOD1 to 26S Proteasome Mediated Protein Degradation	astrocyte	1.32E-04	3.81E-03	12
Mutation Caused Aberrant HTT to Electron Transfer in Complex III	astrocyte	1.41E-04	3.81E-03	6
Mutation Caused Aberrant Abeta to Electron Transfer in Complex I	astrocyte	3.49E-04	8.05E-03	12
26S Proteasome Mediated Protein Degradation	astrocyte	3.63E-04	8.05E-03	11
Mutation Inactivated UBQLN2 to 26S Proteasome Mediated Protein Degradation	astrocyte	4.62E-04	8.67E-03	11
Mutation Inactivated VCP to 26S Proteasome Mediated Protein Degradation	astrocyte	4.62E-04	8.67E-03	11
KEGG Medicus Pathogen Escherichia EAE TIR TCCP to Actin Signaling Pathway	astrocyte	7.28E-04	1.27E-02	6
Antigen Processing and Presentation by MHC Class I Molecules	astrocyte	2.43E-03	3.95E-02	5
KEGG Medicus Pathogen Escherichia MAP to CDC42 Signaling Pathway	astrocyte	3.66E-03	4.96E-02	5
KEGG Medicus Pathogen Shigella IPAC to Actin Signaling Pathway	astrocyte	3.66E-03	4.96E-02	5
Cholesterol Biosynthesis	astrocyte	3.66E-03	4.96E-02	5
Translation Initiation	neuron	2.98E-21	8.69E-19	40
Mutation Caused Aberrant Abeta to Electron Transfer in Complex I	neuron	5.62E-11	8.20E-09	21
Electron Transfer in Complex I	neuron	1.05E-09	8.72E-08	18
Mutation Caused Aberrant SNCA to Electron Transfer in Complex I	neuron	1.79E-09	8.72E-08	18
Mutation Caused Aberrant TDP43 to Electron Transfer in Complex I	neuron	1.79E-09	8.72E-08	18
Mutation Inactivated PINK1 to Electron Transfer in Complex I	neuron	1.79E-09	8.72E-08	18
Mitochondrial Complex UCP1 in Thermogenesis	neuron	1.21E-06	5.04E-05	19
Mutation Caused Aberrant SOD1 to 26S Proteasome Mediated Protein Degradation	neuron	1.71E-06	6.24E-05	15
26S Proteasome Mediated Protein Degradation	neuron	5.00E-06	1.62E-04	14
Mutation Inactivated UBQLN2 to 26S Proteasome Mediated Protein Degradation	neuron	7.05E-06	1.87E-04	14
Mutation Inactivated VCP to 26S Proteasome Mediated Protein Degradation	neuron	7.05E-06	1.87E-04	14
Mutation Caused Aberrant Abeta to 26S Proteasome Mediated Protein Degradation	neuron	5.70E-05	1.28E-03	12
Scrapie Conformation PrPSc to 26S Proteasome Mediated Protein Degradation	neuron	5.70E-05	1.28E-03	12
Mutation Caused Aberrant Abeta to VGCC Ca2+ Apoptotic Pathway N01006	neuron	9.70E-05	2.02E-03	6
Mutation Caused Aberrant HTT to 26S Proteasome Mediated Protein Degradation	neuron	2.82E-04	5.15E-03	11
Mutation Caused Aberrant SNCA to 26S Proteasome Mediated Protein Degradation	neuron	2.82E-04	5.15E-03	11
Mutation Caused Aberrant Abeta to mGluR5 Ca2+ Apoptotic Pathway	neuron	5.30E-04	9.10E-03	8
Mutation Caused Aberrant SNCA to VGCC Ca2+ Apoptotic Pathway	neuron	6.21E-04	9.55E-03	6
Mutation Inactivated SIGMAR1 to Ca2+ Apoptotic Pathway	neuron	6.21E-04	9.55E-03	6

Scrapie Conformation PrPSc to Transport of Calcium	neuron	7.51E-04	1.10E-02	7
Autophagy Vesicle Nucleation Elongation Maturation Sequestosome 1 Like Receptor	neuron	1.00E-03	1.33E-02	6
Mutation Caused Aberrant Abeta to VGCC Ca2+ Apoptotic Pathway N01004	neuron	1.00E-03	1.33E-02	6
Scrapie Conformation PrPSc to mGluR5 Ca2+ Apoptotic Pathway	neuron	1.10E-03	1.39E-02	7
Transcription Coupled NER	neuron	1.53E-03	1.80E-02	9
Mutation Caused Aberrant Abeta to Transport of Calcium	neuron	1.54E-03	1.80E-02	6
Mutation Caused Aberrant Abeta to mAChR Ca2+ Apoptotic Pathway	neuron	2.15E-03	2.41E-02	7
Mutation Caused Aberrant PSEN to mGluR5 Ca2+ Apoptotic Pathway	neuron	2.28E-03	2.46E-02	6
mGluR5 Ca2+ Apoptotic Pathway	neuron	2.90E-03	3.02E-02	7
PINK PARKIN Mediated Autophagosome Formation	neuron	3.24E-03	3.27E-02	6
Kinetochore Fiber Organization	neuron	3.84E-03	3.62E-02	7
Mutation Caused Aberrant HTT to mGluR5 Ca2+ Apoptotic Pathway	neuron	3.84E-03	3.62E-02	7
Mutation Caused Aberrant ATXN2 3 to mGluR5 Ca2+ Apoptotic Pathway	neuron	4.49E-03	4.08E-02	6
KEGG Medicus Pathogen Escherichia MAP to CDC42 Signaling Pathway	neuron	4.75E-03	4.08E-02	5
Mutation Caused Aberrant PSEN1 to mGluR5 Ca2+ Apoptotic Pathway	neuron	4.75E-03	4.08E-02	5
Mutation Caused Aberrant Abeta to Anterograde Axonal Transport	neuron	5.00E-03	4.17E-02	7
Mutation Caused Aberrant HTT to Transport of Calcium	neuron	6.06E-03	4.91E-02	6

Tab. S6. Kyoto Encyclopedia of Genes and Genomes (KEGG) pathway enrichment analysis performed on all significantly differentially expressed genes (DEGs; FDR < 0.05, irrespective of directionality) identified in the PD organoid dataset. Pathways meeting significance criteria (FDR < 0.05, Count >= 5) are shown, providing insight into metabolic and signaling pathway alterations in GBA-associated neurodegeneration. A maximum of top 50 pathways per cell type are shown.

Pathway	Cell Type	p-value	FDR	Count
Response of EIF2AK4 GCN2 to Amino Acid Deficiency	astrocyte	4.95E-112	4.51E-109	82
Eukaryotic Translation Elongation	astrocyte	6.43E-111	2.93E-108	79
Eukaryotic Translation Initiation	astrocyte	1.07E-99	3.24E-97	81
SRP Dependent Cotranslational Protein Targeting to Membrane	astrocyte	3.90E-99	8.88E-97	79
Selenoamino Acid Metabolism	astrocyte	9.60E-95	1.75E-92	78
Nonsense Mediated Decay NMD	astrocyte	1.03E-93	1.56E-91	77
Cellular Response to Starvation	astrocyte	1.75E-89	2.27E-87	83
Influenza Infection	astrocyte	9.24E-86	1.05E-83	81
Regulation of Expression of SLITs and ROBOs	astrocyte	3.53E-80	3.57E-78	78

Signaling by ROBO Receptors	astrocyte	2.86E-72	2.61E-70	80
rRNA Processing	astrocyte	9.30E-70	7.70E-68	78
Translation	astrocyte	2.37E-64	1.80E-62	85
Metabolism of Amino Acids and Derivatives	astrocyte	4.87E-57	3.41E-55	85
SARS CoV 1 Modulates Host Translation Machinery	astrocyte	1.84E-45	1.20E-43	32
Activation of the mRNA upon Binding of the Cap Binding Complex and eIFs and Subsequent Binding to 43S	astrocyte	2.16E-39	1.31E-37	35
SARS CoV 2 Modulates Host Translation Machinery	astrocyte	8.84E-36	5.03E-34	31
SARS CoV 1 Host Interactions	astrocyte	3.37E-31	1.81E-29	36
SARS CoV 1 Infection	astrocyte	1.16E-24	5.87E-23	36
SARS CoV 2 Host Interactions	astrocyte	6.23E-22	2.99E-20	39
SARS CoV 2 Infection	astrocyte	2.86E-17	1.30E-15	41
SARS CoV Infections	astrocyte	1.20E-13	5.22E-12	46
Cholesterol Biosynthesis	astrocyte	2.35E-12	9.74E-11	12
Regulation of Cholesterol Biosynthesis by SREBP SREBF	astrocyte	2.68E-09	1.06E-07	13
Metabolism of Steroids	astrocyte	1.09E-08	4.15E-07	20
Activation of Gene Expression by SREBF SREBP	astrocyte	1.43E-08	5.21E-07	11
Response of EIF2AK1 HRI to Heme Deficiency	astrocyte	6.87E-08	2.41E-06	7
Neutrophil Degranulation	astrocyte	4.55E-06	1.53E-04	32
Bacterial Infection Pathways	astrocyte	2.69E-05	8.76E-04	10
HSF1 Activation	astrocyte	1.91E-04	6.00E-03	6
Chaperone Mediated Autophagy	astrocyte	3.02E-04	9.11E-03	5
Interleukin 12 Signaling	astrocyte	3.10E-04	9.11E-03	7
Cellular Response to Chemical Stress	astrocyte	3.77E-04	1.07E-02	15
HIV Infection	astrocyte	4.35E-04	1.20E-02	16
Host Interactions of HIV Factors	astrocyte	4.78E-04	1.28E-02	11
The Role of GTSE1 in G2 M Progression after G2 Checkpoint	astrocyte	4.95E-04	1.29E-02	8
Gene and Protein Expression by JAK STAT Signaling after Interleukin 12 Stimulation	astrocyte	6.07E-04	1.49E-02	6
HSF1 Dependent Transactivation	astrocyte	6.07E-04	1.49E-02	6
Nuclear Events Mediated by NFE2L2	astrocyte	6.71E-04	1.61E-02	9
NGF Stimulated Transcription	astrocyte	7.01E-04	1.64E-02	6
Infection with Mycobacterium Tuberculosis	astrocyte	8.24E-04	1.88E-02	5
Attenuation Phase	astrocyte	9.80E-04	2.18E-02	5
Interleukin 12 Family Signaling	astrocyte	1.03E-03	2.22E-02	7
Deactivation of the Beta Catenin Transactivating Complex	astrocyte	1.05E-03	2.22E-02	6
Signaling by ALK in Cancer	astrocyte	1.18E-03	2.42E-02	9
KEAP1 NFE2L2 Pathway	astrocyte	1.20E-03	2.42E-02	10
Aggrephagy	astrocyte	1.35E-03	2.67E-02	6
EGFR Downregulation	astrocyte	1.58E-03	3.07E-02	5
MAPK6 MAPK4 Signaling	astrocyte	1.78E-03	3.21E-02	8

PERK Regulates Gene Expression	astrocyte	1.83E-03	3.21E-02	5
Rho GTPases Activate IQGAPs	astrocyte	1.83E-03	3.21E-02	5
Eukaryotic Translation Elongation	neuron	1.27E-73	1.43E-70	74
SRP Dependent Cotranslational Protein Targeting to Membrane	neuron	2.46E-68	1.38E-65	77
Eukaryotic Translation Initiation	neuron	9.59E-67	3.60E-64	78
Response of EIF2AK4 GCN2 to Amino Acid Deficiency	neuron	1.15E-65	3.23E-63	72
Nonsense Mediated Decay NMD	neuron	6.43E-61	1.45E-58	73
Selenoamino Acid Metabolism	neuron	1.13E-58	2.13E-56	72
Influenza Infection	neuron	1.10E-56	1.78E-54	80
Signaling by ROBO Receptors	neuron	2.51E-54	3.54E-52	88
Regulation of Expression of SLITs and ROBOs	neuron	1.26E-53	1.58E-51	78
Cellular Response to Starvation	neuron	3.39E-53	3.82E-51	77
Translation	neuron	1.08E-49	1.10E-47	99
rRNA Processing	neuron	1.03E-38	9.69E-37	73
Metabolism of Amino Acids and Derivatives	neuron	1.33E-29	1.16E-27	84
Activation of the mRNA upon Binding of the Cap Binding Complex and eIFs and Subsequent Binding to 43S	neuron	2.41E-29	1.94E-27	36
SARS CoV 1 Modulates Host Translation Machinery	neuron	3.44E-29	2.58E-27	29
SARS CoV 2 Modulates Host Translation Machinery	neuron	2.78E-24	1.96E-22	30
SARS CoV 1 Host Interactions	neuron	6.34E-20	4.20E-18	36
SARS CoV 1 Infection	neuron	5.25E-14	3.29E-12	36
Respiratory Electron Transport	neuron	2.67E-11	1.59E-09	33
SARS CoV 2 Host Interactions	neuron	5.05E-11	2.85E-09	39
Aerobic Respiration and Respiratory Electron Transport	neuron	1.60E-10	8.56E-09	43
Complex IV Assembly	neuron	2.45E-09	1.26E-07	16
SARS CoV Infections	neuron	3.90E-08	1.91E-06	59
Activation of Gene Expression by SREBF SREBP	neuron	6.37E-08	2.99E-06	14
SARS CoV 2 Infection	neuron	7.20E-08	3.25E-06	43
Cytoprotection by HMOX1	neuron	1.13E-07	4.88E-06	16
Cholesterol Biosynthesis	neuron	1.57E-07	6.56E-06	11
TP53 Regulates Metabolic Genes	neuron	1.73E-07	6.94E-06	19
Regulation of Cholesterol Biosynthesis by SREBP SREBF	neuron	4.25E-07	1.65E-05	15
Cellular Response to Chemical Stress	neuron	1.15E-06	4.32E-05	30
ER to Golgi Anterograde Transport	neuron	4.66E-06	1.69E-04	25
Transport to the Golgi and Subsequent Modification	neuron	4.96E-06	1.75E-04	28
Transcriptional Regulation by TP53	neuron	6.44E-06	2.20E-04	43
HIV Infection	neuron	1.29E-05	4.28E-04	30
Bacterial Infection Pathways	neuron	1.35E-05	4.34E-04	15
Host Interactions of HIV Factors	neuron	1.78E-05	5.56E-04	20
mRNA Splicing	neuron	2.01E-05	6.13E-04	29

Eph Ephrin Signaling	neuron	2.37E-05	7.04E-04	17
COPI Mediated Anterograde Transport	neuron	2.64E-05	7.64E-04	18
Asparagine N Linked Glycosylation	neuron	4.00E-05	1.13E-03	37
Processing of Capped Intron Containing pre mRNA	neuron	6.29E-05	1.73E-03	34
Regulation of MECP2 Expression and Activity	neuron	6.92E-05	1.86E-03	9
Beta Catenin Independent WNT Signaling	neuron	1.03E-04	2.71E-03	20
Infection with Mycobacterium Tuberculosis	neuron	1.17E-04	3.01E-03	8
CaMK IV Mediated Phosphorylation of CREB	neuron	1.41E-04	3.52E-03	5
FLT3 Signaling in Disease	neuron	1.56E-04	3.82E-03	8
Response of MTB to Phagocytosis	neuron	2.63E-04	6.29E-03	7
Metabolism of Steroids	neuron	2.86E-04	6.71E-03	21
Protein Methylation	neuron	2.96E-04	6.82E-03	6
Golgi to ER Retrograde Transport	neuron	3.32E-04	7.49E-03	19

Tab. S7. Reactome pathway enrichment analysis for AD dataset. Reactome pathway database enrichment analysis performed on all significantly differentially expressed genes (DEGs; FDR < 0.05, irrespective of directionality) identified in the AD organoid dataset. The analysis was conducted using the *ReactomePA* package with hypergeometric test and multiple testing correction. Columns represent: Reactome pathway ID, cell type, nominal p-value, FDR, and total gene count. Pathways with FDR < 0.05 and a minimum of 5 genes are included, with a maximum of the top 50 pathways per cell type reported.

Pathway	Cell Type	p-value	FDR	Count
SRP Dependent Cotranslational Protein Targeting to Membrane	astrocyte	1.20E-44	1.27E-41	59
Eukaryotic Translation Elongation	astrocyte	6.27E-44	3.33E-41	54
Response of EIF2AK4 GCN2 to Amino Acid Deficiency	astrocyte	2.43E-41	8.60E-39	54
Cellular Response to Starvation	astrocyte	3.73E-40	9.91E-38	64
Regulation of Expression of SLITs and ROBOs	astrocyte	1.52E-39	3.24E-37	64
Signaling by ROBO Receptors	astrocyte	1.14E-36	2.02E-34	69
Translation	astrocyte	6.62E-36	1.01E-33	81
Influenza Infection	astrocyte	1.44E-35	1.91E-33	60
Eukaryotic Translation Initiation	astrocyte	2.34E-35	2.76E-33	53
Selenoamino Acid Metabolism	astrocyte	1.21E-34	1.28E-32	52
Nonsense Mediated Decay NMD	astrocyte	6.22E-34	6.01E-32	51
rRNA Processing	astrocyte	1.52E-27	1.35E-25	59

Metabolism of Amino Acids and Derivatives	astrocyte	6.98E-24	5.71E-22	73
SARS CoV 1 Modulates Host Translation Machinery	astrocyte	6.35E-22	4.82E-20	24
SARS CoV 1 Host Interactions	astrocyte	5.01E-20	3.55E-18	35
SARS CoV 2 Modulates Host Translation Machinery	astrocyte	1.19E-18	7.88E-17	25
Activation of the mRNA upon Binding of the Cap Binding Complex and eIFs and Subsequent Binding to 43S	astrocyte	1.56E-16	9.73E-15	25
SARS CoV 1 Infection	astrocyte	7.73E-16	4.56E-14	37
Aerobic Respiration and Respiratory Electron Transport	astrocyte	4.69E-14	2.62E-12	47
SARS CoV 2 Host Interactions	astrocyte	1.08E-12	5.73E-11	40
SARS CoV 2 Infection	astrocyte	6.97E-11	3.53E-09	47
Respiratory Electron Transport	astrocyte	3.92E-10	1.89E-08	30
SARS CoV Infections	astrocyte	8.40E-10	3.88E-08	60
Complex I Biogenesis	astrocyte	2.13E-08	9.42E-07	17
Antigen Processing Cross Presentation	astrocyte	5.52E-08	2.35E-06	21
SCF SKP2 Mediated Degradation of p27 p21	astrocyte	2.84E-07	1.16E-05	14
Apoptosis	astrocyte	3.20E-07	1.26E-05	27
The Role of GTSE1 in G2 M Progression after G2 Checkpoint	astrocyte	3.58E-07	1.32E-05	16
Host Interactions of HIV Factors	astrocyte	3.61E-07	1.32E-05	22
HSF1 Activation	astrocyte	3.79E-07	1.34E-05	11
mRNA Splicing	astrocyte	5.02E-07	1.72E-05	31
Programmed Cell Death	astrocyte	1.13E-06	3.76E-05	29
Vif Mediated Degradation of APOBEC3G	astrocyte	1.20E-06	3.79E-05	12
Attenuation Phase	astrocyte	1.21E-06	3.79E-05	10
Dectin 1 Mediated Noncanonical NF kB Signaling	astrocyte	1.49E-06	4.53E-05	13
AUF1 hnRNP D0 Binds and Destabilizes mRNA	astrocyte	1.60E-06	4.69E-05	12
Hsp90 Chaperone Cycle for Steroid Hormone Receptors SHR in the Presence of Ligand	astrocyte	1.63E-06	4.69E-05	14
HIV Infection	astrocyte	2.79E-06	7.80E-05	30
Hedgehog Ligand Biogenesis	astrocyte	3.10E-06	8.45E-05	13
Complex III Assembly	astrocyte	3.89E-06	1.03E-04	9
Hh Mutants Abrogate Ligand Secretion	astrocyte	4.59E-06	1.19E-04	12

Cellular Response to Hypoxia	astrocyte	4.78E-06	1.21E-04	14
Ubiquitin Dependent Degradation of Cyclin D	astrocyte	5.02E-06	1.24E-04	11
G1 S DNA Damage Checkpoints	astrocyte	6.08E-06	1.47E-04	13
Degradation of Beta Catenin by the Destruction Complex	astrocyte	6.37E-06	1.51E-04	15
Regulation of Apoptosis	astrocyte	6.58E-06	1.52E-04	11
Neutrophil Degranulation	astrocyte	7.21E-06	1.61E-04	50
Defective CFTR Causes Cystic Fibrosis	astrocyte	7.43E-06	1.61E-04	12
Degradation of CRY and PER Proteins	astrocyte	7.43E-06	1.61E-04	12
Regulation of mRNA Stability by Proteins that Bind AU Rich Elements	astrocyte	9.08E-06	1.93E-04	15
Eukaryotic Translation Elongation	neuron	8.84E-30	7.42E-27	45
Influenza Infection	neuron	1.33E-29	7.42E-27	57
Regulation of Expression of SLITs and ROBOs	neuron	2.61E-28	9.74E-26	56
Signaling by ROBO Receptors	neuron	6.78E-28	1.90E-25	63
Eukaryotic Translation Initiation	neuron	2.84E-26	6.36E-24	47
Response of EIF2AK4 GCN2 to Amino Acid Deficiency	neuron	1.11E-25	2.06E-23	43
SRP Dependent Cotranslational Protein Targeting to Membrane	neuron	1.46E-25	2.33E-23	45
Translation	neuron	4.29E-25	6.01E-23	72
Cellular Response to Starvation	neuron	2.99E-23	3.72E-21	50
Nonsense Mediated Decay NMD	neuron	4.97E-22	5.56E-20	42
Selenoamino Acid Metabolism	neuron	9.38E-21	9.54E-19	41
Aerobic Respiration and Respiratory Electron Transport	neuron	1.86E-16	1.73E-14	54
rRNA Processing	neuron	2.53E-16	2.18E-14	48
mRNA Splicing	neuron	1.58E-15	1.26E-13	48
SARS CoV 1 Modulates Host Translation Machinery	neuron	5.25E-14	3.68E-12	19
SARS CoV 2 Modulates Host Translation Machinery	neuron	5.26E-14	3.68E-12	22
Metabolism of Amino Acids and Derivatives	neuron	1.17E-13	7.68E-12	61
Activation of the mRNA upon Binding of the Cap Binding Complex and eIFs and Subsequent Binding to 43S	neuron	2.95E-13	1.83E-11	23
HIV Infection	neuron	4.29E-13	2.52E-11	45
Processing of Capped Intron Containing pre mRNA	neuron	1.02E-11	5.69E-10	50

Respiratory Electron Transport	neuron	1.83E-11	9.75E-10	34
SARS CoV 1 Host Interactions	neuron	2.19E-11	1.11E-09	27
SARS CoV 1 Infection	neuron	1.24E-10	6.05E-09	32
AUF1 hnRNP D0 Binds and Destabilizes mRNA	neuron	1.38E-10	6.45E-09	17
Degradation of CRY and PER Proteins	neuron	1.78E-10	7.98E-09	18
Host Interactions of HIV Factors	neuron	4.30E-10	1.85E-08	28
The Role of GTSE1 in G2 M Progression after G2 Checkpoint	neuron	1.40E-09	5.65E-08	20
FBXL7 Down Regulates AURKA During Mitotic Entry and in Early Mitosis	neuron	1.41E-09	5.65E-08	16
MAPK6 MAPK4 Signaling	neuron	1.65E-09	6.37E-08	22
Cellular Response to Hypoxia	neuron	2.93E-09	1.06E-07	19
Complex I Biogenesis	neuron	2.93E-09	1.06E-07	19
Negative Regulation of NOTCH4 Signaling	neuron	3.15E-09	1.10E-07	16
mRNA Splicing Minor Pathway	neuron	3.35E-09	1.13E-07	17
Regulation of Apoptosis	neuron	6.00E-09	1.92E-07	15
Hedgehog Off State	neuron	6.01E-09	1.92E-07	24
Hh Mutants Abrogate Ligand Secretion	neuron	6.67E-09	2.07E-07	16
Mitotic G2 G2 M Phases	neuron	6.88E-09	2.08E-07	35
Degradation of Beta Catenin by the Destruction Complex	neuron	7.54E-09	2.18E-07	20
PCP CE Pathway	neuron	7.58E-09	2.18E-07	21
Vif Mediated Degradation of APOBEC3G	neuron	8.93E-09	2.50E-07	15
Mitotic Metaphase and Anaphase	neuron	9.30E-09	2.54E-07	38
Degradation of GLI1 by the Proteasome	neuron	9.53E-09	2.54E-07	16
Separation of Sister Chromatids	neuron	1.06E-08	2.75E-07	33
SCF Beta TRCP Mediated Degradation of EMI1	neuron	1.31E-08	3.33E-07	15
Defective CFTR Causes Cystic Fibrosis	neuron	1.35E-08	3.35E-07	16
Regulation of RAS by GAPs	neuron	1.73E-08	4.21E-07	17
Dectin 1 Mediated Noncanonical NF kB Signaling	neuron	1.89E-08	4.50E-07	16
Mitochondrial Protein Degradation	neuron	2.24E-08	5.22E-07	22
Degradation of DVL	neuron	2.72E-08	6.21E-07	15

Ubiquitin Dependent Degradation of Cyclin D	neuron	3.73E-08	8.35E-07	14

Tab. S8. Reactome pathway enrichment analysis for PD dataset. Reactome pathway database enrichment analysis performed on all significantly differentially expressed genes (DEGs; FDR < 0.05, irrespective of directionality) identified in the PD dataset using methodology identical to Table S7. Results highlight biological processes and molecular pathways altered in PD organoids compared to controls. A maximum of top 50 pathways per cell type are shown.

Pathway	Cell Type	p-value	FDR	Count
Cytoplasmic Ribosomal Proteins	astrocyte	1.80E-103	8.64E-101	76
Cholesterol Biosynthesis Pathway	astrocyte	3.47E-12	8.31E-10	10
Cholesterol Metabolism with Bloch and Kandutschrussell Pathways	astrocyte	1.25E-11	1.99E-09	15
Cholesterol Synthesis Disorders	astrocyte	4.63E-11	5.54E-09	10
Enterocyte Cholesterol Metabolism	astrocyte	8.36E-10	8.01E-08	12
Mevalonate Arm of Cholesterol Biosynthesis Pathway	astrocyte	5.80E-08	4.63E-06	7
Cholesterol Metabolism	astrocyte	7.25E-08	4.96E-06	14
Sterol Regulatory Elementbinding Proteins SREBP Signaling	astrocyte	3.03E-06	1.81E-04	12
Ferroptosis	astrocyte	6.65E-06	3.54E-04	11
VEGFA/VEGFR2 Signaling	astrocyte	8.56E-05	4.10E-03	30
Cholesterol Biosynthesis Pathway in Hepatocytes	astrocyte	2.87E-04	1.25E-02	12
Apoptosis-Related Network Due to Altered NOTCH3 in Ovarian Cancer	astrocyte	3.14E-04	1.25E-02	8
Pathogenic Escherichia Coli Infection	astrocyte	4.07E-04	1.50E-02	8
Glycolysis and Gluconeogenesis	astrocyte	6.18E-04	2.11E-02	7
Photodynamic Therapy-Induced NFE2L2 NRF2 Survival Signaling	astrocyte	7.85E-04	2.51E-02	5
Translation Factors	astrocyte	1.18E-03	3.33E-02	7
Photodynamic Therapy-Induced Unfolded Protein Response	astrocyte	1.69E-03	4.50E-02	5
Clear Cell Renal Cell Carcinoma Pathways	astrocyte	2.05E-03	4.92E-02	9
Cytoplasmic Ribosomal Proteins	neuron	1.52E-65	8.98E-63	69
Mitochondrial Complex IV Assembly	neuron	1.71E-10	5.06E-08	15
VEGFA/VEGFR2 Signaling	neuron	3.40E-10	6.70E-08	64
Nonalcoholic Fatty Liver Disease	neuron	2.21E-09	3.27E-07	32
Electron Transport Chain OXPHOS System in Mitochondria	neuron	3.03E-09	3.59E-07	24
Cholesterol Synthesis Disorders	neuron	3.73E-08	3.68E-06	10
Cholesterol Biosynthesis Pathway	neuron	7.46E-08	6.31E-06	9

Cholesterol Metabolism with Bloch and Kandutschrussell Pathways	neuron	1.59E-07	1.18E-05	15
Mevalonate Arm of Cholesterol Biosynthesis Pathway	neuron	3.15E-07	2.07E-05	8
Cori Cycle	neuron	4.70E-06	2.78E-04	8
Brain-Derived Neurotrophic Factor BDNF Signaling	neuron	6.29E-06	3.39E-04	25
Enterocyte Cholesterol Metabolism	neuron	1.18E-05	5.81E-04	11
Common Pathways Underlying Drug Addiction	neuron	4.58E-05	2.09E-03	11
mRNA Processing	neuron	6.42E-05	2.71E-03	21
Aerobic Glycolysis Augmented	neuron	8.98E-05	3.55E-03	6
Omega-9 Fatty Acid Synthesis	neuron	1.49E-04	5.49E-03	6
Pathogenic Escherichia Coli Infection	neuron	1.85E-04	6.43E-03	12
Cholesterol Metabolism	neuron	2.03E-04	6.67E-03	14
Hippocampal Synaptogenesis and Neurogenesis	neuron	2.39E-04	7.44E-03	8
Splicing Factor NOVA Regulated Synaptic Proteins	neuron	2.98E-04	8.81E-03	10
16p11.2 Copy Number Variation Syndrome 520kb	neuron	5.38E-04	1.45E-02	11
Glycolysis and Gluconeogenesis	neuron	5.40E-04	1.45E-02	10
Amyotrophic Lateral Sclerosis ALS	neuron	6.20E-04	1.48E-02	9
5q35 Copy Number Variation	neuron	6.23E-04	1.48E-02	17
Sterol Regulatory Elementbinding Proteins SREBP Signaling	neuron	6.25E-04	1.48E-02	13
Prion Disease Pathway	neuron	8.45E-04	1.91E-02	8
Fragile X Syndrome	neuron	8.69E-04	1.91E-02	18
Alzheimer's Disease	neuron	9.13E-04	1.93E-02	31
Lactate Shuttle in Glial Cells	neuron	9.90E-04	2.02E-02	5
HDAC6 Interactions in the Central Nervous System	neuron	1.05E-03	2.08E-02	17
Effect of Omega-3 PUFA on Huntington's Disease Pathways	neuron	2.06E-03	3.83E-02	17
Mitochondrial Complex III Assembly	neuron	2.07E-03	3.83E-02	5
G13 Signaling	neuron	2.77E-03	4.83E-02	8
Photodynamic Therapy-Induced HIF1 Survival Signaling	neuron	2.77E-03	4.83E-02	8

Tab. S9. Wikipathways pathway enrichment analysis for AD dataset. Wikipathways enrichment analysis performed on all significantly differentially expressed genes (DEGs; FDR < 0.05, irrespective of directionality) identified in the AD organoid dataset. The analysis was conducted using the clusterProfiler package with hypergeometric test and multiple testing correction. Columns represent: Wikipathways pathway ID, cell type, nominal p-value, FDR, and total gene count. Pathways with FDR < 0.05 and minimum 5 genes are included. A maximum of top 50 pathways per cell type are shown.

Pathway	Cell Type	p-value	FDR	Count
Cytoplasmic Ribosomal Proteins	astrocyte	8.65E-41	4.91E-38	51

Electron Transport Chain OXPHOS System in Mitochondria	astrocyte	1.08E-13	3.07E-11	28
Oxidative Phosphorylation	astrocyte	2.67E-10	5.06E-08	18
VEGFA/VEGFR2 Signaling	astrocyte	1.70E-08	2.42E-06	55
Nonalcoholic Fatty Liver Disease	astrocyte	4.49E-07	5.10E-05	26
Proteasome Degradation	astrocyte	1.65E-06	1.56E-04	15
Cholesterol Synthesis Disorders	astrocyte	3.10E-06	2.35E-04	8
miR-Targeted Genes in Squamous Cell	astrocyte	3.31E-06	2.35E-04	25
Cholesterol Biosynthesis Pathway	astrocyte	8.92E-06	5.04E-04	7
Mitochondrial Complex III Assembly	astrocyte	8.92E-06	5.04E-04	7
mRNA Processing	astrocyte	9.76E-06	5.04E-04	21
Mitochondrial Complex I Assembly Model OXPHOS System	astrocyte	1.99E-05	9.41E-04	12
Pathogenic Escherichia Coli Infection	astrocyte	5.51E-05	2.30E-03	12
Cholesterol Metabolism with Bloch and Kandutschrussell Pathways	astrocyte	5.66E-05	2.30E-03	11
16p11.2 Copy Number Variation Syndrome 520kb	astrocyte	1.81E-04	6.41E-03	11
Apoptosis-Related Network Due to Altered NOTCH3 in Ovarian Cancer	astrocyte	1.81E-04	6.41E-03	11
Primary Focal Segmental Glomerulosclerosis FSGS	astrocyte	2.12E-04	7.10E-03	13
miR-Targeted Genes in Leukocytes	astrocyte	2.43E-04	7.28E-03	21
Photodynamic Therapy-Induced HIF1 Survival Signaling	astrocyte	2.44E-04	7.28E-03	9
Exercise-Induced Circadian Regulation	astrocyte	3.42E-04	9.72E-03	10
Metabolic Reprogramming in Colon Cancer	astrocyte	5.42E-04	1.43E-02	9
Lactate Shuttle in Glial Cells	astrocyte	5.54E-04	1.43E-02	5
Photodynamic Therapy-Induced Unfolded Protein Response	astrocyte	6.70E-04	1.62E-02	7
Metabolic Epileptic Disorders	astrocyte	6.83E-04	1.62E-02	14
Enterocyte Cholesterol Metabolism	astrocyte	8.51E-04	1.93E-02	8
Glycolysis and Gluconeogenesis	astrocyte	9.23E-04	2.02E-02	9
Ferroptosis	astrocyte	9.90E-04	2.08E-02	11
Physicochemical Features and Toxicity-Associated Pathways	astrocyte	1.29E-03	2.61E-02	11
Cell Cycle	astrocyte	1.44E-03	2.82E-02	16
TROP2 Regulatory Signaling	astrocyte	1.74E-03	3.22E-02	9
Parkin-Ubiquitin Proteasomal System Pathway	astrocyte	2.36E-03	4.20E-02	11
Cytoplasmic Ribosomal Proteins	neuron	4.82E-24	2.74E-21	40
Electron Transport Chain OXPHOS System in Mitochondria	neuron	3.47E-17	9.86E-15	34
Oxidative Phosphorylation	neuron	4.25E-15	8.05E-13	24
mRNA Processing	neuron	1.80E-10	2.56E-08	31
Nonalcoholic Fatty Liver Disease	neuron	3.10E-10	3.52E-08	34
Mitochondrial Complex I Assembly Model OXPHOS System	neuron	1.72E-09	1.63E-07	18
Glycolysis and Gluconeogenesis	neuron	8.96E-07	7.27E-05	14
Alzheimer's Disease	neuron	2.27E-06	1.55E-04	39

Proteasome Degradation	neuron	2.45E-06	1.55E-04	16
Translation Factors	neuron	1.97E-05	1.12E-03	13
Parkin-Ubiquitin Proteasomal System Pathway	neuron	6.60E-05	3.41E-03	15
TCA Cycle AKA Krebs or Citric Acid Cycle	neuron	1.06E-04	4.94E-03	7
Metabolic Epileptic Disorders	neuron	1.13E-04	4.94E-03	17
HDAC6 Interactions in the Central Nervous System	neuron	1.97E-04	7.99E-03	19
Alzheimer's Disease and miRNA Effects	neuron	3.62E-04	1.37E-02	39
Fragile X Syndrome	neuron	4.85E-04	1.72E-02	19
RALA Downstream Regulated Genes	neuron	7.55E-04	2.52E-02	5
Ferroptosis	neuron	1.09E-03	3.43E-02	12
Aerobic Glycolysis Augmented	neuron	1.16E-03	3.46E-02	5
VEGFA/VEGFR2 Signaling	neuron	1.38E-03	3.93E-02	46
7q11.23 Copy Number Variation Syndrome	neuron	1.65E-03	4.45E-02	16

Tab. S10. WikiPathways pathway enrichment analysis for PD dataset. Wikipathways enrichment analysis performed on all significantly differentially expressed genes (DEGs; FDR < 0.05, irrespective of directionality) for PD dataset using methodology identical to Table S9. Results highlight biological processes and molecular pathways altered in PD organoids compared to controls. A maximum of top 50 pathways per cell type are shown.

Pathway	Cell Type	p-value	FDR	Count
Cytoplasmic translation	astrocyte	2.92E-96	1.10E-92	81
Ribosome biogenesis	astrocyte	6.99E-20	1.32E-16	39
Ribonucleoprotein complex biogenesis	astrocyte	2.79E-18	3.51E-15	46
Ribosomal small subunit biogenesis	astrocyte	4.29E-18	4.05E-15	23
Ribosome assembly	astrocyte	8.53E-13	6.44E-10	15
Sterol biosynthetic process	astrocyte	4.23E-11	2.66E-08	14
rRNA processing	astrocyte	1.24E-10	5.47E-08	23
Cholesterol biosynthetic process	astrocyte	1.30E-10	5.47E-08	13
Secondary alcohol biosynthetic process	astrocyte	1.30E-10	5.47E-08	13
Ribosomal small subunit assembly	astrocyte	8.07E-10	3.05E-07	8
Protein-rna complex assembly	astrocyte	1.69E-09	5.81E-07	22
Ribosomal large subunit biogenesis	astrocyte	2.06E-09	6.47E-07	13
rRNA metabolic process	astrocyte	3.65E-09	9.99E-07	23
Protein-RNA complex organization	astrocyte	3.70E-09	9.99E-07	22
Negative regulation of protein modification by small protein conjugation or removal	astrocyte	2.43E-08	5.98E-06	13
Intrinsic apoptotic signaling pathway	astrocyte	2.53E-08	5.98E-06	24
Negative regulation of protein ubiquitination	astrocyte	4.09E-08	8.68E-06	12
Negative regulation of post-translational protein modification	astrocyte	4.14E-08	8.68E-06	13
Regulation of protein ubiquitination	astrocyte	8.75E-08	1.74E-05	18
Cholesterol metabolic process	astrocyte	1.36E-07	2.56E-05	15

Negative regulation of catalytic activity	astrocyte	3.40E-07	5.84E-05	27
Regulation of protein modification by small protein conjugation or removal	astrocyte	3.61E-07	5.84E-05	19
Secondary alcohol metabolic process	astrocyte	3.65E-07	5.84E-05	15
Regulation of translation	astrocyte	3.71E-07	5.84E-05	27
Sterol metabolic process	astrocyte	4.70E-07	6.92E-05	15
Signal transduction by p53 class mediator	astrocyte	4.95E-07	6.92E-05	16
Regulation of post-translational protein modification	astrocyte	4.95E-07	6.92E-05	19
Steroid biosynthetic process	astrocyte	8.97E-07	1.18E-04	16
Alcohol biosynthetic process	astrocyte	9.05E-07	1.18E-04	14
Regulation of intrinsic apoptotic signaling pathway	astrocyte	1.28E-06	1.61E-04	16
Positive regulation of signal transduction by p53 class mediator	astrocyte	1.55E-06	1.89E-04	7
Non-membrane-bounded organelle assembly	astrocyte	2.81E-06	3.31E-04	24
Regulation of apoptotic signaling pathway	astrocyte	4.57E-06	5.23E-04	23
Negative regulation of protein modification process	astrocyte	4.77E-06	5.30E-04	24
Negative regulation of transferase activity	astrocyte	5.66E-06	6.05E-04	16
Ribosomal large subunit assembly	astrocyte	5.76E-06	6.05E-04	6
Regulation of ubiquitin-protein transferase activity	astrocyte	8.39E-06	8.56E-04	7
Regulation of fibroblast proliferation	astrocyte	1.15E-05	1.14E-03	10
Protein refolding	astrocyte	1.21E-05	1.14E-03	6
Cellular response to gamma radiation	astrocyte	1.21E-05	1.14E-03	6
Chaperone-mediated protein folding	astrocyte	1.69E-05	1.55E-03	9
Isoprenoid biosynthetic process	astrocyte	1.87E-05	1.67E-03	6
Alcohol metabolic process	astrocyte	1.90E-05	1.67E-03	21
Regulation of ubiquitin protein ligase activity	astrocyte	2.05E-05	1.76E-03	5
Negative regulation of apoptotic signaling pathway	astrocyte	3.08E-05	2.59E-03	16
Intrinsic apoptotic signaling pathway in response to dna damage	astrocyte	3.47E-05	2.85E-03	10
Translational initiation	astrocyte	3.63E-05	2.92E-03	11
Intrinsic apoptotic signaling pathway by p53 class mediator	astrocyte	3.84E-05	3.02E-03	9
Organic hydroxy compound biosynthetic process	astrocyte	3.94E-05	3.04E-03	16
Regulation of signal transduction by p53 class mediator	astrocyte	5.68E-05	4.28E-03	10
Cytoplasmic translation	neuron	4.06E-59	2.01E-55	78
Ribonucleoprotein complex biogenesis	neuron	3.92E-12	9.71E-09	63
Ribosome biogenesis	neuron	1.35E-10	2.23E-07	45
Ribosome assembly	neuron	4.99E-10	4.09E-07	18
ATP synthesis coupled electron transport	neuron	5.38E-10	4.09E-07	23
Mitochondrial atp synthesis coupled electron transport	neuron	5.38E-10	4.09E-07	23
Aerobic electron transport chain	neuron	5.96E-10	4.09E-07	22
Ribosomal small subunit biogenesis	neuron	6.61E-10	4.09E-07	23
Protein-RNA complex organization	neuron	7.84E-10	4.31E-07	37
Protein-RNA complex assembly	neuron	8.87E-10	4.39E-07	36
Oxidative phosphorylation	neuron	2.67E-09	1.20E-06	27
Respiratory electron transport chain	neuron	5.07E-09	2.09E-06	24

Mitochondrial electron transport, cytochrome c to oxygen	neuron	8.30E-09	3.16E-06	11
RNA splicing	neuron	1.23E-08	4.34E-06	54
Neuron migration	neuron	1.95E-08	6.43E-06	29
Ribosomal small subunit assembly	neuron	4.90E-08	1.51E-05	9
Regulation of translation	neuron	5.24E-08	1.52E-05	50
Cognition	neuron	5.54E-08	1.52E-05	40
Axonogenesis	neuron	6.94E-08	1.81E-05	50
Electron transport chain	neuron	1.05E-07	2.61E-05	27
Synapse organization	neuron	1.43E-07	3.36E-05	52
Aerobic respiration	neuron	4.40E-07	9.89E-05	28
Forebrain development	neuron	7.14E-07	1.54E-04	44
Learning or memory	neuron	7.86E-07	1.62E-04	34
RNA splicing, via transesterification reactions with bulged adenosine as nucleophile	neuron	1.38E-06	2.64E-04	38
mRNA splicing, via spliceosome	neuron	1.38E-06	2.64E-04	38
RNA splicing, via transesterification reactions	neuron	1.85E-06	3.38E-04	38
Dense core granule localization	neuron	2.31E-06	4.09E-04	8
Regulation of neuron projection development	neuron	3.25E-06	5.55E-04	46
Cellular respiration	neuron	3.37E-06	5.57E-04	30
Regulation of synapse structure or activity	neuron	4.69E-06	7.11E-04	30
Telencephalon development	neuron	4.80E-06	7.11E-04	32
Dense core granule cytoskeletal transport	neuron	4.95E-06	7.11E-04	6
Dense core granule transport	neuron	4.95E-06	7.11E-04	6
Cytoskeleton-dependent intracellular transport	neuron	5.03E-06	7.11E-04	26
Negative regulation of mrna metabolic process	neuron	6.28E-06	8.64E-04	17
Secretory granule localization	neuron	7.89E-06	1.06E-03	8
Translational initiation	neuron	8.82E-06	1.15E-03	19
Regulation of neurogenesis	neuron	9.29E-06	1.18E-03	40
Memory	neuron	1.12E-05	1.38E-03	19
Ribosomal large subunit biogenesis	neuron	1.15E-05	1.39E-03	14
Axo-dendritic transport	neuron	1.59E-05	1.87E-03	14
Regulation of synapse organization	neuron	2.22E-05	2.56E-03	28
Energy derivation by oxidation of organic compounds	neuron	2.31E-05	2.60E-03	35
Anterograde axonal transport	neuron	2.76E-05	3.04E-03	11
Synapse assembly	neuron	2.97E-05	3.19E-03	25
Dendrite development	neuron	3.26E-05	3.43E-03	27
Transport along microtubule	neuron	3.39E-05	3.49E-03	22
Establishment of protein localization to membrane	neuron	3.78E-05	3.82E-03	30
Regulation of rna splicing	neuron	3.90E-05	3.86E-03	23

Tab. S11. GO pathway enrichment analysis for AD dataset. GO pathway enrichment analysis performed on all significantly differentially expressed genes (DEGs; FDR < 0.05, irrespective of directionality) identified in the AD organoid dataset. The analysis was conducted using the *clusterProfiler* package with hypergeometric test and multiple testing correction. Columns represent: GO pathway ID, cell type, nominal p-value, FDR, and total

gene count. Pathways with FDR < 0.05 and a minimum of 5 genes are included, with a maximum of the top 50 pathways per cell type reported.

Pathway	Cell Type	p-value	FDR	Count
Cytoplasmic Translation	astrocyte	1.73E-40	8.43E-37	59
Ribonucleoprotein Complex Biogenesis	astrocyte	1.50E-16	3.65E-13	65
Ribosome Biogenesis	astrocyte	1.45E-15	2.35E-12	49
Oxidative Phosphorylation	astrocyte	5.32E-14	6.46E-11	31
Aerobic Respiration	astrocyte	3.08E-13	2.42E-10	35
ATP Synthesis Coupled Electron Transport	astrocyte	3.48E-13	2.42E-10	25
Mitochondrial ATP Synthesis Coupled Electron Transport	astrocyte	3.48E-13	2.42E-10	25
Ribosome Assembly	astrocyte	3.19E-12	1.73E-09	19
Aerobic Electron Transport Chain	astrocyte	3.19E-12	1.73E-09	23
Respiratory Electron Transport Chain	astrocyte	4.66E-12	2.27E-09	26
Cellular Respiration	astrocyte	7.68E-12	3.39E-09	37
Protein-RNA Complex Organization	astrocyte	2.93E-11	1.19E-08	36
ATP Biosynthetic Process	astrocyte	1.11E-10	4.14E-08	22
Purine Ribonucleoside Triphosphate Biosynthetic Process	astrocyte	1.48E-10	4.75E-08	23
Protein-RNA Complex Assembly	astrocyte	1.59E-10	4.75E-08	34
Proton Motive Force-Driven ATP Synthesis	astrocyte	1.65E-10	4.75E-08	19
Ribosomal Small Subunit Biogenesis	astrocyte	1.66E-10	4.75E-08	22
Purine Nucleoside Triphosphate Biosynthetic Process	astrocyte	1.79E-10	4.82E-08	23
Protein Folding	astrocyte	2.31E-10	5.90E-08	33
Nucleoside Triphosphate Biosynthetic Process	astrocyte	3.06E-10	7.43E-08	24
Ribonucleoside Triphosphate Biosynthetic Process	astrocyte	4.43E-10	1.03E-07	23
Electron Transport Chain	astrocyte	7.12E-10	1.57E-07	28
Proton Motive Force-Driven Mitochondrial ATP Synthesis	astrocyte	1.18E-09	2.50E-07	17
Energy Derivation by Oxidation of Organic Compounds	astrocyte	2.12E-09	4.29E-07	40
Regulation of Intrinsic Apoptotic Signaling Pathway	astrocyte	4.79E-09	9.31E-07	28
Ribosomal Large Subunit Biogenesis	astrocyte	6.14E-09	1.15E-06	17
Mitochondrial Electron Transport, NADH to Ubiquinone	astrocyte	1.15E-08	2.06E-06	14
Purine Nucleoside Triphosphate Metabolic Process	astrocyte	1.33E-08	2.31E-06	32
Regulation of Apoptotic Signaling Pathway	astrocyte	2.18E-08	3.66E-06	42
Purine Ribonucleoside Triphosphate Metabolic Process	astrocyte	2.67E-08	4.33E-06	31
Purine Ribonucleotide Biosynthetic Process	astrocyte	4.93E-08	7.73E-06	28
Ribonucleoside Triphosphate Metabolic Process	astrocyte	5.18E-08	7.77E-06	31
Chaperone-Mediated Protein Folding	astrocyte	5.27E-08	7.77E-06	16
Nucleoside Triphosphate Metabolic Process	astrocyte	5.90E-08	8.37E-06	32
Oligodendrocyte Differentiation	astrocyte	6.02E-08	8.37E-06	19
NADH Dehydrogenase Complex Assembly	astrocyte	1.09E-07	1.43E-05	14

Mitochondrial Respiratory Chain Complex I Assembly	astrocyte	1.09E-07	1.43E-05	14
ATP Metabolic Process	astrocyte	1.21E-07	1.55E-05	28
Regulation of Protein Catabolic Process	astrocyte	1.34E-07	1.67E-05	38
Intrinsic Apoptotic Signaling Pathway	astrocyte	1.44E-07	1.74E-05	35
Mitochondrial Respiratory Chain Complex Assembly	astrocyte	1.47E-07	1.74E-05	18
rRNA Processing	astrocyte	1.77E-07	2.05E-05	28
Ribonucleotide Biosynthetic Process	astrocyte	2.13E-07	2.41E-05	28
Response to Unfolded Protein	astrocyte	2.29E-07	2.53E-05	21
Ribosomal Large Subunit Assembly	astrocyte	2.47E-07	2.67E-05	9
Purine Nucleotide Biosynthetic Process	astrocyte	2.71E-07	2.87E-05	30
Response to Topologically Incorrect Protein	astrocyte	2.92E-07	3.02E-05	22
Ribose Phosphate Biosynthetic Process	astrocyte	4.03E-07	4.08E-05	28
Stem Cell Differentiation	astrocyte	4.48E-07	4.44E-05	29
Purine-Containing Compound Biosynthetic Process	astrocyte	5.28E-07	5.05E-05	30
Cytoplasmic Translation	neuron	3.53E-27	1.74E-23	50
Aerobic Respiration	neuron	3.79E-19	8.08E-16	46
Proton Motive Force-Driven ATP Synthesis	neuron	4.93E-19	8.08E-16	29
Ribonucleoprotein Complex Biogenesis	neuron	1.68E-18	2.07E-15	75
ATP Biosynthetic Process	neuron	4.68E-18	4.61E-15	32
Oxidative Phosphorylation	neuron	1.08E-17	8.87E-15	38
Cellular Respiration	neuron	1.60E-17	1.13E-14	49
Proton Motive Force-Driven Mitochondrial ATP Synthesis	neuron	2.03E-17	1.25E-14	26
ATP Metabolic Process	neuron	2.58E-17	1.41E-14	46
RNA Splicing	neuron	5.44E-17	2.68E-14	71
Nucleoside Triphosphate Metabolic Process	neuron	1.11E-16	4.95E-14	50
Purine Ribonucleoside Triphosphate Biosynthetic Process	neuron	1.40E-16	5.76E-14	32
Purine Nucleoside Triphosphate Biosynthetic Process	neuron	1.87E-16	6.69E-14	32
Purine Nucleoside Triphosphate Metabolic Process	neuron	1.90E-16	6.69E-14	48
Purine Ribonucleoside Triphosphate Metabolic Process	neuron	3.45E-16	1.13E-13	47
ATP Synthesis Coupled Electron Transport	neuron	5.20E-16	1.50E-13	30
Mitochondrial ATP Synthesis Coupled Electron Transport	neuron	5.20E-16	1.50E-13	30
Ribonucleoside Triphosphate Biosynthetic Process	neuron	7.43E-16	2.03E-13	32
Nucleoside Triphosphate Biosynthetic Process	neuron	8.10E-16	2.07E-13	33
RNA Splicing, via Transesterification Reactions	neuron	8.42E-16	2.07E-13	56
Ribonucleoside Triphosphate Metabolic Process	neuron	1.09E-15	2.54E-13	47
RNA Splicing, via Transesterification Reactions with Bulged Adenosine as Nucleophile	neuron	2.02E-15	4.33E-13	55
mRNA Splicing, via Spliceosome	neuron	2.02E-15	4.33E-13	55
Energy Derivation by Oxidation of Organic Compounds	neuron	2.30E-15	4.73E-13	55
Respiratory Electron Transport Chain	neuron	1.93E-14	3.80E-12	31
Aerobic Electron Transport Chain	neuron	2.89E-14	5.48E-12	27

Ribosome Biogenesis	neuron	5.04E-14	9.18E-12	51
Protein-RNA Complex Organization	neuron	1.65E-13	2.82E-11	43
Protein-RNA Complex Assembly	neuron	1.66E-13	2.82E-11	42
Ribose Phosphate Metabolic Process	neuron	1.90E-13	3.11E-11	64
Ribose Phosphate Biosynthetic Process	neuron	6.39E-13	1.01E-10	41
Mitochondrial Electron Transport, NADH to Ubiquinone	neuron	1.03E-12	1.58E-10	19
Ribonucleotide Metabolic Process	neuron	1.60E-12	2.38E-10	61
Purine Ribonucleotide Metabolic Process	neuron	2.40E-12	3.48E-10	59
Electron Transport Chain	neuron	3.44E-12	4.84E-10	34
Purine Nucleotide Biosynthetic Process	neuron	3.71E-12	5.07E-10	42
Ribonucleotide Biosynthetic Process	neuron	4.41E-12	5.86E-10	39
Nucleotide Biosynthetic Process	neuron	6.58E-12	8.52E-10	45
Nucleoside Phosphate Biosynthetic Process	neuron	8.35E-12	1.05E-09	45
Purine Ribonucleotide Biosynthetic Process	neuron	9.57E-12	1.18E-09	37
Purine-Containing Compound Biosynthetic Process	neuron	1.04E-11	1.25E-09	42
Ribosome Assembly	neuron	5.46E-11	6.40E-09	19
Regulation of RNA Splicing	neuron	4.01E-10	4.58E-08	32
Transport Along Microtubule	neuron	4.28E-09	4.78E-07	29
Apoptotic Mitochondrial Changes	neuron	7.54E-09	8.24E-07	22
Cytoskeleton-Dependent Intracellular Transport	neuron	1.01E-08	1.08E-06	31
Regulation of Mitochondrion Organization	neuron	1.29E-08	1.35E-06	26
Mitochondrial Respiratory Chain Complex Assembly	neuron	1.34E-08	1.37E-06	21
Regulation of Translational Initiation	neuron	1.53E-08	1.53E-06	19
Axo-Dendritic Transport	neuron	1.69E-08	1.61E-06	18

Tab. S12. GO pathway enrichment analysis for PD dataset. GO pathway enrichment analysis performed on all significantly differentially expressed genes (DEGs; FDR < 0.05, irrespective of directionality) identified in the PD organoid dataset. The analysis was conducted using the *clusterProfiler* package with hypergeometric test and multiple testing correction. Columns represent: GO pathway ID, cell type, nominal p-value, FDR, and total gene count. Pathways with FDR < 0.05 and a minimum of 5 genes are included, with a maximum of the top 50 pathways per cell type reported. Results highlight biological processes and molecular pathways altered in PD organoids compared to controls.

Pathway	Cell Type	p-value	FDR	Trend	Count	Source
Intrinsic Apoptotic Signaling Pathway	astrocyte	4.34E-07	5.87E-04	neutral	8	GO
Regulation of Intrinsic Apoptotic Signaling Pathway	astrocyte	3.72E-06	2.51E-03	neutral	6	GO
KEGG Medicus Pathogen EBV EBNA1 to p53 Mediated Transcription	astrocyte	1.20E-05	2.28E-04	neutral	3	KEGG

KEGG Medicus Pathogen EBV EBNA3C to p53 Mediated Transcription	astrocyte	1.20E-05	2.28E-04	neutral	3	KEGG
FOXO Mediated Transcription	astrocyte	4.76E-05	7.10E-03	neutral	4	Reactome
Basigin Interactions	astrocyte	5.89E-05	7.10E-03	neutral	3	Reactome
Response of EIF2AK4 GCN2 to Amino Acid Deficiency	astrocyte	2.60E-04	2.09E-02	neutral	4	Reactome
TP53 Regulates Transcription of Cell Cycle Genes	astrocyte	4.49E-04	2.70E-02	neutral	3	Reactome
Cellular Response to Starvation	astrocyte	1.29E-03	4.13E-02	neutral	4	Reactome
TP53 Regulates Metabolic Genes	neuron	3.89E-06	1.29E-03	up	7	Reactome
Transcriptional Regulation by TP53	neuron	5.59E-06	1.29E-03	up	13	Reactome
Translation	neuron	1.25E-04	1.93E-02	up	10	Reactome
Respiratory Electron Transport	neuron	2.01E-04	2.33E-02	up	7	Reactome
The NLRP3 Inflammasome	neuron	2.67E-04	2.47E-02	up	3	Reactome
HIV Transcription Initiation	neuron	5.52E-04	3.87E-02	up	4	Reactome
Inflammasomes	neuron	6.16E-04	3.87E-02	up	3	Reactome
mRNA Splicing Minor Pathway	neuron	7.00E-04	3.87E-02	up	4	Reactome
Eph Ephrin Signaling	neuron	8.85E-04	3.87E-02	up	5	Reactome
Aerobic Respiration and Respiratory Electron Transport	neuron	9.11E-04	3.87E-02	up	8	Reactome
Cellular Response to Chemical Stress	neuron	9.83E-04	3.87E-02	up	7	Reactome
Defective Intrinsic Pathway for Apoptosis	neuron	1.04E-03	3.87E-02	up	3	Reactome
Cytoprotection by HMOX1	neuron	1.15E-03	3.87E-02	up	4	Reactome
Purinergic Signaling in Leishmaniasis Infection	neuron	1.17E-03	3.87E-02	up	3	Reactome
FGFR2 Alternative Splicing	neuron	1.31E-03	4.04E-02	up	3	Reactome
TP53 Regulates Transcription of DNA Repair Genes	neuron	1.57E-03	4.22E-02	up	4	Reactome
mRNA Capping	neuron	1.62E-03	4.22E-02	up	3	Reactome
mRNA Splicing	neuron	1.64E-03	4.22E-02	up	7	Reactome
TP53 Network	astrocyte	4.03E-05	7.62E-03	neutral	3	WikiPathways
Pancreatic Adenocarcinoma Pathway	astrocyte	2.80E-04	2.36E-02	neutral	4	WikiPathways
p53 Transcriptional Gene Network	astrocyte	3.75E-04	2.36E-02	neutral	4	WikiPathways

Tab. S13. Cross-dataset pathway comparison - pathways enriched in shared DEGs. Comparative pathway enrichment analysis performed on all significantly differentially expressed genes (DEGs; FDR < 0.05) that are shared between AD and PD organoid datasets and show concordant directionality (over- or under-expressed in both diseases). The table columns include pathway name, cell types showing enrichment in both datasets, p-values, FDR values, direction of enrichment (up/down/neutral), gene count, and pathway source (GO/KEGG/Reactome/WikiPathways). Only pathways with FDR < 0.05 and Count >= 3 are included and a maximum of top 50 pathways per cell type are shown. This analysis reveals potential conserved pathological mechanisms across neurodegenerative conditions.

Pathway	Cell Type	p-value	FDR	Trend AD	Trend PD	Count	Source
Cytoplasmic Translation	astrocyte	2.19E-69	4.17E-66	up	down	48	GO
Ribosome Biogenesis	astrocyte	9.63E-23	9.17E-20	up	down	27	GO
Ribonucleoprotein Complex Biogenesis	astrocyte	7.64E-20	4.85E-17	up	down	29	GO
Ribosomal Small Subunit Biogenesis	astrocyte	4.68E-18	2.23E-15	up	down	16	GO
Ribosome Assembly	astrocyte	4.99E-15	1.90E-12	up	down	12	GO
rRNA Processing	astrocyte	1.62E-11	5.14E-09	up	down	15	GO
Ribosomal Large Subunit Biogenesis	astrocyte	4.41E-11	1.20E-08	up	down	10	GO
rRNA Metabolic Process	astrocyte	1.84E-10	4.38E-08	up	down	15	GO
Protein-RNA Complex Assembly	astrocyte	3.56E-10	7.53E-08	up	down	14	GO
Protein-RNA Complex Organization	astrocyte	6.16E-10	1.17E-07	up	down	14	GO
Ribosomal Large Subunit Assembly	astrocyte	7.85E-09	1.36E-06	up	down	6	GO
Ribosomal Small Subunit Assembly	astrocyte	8.29E-08	1.32E-05	up	down	5	GO
Non-Membrane-Bounded Organelle Assembly	astrocyte	4.59E-07	6.73E-05	up	down	14	GO
Sterol Biosynthetic Process	astrocyte	4.64E-06	6.00E-04	down	up	6	GO
Regulation of Ubiquitin Protein Ligase Activity	astrocyte	4.73E-06	6.00E-04	up	down	4	GO
Maturation of SSU-rRNA	astrocyte	2.54E-05	3.03E-03	up	down	5	GO
Positive Regulation of Signal Transduction by p53 Class Mediator	astrocyte	3.99E-05	4.27E-03	up	down	4	GO
Cholesterol Biosynthetic Process	astrocyte	4.26E-05	4.27E-03	down	up	5	GO
Secondary Alcohol Biosynthetic Process	astrocyte	4.26E-05	4.27E-03	down	up	5	GO
Regulation of Ubiquitin-Protein Transferase Activity	astrocyte	1.03E-04	9.83E-03	up	down	4	GO
Negative Regulation of Ubiquitin-Protein Transferase Activity	astrocyte	1.13E-04	1.03E-02	up	down	3	GO
Regulation of Translation	astrocyte	1.66E-04	1.39E-02	up	down	11	GO
Embryonic Brain Development	astrocyte	1.67E-04	1.39E-02	down	up	3	GO
Cognition	astrocyte	2.46E-04	1.95E-02	down	up	9	GO
Negative Regulation of Protein Modification by Small Protein Conjugation or Removal	astrocyte	2.94E-04	2.18E-02	neutral	down	5	GO
Regulation of Protein Ubiquitination	astrocyte	3.03E-04	2.18E-02	neutral	down	7	GO
Cholesterol Metabolic Process	astrocyte	3.09E-04	2.18E-02	down	up	6	GO
Erythrocyte Homeostasis	astrocyte	3.46E-04	2.36E-02	up	down	6	GO
Negative Regulation of Post-Translational Protein Modification	astrocyte	3.59E-04	2.36E-02	neutral	down	5	GO
Learning or Memory	astrocyte	4.51E-04	2.84E-02	down	up	8	GO
Secondary Alcohol Metabolic Process	astrocyte	4.62E-04	2.84E-02	down	up	6	GO
Negative Regulation of Catabolic Process	astrocyte	4.80E-04	2.86E-02	neutral	neutral	9	GO

Sterol Metabolic Process	astrocyte	5.12E-04	2.96E-02	down	up	6	GO
Regulation of Signal Transduction by p53 Class Mediator	astrocyte	6.79E-04	3.74E-02	up	down	5	GO
Protein Refolding	astrocyte	6.87E-04	3.74E-02	up	down	3	GO
Isoprenoid Biosynthetic Process	astrocyte	8.50E-04	4.50E-02	down	up	3	GO
Cytoplasmic Translation	neuron	9.65E-40	2.34E-36	up	down	35	GO
Ribonucleoprotein Complex Biogenesis	neuron	1.90E-14	2.30E-11	up	down	27	GO
Ribosome Biogenesis	neuron	4.75E-14	3.85E-11	up	down	22	GO
Ribosome Assembly	neuron	1.57E-13	9.51E-11	up	down	12	GO
Protein-RNA Complex Assembly	neuron	1.87E-11	9.06E-09	neutral	down	17	GO
Protein-RNA Complex Organization	neuron	3.62E-11	1.47E-08	neutral	down	17	GO
Ribosomal Small Subunit Biogenesis	neuron	8.20E-11	2.84E-08	up	down	12	GO
Ribosomal Small Subunit Assembly	neuron	6.18E-09	1.88E-06	up	down	6	GO
rRNA Processing	neuron	5.43E-07	1.35E-04	up	down	12	GO
Non-Membrane-Bounded Organelle Assembly	neuron	5.56E-07	1.35E-04	up	down	16	GO
Ribosomal Large Subunit Assembly	neuron	1.63E-06	3.59E-04	up	down	5	GO
rRNA Metabolic Process	neuron	3.29E-06	6.22E-04	up	down	12	GO
Ribosomal Large Subunit Biogenesis	neuron	3.33E-06	6.22E-04	up	down	7	GO
RNA Splicing	neuron	7.58E-05	1.31E-02	down	neutral	14	GO
Translational Initiation	neuron	9.74E-05	1.58E-02	neutral	down	7	GO
Positive Regulation of Viral Process	neuron	2.21E-04	3.36E-02	down	neutral	5	GO
Translation Initiation	astrocyte	7.08E-57	5.80E-55	up	down	46	KEGG
Translation Initiation	neuron	1.58E-34	2.18E-32	up	down	34	KEGG
Eukaryotic Translation Elongation	astrocyte	2.86E-73	1.71E-70	up	down	47	Reactome
Response of EIF2AK4 GCN2 to Amino Acid Deficiency	astrocyte	7.23E-69	2.16E-66	up	down	46	Reactome
SRP Dependent Cotranslational Protein Targeting to Membrane	astrocyte	2.51E-68	4.99E-66	up	down	47	Reactome
Nonsense Mediated Decay NMD	astrocyte	1.40E-65	2.09E-63	up	down	46	Reactome
Selenoamino Acid Metabolism	astrocyte	3.74E-65	4.46E-63	up	down	46	Reactome
Eukaryotic Translation Initiation	astrocyte	9.77E-65	9.72E-63	up	down	46	Reactome
Influenza Infection	astrocyte	4.60E-62	3.92E-60	up	down	48	Reactome
Cellular Response to Starvation	astrocyte	1.86E-58	1.39E-56	up	down	46	Reactome
Regulation of Expression of SLITs and ROBOs	astrocyte	5.13E-58	3.40E-56	up	down	46	Reactome
rRNA Processing	astrocyte	1.68E-52	1.00E-50	up	down	46	Reactome
Signaling by ROBO Receptors	astrocyte	2.77E-52	1.50E-50	up	down	46	Reactome

Translation	astrocyte	1.17E-47	5.83E-46	up	down	48	Reactome
Metabolism of Amino Acids and Derivatives	astrocyte	3.17E-42	1.45E-40	up	down	47	Reactome
SARS CoV 1 Modulates Host Translation Machinery	astrocyte	4.50E-36	1.92E-34	up	down	22	Reactome
SARS CoV 2 Modulates Host Translation Machinery	astrocyte	6.24E-30	2.48E-28	up	down	21	Reactome
Activation of the mRNA upon Binding of the Cap Binding Complex and eIFs and Subsequent Binding to 43S	astrocyte	4.07E-28	1.52E-26	up	down	21	Reactome
SARS CoV 1 Host Interactions	astrocyte	1.35E-24	4.75E-23	up	down	22	Reactome
SARS CoV 2 Host Interactions	astrocyte	2.66E-22	8.83E-21	up	down	26	Reactome
SARS CoV 1 Infection	astrocyte	9.19E-21	2.89E-19	up	down	22	Reactome
SARS CoV 2 Infection	astrocyte	5.20E-19	1.55E-17	up	down	27	Reactome
SARS CoV Infections	astrocyte	5.26E-15	1.50E-13	up	down	28	Reactome
Cholesterol Biosynthesis	astrocyte	1.81E-07	4.91E-06	down	up	6	Reactome
Activation of Gene Expression by SREBF SREBP	astrocyte	2.85E-06	7.40E-05	down	up	6	Reactome
Regulation of Cholesterol Biosynthesis by SREBP SREBF	astrocyte	1.42E-05	3.54E-04	down	up	6	Reactome
Protein Hydroxylation	astrocyte	3.59E-05	8.57E-04	up	down	4	Reactome
HSF1 Activation	astrocyte	2.15E-04	4.93E-03	up	down	4	Reactome
Metabolism of Steroids	astrocyte	7.31E-04	1.62E-02	down	up	7	Reactome
Aggrephagy	astrocyte	8.41E-04	1.79E-02	neutral	down	4	Reactome
Chaperone Mediated Autophagy	astrocyte	1.19E-03	2.45E-02	up	down	3	Reactome
SARS CoV 2 Activates Modulates Innate and Adaptive Immune Responses	astrocyte	1.39E-03	2.76E-02	up	down	6	Reactome
Hsp90 Chaperone Cycle for Steroid Hormone Receptors SHR in the Presence of Ligand	astrocyte	2.22E-03	4.28E-02	up	down	4	Reactome
Attenuation Phase	astrocyte	2.43E-03	4.54E-02	up	down	3	Reactome
rRNA Modification in the Nucleus and Cytosol	astrocyte	2.68E-03	4.85E-02	up	down	4	Reactome
Eukaryotic Translation Elongation	neuron	5.90E-46	4.42E-43	up	down	36	Reactome
Response of EIF2AK4 GCN2 to Amino Acid Deficiency	neuron	1.22E-42	4.57E-40	up	down	35	Reactome
Influenza Infection	neuron	9.11E-40	2.27E-37	up	down	38	Reactome

SRP Dependent Cotranslational Protein Targeting to Membrane	neuron	3.93E-39	7.36E-37	up	down	34	Reactome
Nonsense Mediated Decay NMD	neuron	1.09E-38	1.64E-36	up	down	34	Reactome
Selenoamino Acid Metabolism	neuron	2.13E-38	2.66E-36	up	down	34	Reactome
Eukaryotic Translation Initiation	neuron	4.08E-38	4.37E-36	up	down	34	Reactome
Regulation of Expression of SLITs and ROBOs	neuron	5.31E-38	4.97E-36	up	down	37	Reactome
Cellular Response to Starvation	neuron	8.22E-37	6.84E-35	up	down	36	Reactome
Signaling by ROBO Receptors	neuron	5.14E-35	3.85E-33	up	down	38	Reactome
rRNA Processing	neuron	4.82E-31	3.28E-29	up	down	35	Reactome
Translation	neuron	9.49E-28	5.92E-26	up	down	37	Reactome
Metabolism of Amino Acids and Derivatives	neuron	5.75E-26	3.31E-24	up	down	38	Reactome
SARS CoV 1 Modulates Host Translation Machinery	neuron	3.78E-18	2.02E-16	up	down	14	Reactome
SARS CoV 2 Modulates Host Translation Machinery	neuron	6.94E-16	3.46E-14	up	down	14	Reactome
Activation of the mRNA upon Binding of the Cap Binding Complex and eIFs and Subsequent Binding to 43S	neuron	8.48E-15	3.97E-13	up	down	14	Reactome
SARS CoV 1 Host Interactions	neuron	3.82E-14	1.68E-12	up	down	16	Reactome
SARS CoV 1 Infection	neuron	1.56E-11	6.48E-10	up	down	16	Reactome
SARS CoV 2 Host Interactions	neuron	4.39E-11	1.73E-09	up	down	18	Reactome
SARS CoV 2 Infection	neuron	4.16E-09	1.56E-07	up	down	19	Reactome
SARS CoV Infections	neuron	1.12E-06	4.01E-05	up	down	20	Reactome
AUF1 hnRNP D0 Binds and Destabilizes mRNA	neuron	1.46E-04	4.98E-03	neutral	down	5	Reactome
Regulation of mRNA Stability by Proteins that Bind AU Rich Elements	neuron	2.72E-04	8.86E-03	neutral	neutral	6	Reactome
Cholesterol Biosynthesis	neuron	2.95E-04	9.18E-03	down	up	4	Reactome
Pyruvate Metabolism	neuron	3.06E-04	9.18E-03	neutral	neutral	5	Reactome
FLT3 Signaling in Disease	neuron	3.41E-04	9.83E-03	neutral	neutral	4	Reactome
Host Interactions of HIV Factors	neuron	5.79E-04	1.61E-02	neutral	neutral	7	Reactome
Selective Autophagy	neuron	7.81E-04	2.09E-02	up	down	6	Reactome

Aerobic Respiration and Respiratory Electron Transport	neuron	9.11E-04	2.24E-02	up	down	10	Reactome
Cellular Response to Hypoxia	neuron	9.16E-04	2.24E-02	up	down	5	Reactome
Signaling by ALK in Cancer	neuron	9.28E-04	2.24E-02	down	up	6	Reactome
The Role of GTSE1 in G2 M Progression after G2 Checkpoint	neuron	1.22E-03	2.81E-02	up	down	5	Reactome
Ubiquitin Dependent Degradation of Cyclin D	neuron	1.24E-03	2.81E-02	neutral	neutral	4	Reactome
Hedgehog Off State	neuron	1.35E-03	2.83E-02	up	down	6	Reactome
HIV Infection	neuron	1.36E-03	2.83E-02	neutral	neutral	9	Reactome
Regulation of Apoptosis	neuron	1.36E-03	2.83E-02	neutral	neutral	4	Reactome
Separation of Sister Chromatids	neuron	1.47E-03	2.87E-02	neutral	down	8	Reactome
Constitutive Signaling by Ligand Responsive EGFR Cancer Variants	neuron	1.48E-03	2.87E-02	neutral	neutral	3	Reactome
Vif Mediated Degradation of APOBEC3G	neuron	1.49E-03	2.87E-02	neutral	neutral	4	Reactome
Degradation of AXIN	neuron	1.64E-03	2.92E-02	neutral	neutral	4	Reactome
FBXL7 Down Regulates AURKA During Mitotic Entry and in Early Mitosis	neuron	1.64E-03	2.92E-02	neutral	neutral	4	Reactome
SCF Beta TRCP Mediated Degradation of EMI1	neuron	1.64E-03	2.92E-02	neutral	neutral	4	Reactome
Degradation of Beta Catenin by the Destruction Complex	neuron	1.80E-03	3.10E-02	neutral	neutral	5	Reactome
Aggrephagy	neuron	1.95E-03	3.10E-02	up	down	4	Reactome
Degradation of DVL	neuron	1.95E-03	3.10E-02	neutral	neutral	4	Reactome
Negative Regulation of NOTCH4 Signaling	neuron	1.95E-03	3.10E-02	neutral	neutral	4	Reactome
Stabilization of p53	neuron	1.95E-03	3.10E-02	neutral	neutral	4	Reactome
Regulation of RUNX3 Expression and Activity	neuron	2.12E-03	3.30E-02	neutral	neutral	4	Reactome
Chaperone Mediated Autophagy	neuron	2.29E-03	3.32E-02	up	down	3	Reactome
Regulation of Signaling by CBL	neuron	2.29E-03	3.32E-02	neutral	neutral	3	Reactome
Cytoplasmic Ribosomal Proteins	astrocyte	1.16E-69	3.23E-67	up	down	46	WikiPathways
Cholesterol Biosynthesis Pathway	astrocyte	9.34E-09	1.30E-06	down	up	6	WikiPathways
Cholesterol Synthesis Disorders	astrocyte	3.37E-08	3.12E-06	down	up	6	WikiPathways
Cholesterol Metabolism with Bloch and Kandutschrussell Pathways	astrocyte	5.15E-08	3.58E-06	down	up	8	WikiPathways

Enterocyte Cholesterol Metabolism	astrocyte	2.99E-06	1.66E-04	down	up	6	WikiPathways
Mevalonate Arm of Cholesterol Biosynthesis Pathway	astrocyte	1.10E-05	5.09E-04	down	up	4	WikiPathways
Sterol Regulatory Elementbinding Proteins SREBP Signaling	astrocyte	1.58E-04	5.95E-03	down	up	6	WikiPathways
Cholesterol Metabolism	astrocyte	1.71E-04	5.95E-03	down	up	6	WikiPathways
Cytoplasmic Ribosomal Proteins	neuron	5.62E-42	1.57E-39	up	down	34	WikiPathways
Cholesterol Biosynthesis Pathway	neuron	4.10E-05	5.72E-03	down	up	4	WikiPathways
Cholesterol Synthesis Disorders	neuron	8.90E-05	8.28E-03	down	up	4	WikiPathways

Tab. S14. Cross-dataset pathway comparison - pathways enriched in contrasting DEGs. Comparative pathway enrichment analysis performed on all significantly differentially expressed genes (DEGs; FDR < 0.05) that are shared between AD and PD organoid datasets but show opposite directionality (over-expressed in one disease and under-expressed in the other). Table format follows Table S7 (FDR<5, Count >=3) but focuses on pathways with an overrepresentation of contrasting DEGs. These results highlight potential condition-specific molecular mechanisms.

Condition	Cell Type	Ligand-Receptor Pairs	Interaction Weight	AUPR Classic	AUPR Corrected
			Median / Mean / Min / Max	Mean / Median / SD	Mean / Median / SD
PD	Astrocyte	58	0.600 / 0.664 / 0.101 / 1.672	0.289 / 0.290 / 0.009	0.044 / 0.045/ 0.009
PD	Neuron	56	0.672 / 0.687/ / 0.142 / 1.671	0.299 / 0.299 / 0.008	-0.012 / -0.013 / 0.008
AD	Astrocyte	66	0.746 / 0.733/ / 0.101 / 1.435	0.116 / 0.118/ 0.008	0.037 / 0.038 / 0.008
AD	Neuron	70	0.652 / 0.634/ /0.1054 / 1.3973	0.466 / 0.466/ 0.007	0.026/ 0.026 / 0.007

Tab. S15. Quantitative metrics for cell-cell communication analysis between AD and PD organoid conditions. Analysis performed using CellChat and NicheNet to predict ligand-receptor interactions and assess communication strength between cell types. Interaction weight measures the confidence score for ligand-receptor binding likelihood (0-1). AUPR (Area Under Precision-Recall curve) quantifies how well a ligand explains observed gene expression changes in receiver cells. Higher AUPR indicates stronger evidence that the ligand is functionally active in the target cell. Corrected AUPR accounts for background expectations, providing a more stringent assessment of ligand predictive capacity.

Dataset	Disease	Cell_Type	Background	Total DEGs	GWAS Genes	Overlap (n)	Overlap (%)	p-value	Overlap_Genes
AD_ Organoid	AD-like	Astrocyte	16247	398	798	18	4.52	0.68	*APOE;SOX11;MARCKS;TCF4; CLU;RPL6;DHCR24;VCAN;MEIS2;INTU* <br>*BCL11A;CST3;RPL17;AKAP9; PFKP;NUDT3;DACH1;EFNB2*

AD_ Organoid	AD-like	Neuron	16247	1028	798	58	5.64	0.15	*CADM2;SOX11;MEF2C;CNTNAP2;ARPP21;RPL6;BCL11A;MARCKS;SRRM4;VSNL1 AKAP9;MAP1B;BCHE;COX6B1;VRK2;SNCA;QKI;BRINP1;FAT4;ZC3H13 NDUFA4;PLXDC2;ANK3;TNRC6C;ARID1B;EFNB2;TMEM106B;TLE4;NRCAM;SUGT1 TMEM59;SSBP4;RAC1;SSR1;TCF4;ELAVL4;RBBP4;CELF1;MLLT3;SETD5 SULT4A1;VCAN;PFKP;MTCH2;DHCR24;APP;IGF2BP2;NRXN1;OPCML;DAB1 MTIF3;PRKCA;RBBP6;AP1S1;PGM2L1;ZNF652;USP47;GOLM1*
PD_ Organoid	PD-like	Astrocyte	15787	813	132	3	0.37	0.97	*RPS12;TOX3;CTSB*
PD_ Organoid	PD-like	Neuron	15787	956	132	7	0.73	0.67	*PTPRN2;TOX3;PDCD5;FYN;ZYG11B;NCOR1;PTPN1*

Tab. S16. Differentially expressed genes (DEGs) overlap between organoid and *GWAS* genes from AD and PD. The table shows the overlap between differentially expressed genes (DEGs) from brain organoid models and genome-wide association study (GWAS) loci for Alzheimer's disease (AD) and Parkinson's disease (PD).

Dataset	Disease	Cell Type	Overlapping Genes
Organoid + PMT + GWAS	AD	Astrocyte	*APOE, SOX11, MARCKS, RPL6, DHCR24, VCAN, RPL17, PFKP, NUDT3*
Organoid + PMT + GWAS	AD	Neuron	*RPL6, MARCKS, BCHE, COX6B1, VRK2, NDUFA4, PLXDC2, EFNB2, ELAVL4, VCAN, PFKP, DHCR24*
Organoid + PMT + GWAS	PD	Astrocyte	—

Organoid + PMT + GWAS	PD	Neuron	*PTPRN2, TOX3, FYN, ZYG11B, PTPN1*
Organoid + PMT + GWAS	AD & PD	All	—

Tab. S17. **Intersection of differentially expressed genes (DEGs) across organoid, *post-mortem* (PMT), and GWAS datasets.** Overlaps are reported without considering the directionality of expression changes between PMT and organoid study. "—" indicates no overlap.

**Dataset**	**Disease**	**Cell Type**	**Overlapping Genes**
Organoid + PMT	AD	Astrocyte	*SOX11, DHCR24, VCAN, PFKP*
Organoid + PMT	AD	Neuron	*BCHE, VCAN, PFKP, DHCR24*
Organoid + PMT	PD	Neuron	*TOX3, FYN, PTPN1*

Tab. S18. **Intersection of DEGs between organoid and *post-mortem* (PMT) datasets, including only genes with consistent directionality of differential expression.** Only cell types with overlapping DEGs are shown.
